# Electrophotocatalytic Hydroxymethylation of Azaarenes with Methanol

**DOI:** 10.1021/acs.orglett.4c02797

**Published:** 2024-08-24

**Authors:** Beatriz Quevedo-Flores, Irene Bosque, Jose C. Gonzalez-Gomez

**Affiliations:** Instituto de Síntesis Orgánica (ISO) and Departamento de Química Orgánica, Universidad de Alicante, 03080 San Vicente del Raspeig, Spain

## Abstract

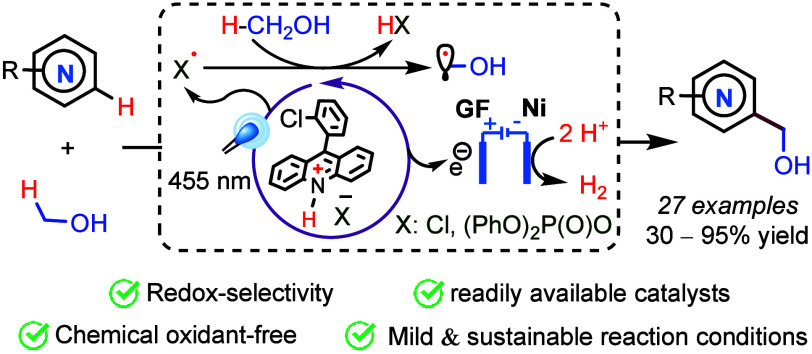

The merging of electrochemistry and photocatalysis allowed the required selectivity for the hydroxymethylation of functionalized azaarenes with methanol, including bioactive substrates. The two electrophotocatalytic protocols developed in this work address this transformation, using nontoxic and readily available reagents under mild reaction conditions with electricity as the only “sacrificial oxidant”.

Installing a hydroxymethyl group on bioactive nitrogenated heterocycles can profoundly affect their physical properties, such as solubility (log P) and their interaction with pharmacophores through hydrogen bonds. This moiety not only is found in pharmaceuticals like pirbuterol, renierol, and losartan but also offers the potential for the easy transformation of this group into other functionalities, thereby expanding the scope of drug design and synthesis.^1,2^

Hundreds of millions of tons of methanol are produced annually from different sources, including the catalytic reduction of CO_2_ (contributing to carbon neutrality). Therefore, using methanol as a C_1_ source for the direct hydroxymethylation of azaarenes is an inexpensive and sustainable approach.^3^ This transformation exemplifies a cross-dehydrogenative coupling (CDC) with the potential for late-stage functionalization of bioactive azaarenes.^4^ The weakness of C–H bonds in methanol makes their selective activation versus the O–H bonds feasible, likely involving hydrogen atom transfer (HAT) processes. The rapid reaction of this radical with protonated azaarenes was comprehensively studied by Minisci in 1985.^5^ However, years later, the same authors developed an indirect approach using ethylene glycol as the hydroxymethyl radical source to improve the selectivity of the desired transformation.^6^ Among the challenges presented in the direct hydroxymethylation are (a) the possible unproductive charge transfer between the highly nucleophilic hydroxymethyl radical and the protonated azaarene and (b) the reversibility of the radical addition due to the increased stability of the radical (Scheme 1).

**Scheme 1:**
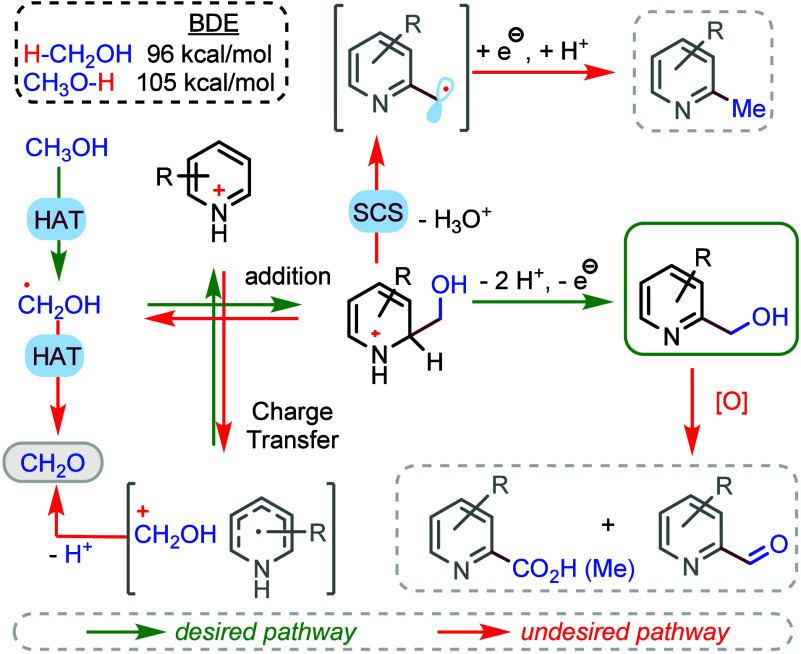
Some Challenges in the Direct Hydroxymethylation of Azaarenes with MeOH

Deprotonation of the hydroxymethyl cation or β-scission from the hydroxymethyl radical should furnish formaldehyde in competition with the desired transformation. An excess of methanol might compensate for its partial oxidation. However, the resulting hydroxymethyl derivatives are prone to further oxidation due to their benzylic structure, which commonly occurs, giving rise to aldehydes or carboxylic acid derivatives by overoxidation. In addition, the most favorable process under redox-neutral conditions is the spin-center shift, affording the corresponding methylated products.^7,8^ Moreover, polyhydroxymethylation is often observed when more than one C(sp^2^)–H bond is available due to the high nucleophilicity of the radical, which has been addressed with indirect methods such as the alkylation of *N*-methoxypyridinium derivatives.^9^

Given the mild conditions employed in photoredox catalysis to generate alkyl radicals from C–H bonds,^10^ photoinduced Minisci-like reactions have experienced significant growth in recent years.^11−13^ However, maybe due to the complications mentioned above, only a few general protocols have been reported for the photoinduced hydroxymethylation of azaarenes with methanol.^14−18^ Mild reaction conditions for this transformation have also been reported without photoactivation [e.g., Fe(II)/H_2_O_2_, rt].^19^ To the best of our knowledge, a general approach to this transformation without chemical sacrificial oxidants remains unexplored. Under the reaction conditions previously reported by our group for the photoinduced alkylation of azaarenes,^20^ we observed that MeOH gave mainly the corresponding methyl derivative (see product **31** in Scheme 3). We hypothesize that maintaining mild oxidant conditions during the reaction would minimize the spin-center shift pathway and the overoxidation to aldehydes and carboxylic acid derivatives mentioned above. Recent reports show that readily available 9-(2-chlorophenyl)acridine (**A1**) becomes photoactive with blue light (455 nm) upon protonation with HCl.^21^ Because the photoexcited acridinium should be oxidant enough for the single-electron oxidation of the chloride anion, we decided to test this *in situ* generation of chlorine radicals to promote the formation of hydroxymethyl radicals via HAT from methanol. With this in mind, we designed an electrophotocatalytic approach for the hydroxymethylation of azaarenes,^22,23^ using the acridine **A1** prephotocatalyst and inexpensive chlorohydric acid [HCl_(aq)_] at a low constant current or voltage.^24^ Moreover, our reaction design includes chloride salts as supporting electrolytes (SEs) and a source of chlorine radicals. Importantly, these salts are innocuous, abundant in diverse forms, and much more inexpensive than other SEs commonly used in electrocatalysis.

To test our hypothesis, we first examined the hydroxymethylation of 2-phenylquinoline using LiCl with aqueous HCl in MeOH and acridine **A1** as the photocatalyst. The reaction was conducted in an undivided cell at a constant current (2 mA), with irradiation with blue light-emitting diodes at room temperature. The screening of different electrodes (Table S1, entries 1–3) revealed that graphite as the anode and a Ni plate as the cathode gave optimal results after 24 h (entry 1 vs entry 4). These electrodes are rather inexpensive and deliver superior results. We examined other acids instead of HCl, which gave poorer results or no reaction (entry 5). We also found that other chloride salts can promote the reaction with good yields but less efficiently than LiCl (entry 6). Furthermore, the reaction works much better without excluding air than in an Ar or an O_2_ atmosphere (entry 7 vs entry 1), making this protocol more user-friendly. Notably, our results contrast the recently reported formylation of quinolines with methanol under HAT-mediated electrocatalytic conditions.^25^ In addition, control experiments revealed that the acid, acridine **A1**, light, and electricity were essential for the progress of the reaction (entries 8 and 9). Similar acridines were tested as precatalysts, but poorer results were obtained [**A2–A5** (Table S1, entry 10)]. It is worth noting that dehydrogenative coupling requires 2 F mol^–1^, but we have observed that the reaction is generally complete after 6 F mol^–1^. This has also been observed in previous works and could be associated with the cathodic reduction of chlorine radicals and other unproductive radical processes consuming the charge.^26^

Subsequently, the substrate scope was investigated under optimal electrophotocatalytic conditions for a range of azaarenes (Figure 1). 2-Alkyl quinolines, containing different halides or ester groups at C6 and/or C7, provided the corresponding 4-hydroxymethyl products (**2**–**6**) in good yields (60–89%). Additionally, 4-substituted quinolines afforded 2-hydroxymethyl products in moderate-to-excellent yields (**7**–**11**, 40–95%), with tolerance for halide, alkyl, aryl, and alkyne substituents. Notably, 3-methylquinoline and quinoline 8-sulfonic acid afforded the corresponding 2,4-dihydroxymethyl products in good yields (**12** in 74% yield and **13** in 50% yield, respectively). In the former case, it could be a result of a similar steric hindrance at C2 and/or C4, while in the latter case, it must be due to the strong electron-withdrawing effect of the sulfonic acid, which increases the reactivity of the azaarene with the nucleophilic hydroxymethyl radical. Interestingly, selective monosubstitution was achieved with 2,2′-biquinoline (**14**, 53%), and the protocol was successfully applied to a quinoline–menthol hybrid molecule (**15**, 44%).

**Figure 1:**
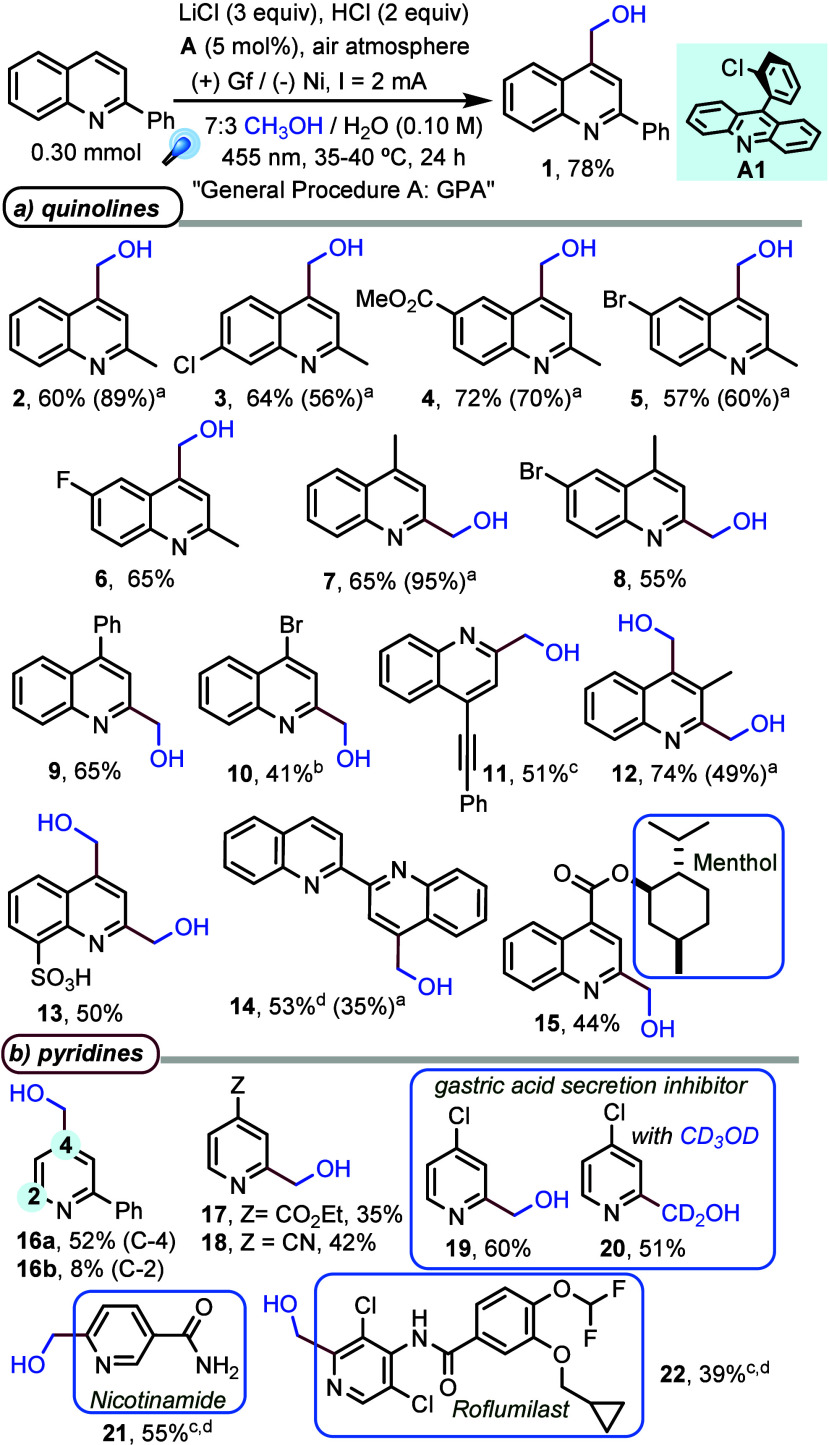
Substrate scope obtained with LiCl/HCl. Yields for isolated pure products are given. ^a^Current of 4 mA. ^b^Contaminated with 12% 4-chloro derivative. ^c^Cell voltage of 1.5 V. ^d^For 48 h.

2-Phenylpyridine gave monoalkylation, the 4-hydroxymethyl product being the major one, which is in line with the preferential attack of a nucleophilic radical at C4 of a pyridinium ion, the atom with the largest coefficient in the LUMO (C4).^27^ Monoalkylation was also observed for C4-substituted pyridines, affording compounds **17**–**20** in moderate to good yields. We found this quite interesting because, under classical (and harsher) Minisci conditions, mixtures of mono- and dialkylated products are commonly obtained, which are difficult to separate.^28^ In particular, we obtained compound **19** in 60% yield, while a 20–30% yield is reported using (NH_4_)_2_S_2_O_8_ as the sacrificial oxidant and thermal activation.^9^ This product is an inhibitor of gastric secretion, and we could prepare its deuterated analogue from CD_3_OD in satisfactory yield (**20**, 51%). Remarkably, vitamin B_3_ gave 6-(hydroxymethyl)nicotinamide **21** with excellent regio- and chemoselectivity. Furthermore, roflumilast, an inhibitor of phosphodiesterase-4 used as medication in severe chronic obstructive pulmonary disease, afforded monoalkylated product **22** in synthetically useful yield, exhibiting a good tolerance to various functionalities. Notably, quinaldine and lepidine gave the products in better yields with a higher current intensity (products **2** and **7**). Still, we have checked for five other products (**3**–**5**, **12**, and **14**), but similar or better results were obtained at 2 mA. Moreover, other more sensitive substrates gave the best results, fixing the cell voltage at 1.5 V (products **11**, **21**, and **22**). In all of these cases, the observed current intensity was <2 mA (details in the Supporting Information for each product). A list of substrates that failed to give the desired product in >25% yield is given in Table S3.

For isoquinoline, we observed the incorporation of a chlorine radical under the reaction conditions shown in Figure 1 (Table S3). Therefore, after new reaction conditions in the absence of chlorides had been screened (Table S2), Bu_4_NBF_4_ was the optimal supporting electrolyte when diphenyl hydrogen phosphate was used instead of HCl to generate the HAT catalyst.^29^ Under these conditions, five isoquinolines were selectively hydroxymethylated at C1, including the acetyl derivative of Fasudil, a potent Rho-kinase inhibitor and vasodilator (Figure 2, products **23**–**27**). In addition, the phenanthridine has shown singular reactivity. When the substrate was submitted to the reaction conditions of GPA (chlorine-mediated), methyl derivative **28** was obtained in good yield. Instead, the reaction conditions of GPB, but fixing the cell voltage at 1.5 V, afforded formyl derivative **29** in good yield. We suspected that the aerobic O_2_ could facilitate the overoxidation, but similar results were obtained under an Ar atmosphere. Moreover, using CD_3_OH, we obtained deuterated formyl derivative **30** in very good yield. The dichotomy found in the reactivity of phenanthridine reveals that fine-tuning the reaction conditions is necessary to obtain the desired hydroxymethylation (redox selectivity) without further reduction (methylation) or further oxidation (formylation).

**Figure 2:**
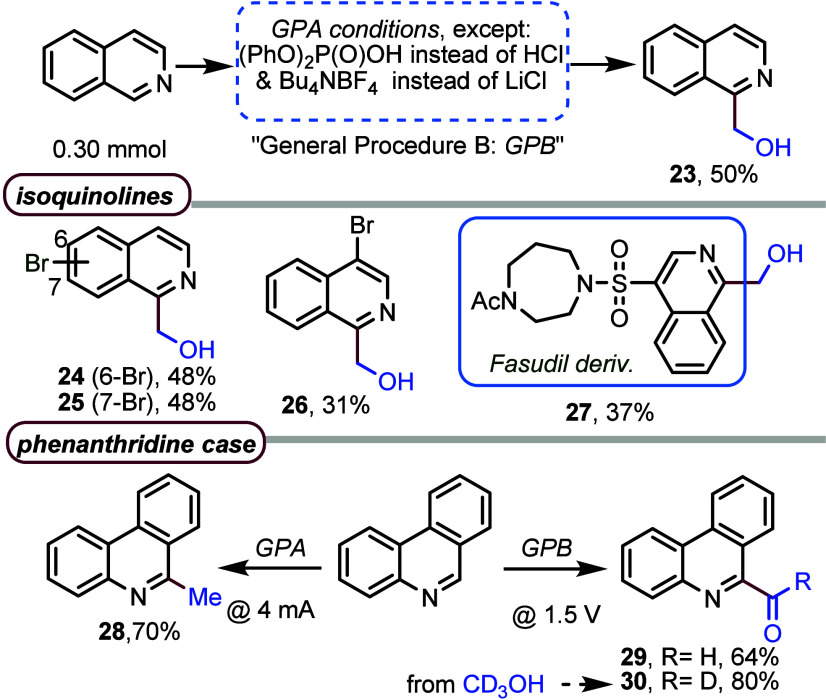
Substrate scope in the absence of chlorides. Yields for isolated pure products are given.

In preliminary mechanistic investigations, we have observed that radical inhibitors such as TEMPO and 1,1-diphenylethene completely stifle the reaction, and we were able to detect by LC-MS adducts **Ad1** and **Ad2** with the hydroxymethyl radical (Scheme 2A; details in the Supporting Information). Moreover, CuCl_2_, a single-electron scavenger, also efficiently inhibited the reaction. In an attempt to trap the chlorine radical, we submitted the 1,1-diphenylethene to the reaction conditions of GPA (Scheme 2B), observing the formation of **Ad3** by LC-MS and ^1^H NMR. Considering our mechanistic observations (including control experiments and the reactivity of substrates) and literature precedent, we propose a plausible mechanism (Scheme 2C). Photoinduced electron transfer (PET) between the activated acridinium catalyst (*E*_p/2_ + 2.19 V vs SCE)^21^ and chloride anion should form chlorine radicals [*E*_p/2_ > 1.65 V vs SCE, in the reaction medium (Figure S10, right)]. The electrochemical oxidation of the chloride anion is unlikely because we have checked that when the current is kept at 2 mA in the reaction mixture, the anodic potential ranges from 1.20 to 1.50 V versus a Ag/AgCl reference electrode. This is in accordance with the failure of the reaction without the photocatalyst or light (Table S1, entry 9). HAT from methanol to chlorine atoms is plausible, considering the corresponding bond dissociation energies.^30^ The addition of the hydroxymethyl radical generated to the protonated azaarene must be followed by deprotonation and single-electron oxidation, likely in solution by **[AH**^**+**^]*, but anodic oxidation is also possible. A crucial element of our reaction design is the anodic oxidation of AH^•^ at a low oxidation potential [*E*_1/2_ = −0.67 V vs SCE (Figure S11, right)], revitalizing the photocatalyst under mild oxidation conditions. We cannot completely rule out the possibility that aerobic O_2_ contributes to turning over the photocatalyst (or to the final oxidation steps) in a Fenton-like process. However, we could not detect the formation of H_2_O_2_ with a KI test, and the reactions worked poorly under a pure O_2_ atmosphere for **s1** (Table S1, entry 7) or nothing for isoquinoline (Table S2, entry 4).

**Scheme 2:**
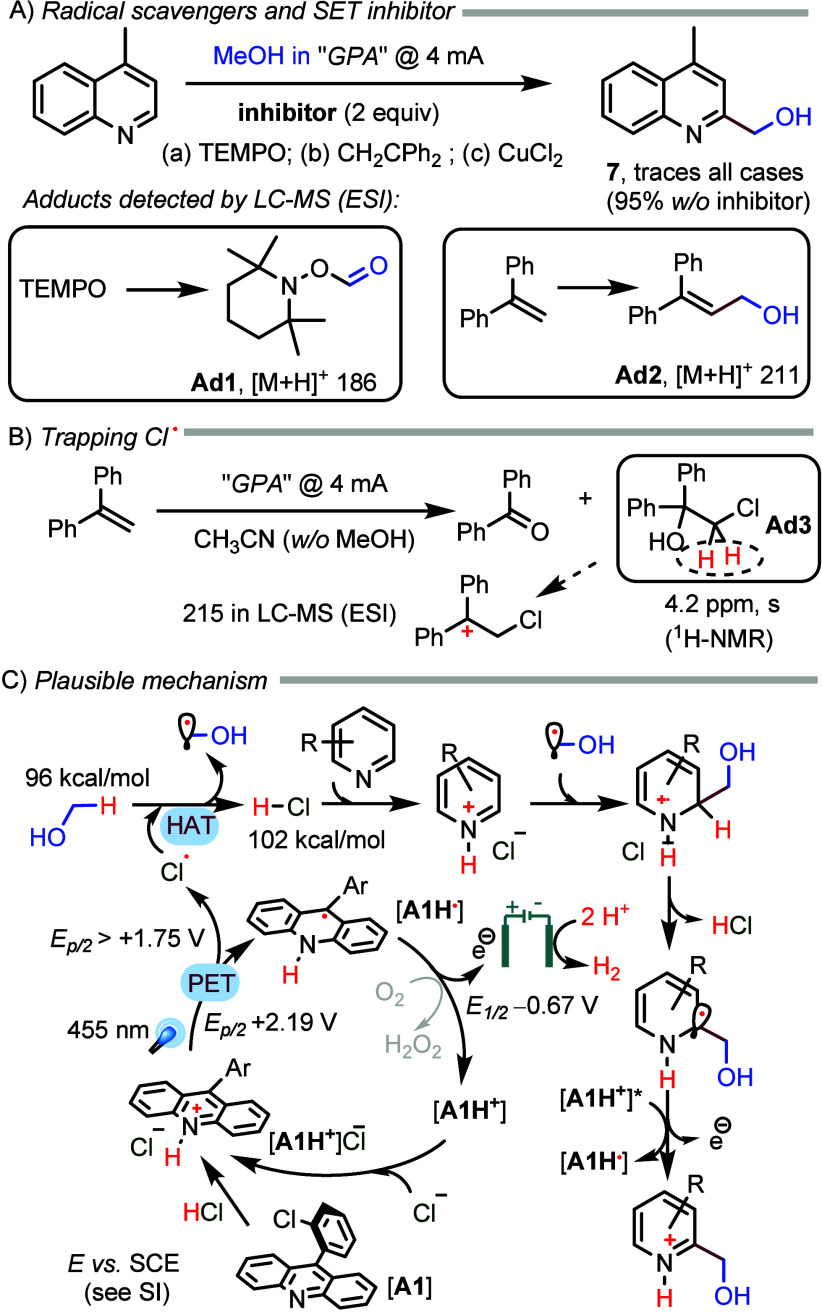
Mechanistic Investigations and Proposal

As mentioned above, we have observed the methylation of azaarenes with methanol under photocatalytic conditions,^19^ illustrated for product **31** in Scheme 3A, while formylation has recently been reported under electrocatalytic conditions.^25^ We have also verified that hydroxymethyl compound **1** can be electrochemically oxidized to **32**, and its reduction to **31** has been reported under photocatalytic conditions.^15^ Therefore, combining electrochemical and photochemical activation modes provides a redox selectivity different from that obtained with each activation mode.

**Scheme 3:**
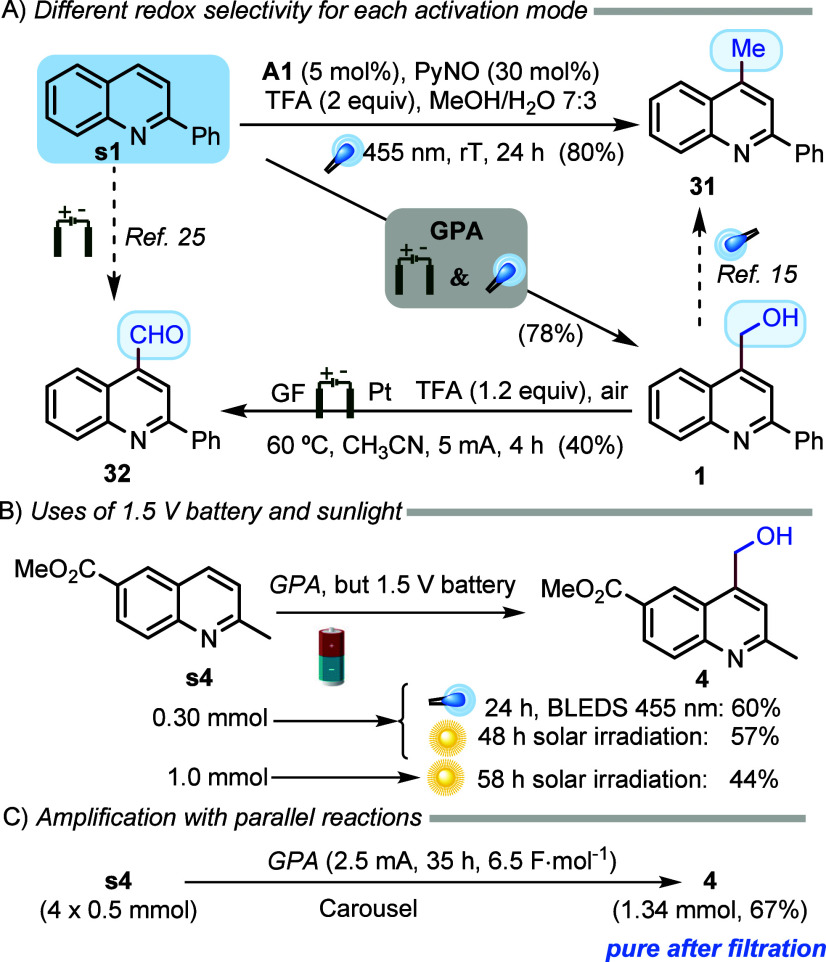
Selectivity and Applicability

To make the protocol more user-friendly, we demonstrated that the hydroxymethylation of **s4** can be performed in good yields using an inexpensive 1.5 V battery, even under solar irradiation without stirring (Scheme 3B). Moreover, four parallel hydroxymethylations of **s4** were conducted using a carrousel (details in the Supporting Information) to obtain 1.34 mmol of product **4**, which precipitated from EtOAc after the workup and was obtained in pure form after filtration (Scheme 3C). It is worth noting that we obtained a yield for isolated pure product **4** (67%) similar to that obtained at a 0.30 mmol scale (72%)

In conclusion, we developed an electrophotocatalytic protocol for the hydroxymethylation of azaarenes with methanol. We demonstrated that merging photochemistry and electrochemistry provides a selectivity different from that obtained with each activation mode. This approach relies on readily available acridine **A1** as the organophotocatalyst and LiCl/HCl(aq) to generate chlorine atoms for one protocol (GPA) or diphenyl hydrogen phosphate for isoquinolines (GPB) as HAT reagents.

## Data Availability

The data underlying this study are available in the published article and its Supporting Information.

## Supporting Information Available

Experimental procedures and optimization, mechanistic studies, and full characterization (including NMR spectra) of all products (PDF)
