# Supplementary material for: Electrophotocatalytic Hydroxymethylation of Azaarenes with Methanol
Source: Org Lett. 2024 Aug 24;26(35):7447–51. doi: 10.1021/acs.orglett.4c02797 (PMC11385437; doi:10.1021/acs.orglett.4c02797)
Supplement: Supplementary file 1 — ol4c02797_si_001.pdf [file ol4c02797_si_001.pdf]

Supporting information for

Electrophotocatalytic hydroxymethylation of azaarenes with methanol

Beatriz Quevedo-Flores, Irene Bosque,* Jose C. Gonzalez-Gomez*

Instituto de Síntesis Orgánica (ISO) and Departamento de Química Orgánica
Universidad de Alicante, Apdo. 99, 03080 Alicante, Spain

TABLE OF CONTENTS

MATERIALS AND METHODS 4
GENERAL METHODS 5
Preparation of acridine catalysts 5
Preparation of 9-(2-Chlorophenyl)acridine (A1) 5
Preparation of bis(triphenylphosphine)palladium chloride 5
Preparation of starting materials 6
Synthesis of 4-phenylquinoline 6
Synthesis of 4-(phenylethynyl)quinoline 6
Synthesis of (1*S**,2*R**,5*S**)-2-isopropyl-5-methylcyclohexyl quinoline-4-carboxylate 7
Synthesis of *N*-acetyl fasudil 7
OPTIMIZATION OF REACTION CONDITIONS 8
GENERAL PROCEDURES 10
General procedure A (GPA): 10
GPB: 10
AMPLIFICATION WITH PARALLEL REACTIONS 11
USE OF A 1.5 V BATTERY AS A POWER SOURCE 12
USE OF 1.5 V BATTERY UNDER SUNLIGHT IRRADIATION 13
REACTION AT 1 MMOL USING A 1.5 V BATTERY UNDER SUNLIGHT IRRADIATION 14
MECHANISTIC STUDIES 15
Addition of TEMPO 15
Addition of 1,1-Diphenylethylene 16
Addition of $CuCl_2$ 16
Trapping Cl radical with 1,1-Diphenylethylene 17
Cyclic Voltammetry (CV) measurements 18
CHARACTERIZATION OF PRODUCTS 20
(2-Phenyl-4-yl)methanol (1): 20
(2-Methyl-4-yl)methanol (2): 20
(2-Methyl-7-chloroquinoline -4-yl)methanol (3): 20
Methyl 4-(hydroxymethyl)-2-methylquinoline-6-carboxylate (4): 21
(2-Methyl-6-bromoquinoline -4-yl)methanol (5): 21
(2-Methyl-6-fluoroquinoline -4-yl)methanol (6): 21
(4-Methylquinolin-2-yl)methanol (7): 22
(4-Methyl-6-bromoquinolin-2-yl)methanol (8): 22

(4-Phenylquinolin-2-yl)methanol (9): 23
(4-Bromoquinolin-2-yl)methanol (10): 23
(4-(Phenylethynyl)quinolin-2-yl)methanol (11): 23
(3-Methylquinoline-2,4-diyl)dimethanol (12): 24
2,4-Bis(hydroxymethyl)quinoline-8-sulfonic acid (13): 24
[2,2'-Biquinolin]-4-ylmethanol (14): 24
(1S*,2R*,5S*)-2-Isopropyl-5-methylcyclohexyl 2-(hydroxymethyl)quinoline-4-carboxylate (15): 25
(2-Phenylpyridin-4-yl)methanol (16a): 25
Ethyl 2-(hydroxymethyl)isonicotinate (17): 26
2-(Hydroxymethyl)isonicotinonitrile (18): 26
(4-Chloropyridin-2-yl)methanol (19): 27
(4-Chloropyridin-2-yl)methan-D_2-ol (20): 27
6-(Hydroxymethyl)nicotinamide (21): 27
3-(cyclopropylmethoxy)-N-(3,5-dichloro-2-(hydroxymethyl)pyridin-4-yl)-4-(difluoromethoxy)benzamide (22): 28
Isoquinolin-1-ylmethanol (23): 28
(6-Bromoisoquinolin-1-yl)methanol (24): 28
(7-Bromoisoquinolin-1-yl)methanol (25): 29
(4-Bromoisoquinolin-1-yl)methanol (26): 29
1-(4-((1-(hydroxymethyl)isoquinolin-4-yl)sulfonyl)-1,4-diazepan-1-yl)ethan-1-one (27): ... 30
Phenanthridine-6-methyl (28): 30
Phenanthridine-6-carbaldehyde (29): 30
Phenanthridine-6-carbaldehyde-D (30): 31
4-Methyl-2-phenylquinoline (31): 31
2-Phenylquinoline-4-carbaldehyde (32): 32
UNSUCCESSFUL SUBSTRATES 33
NMR SPECTRA OF SYNTHESIZED COMPOUNDS 34
REFERENCES 69

MATERIALS AND METHODS

Solvents and reagents: Unless otherwise stated, all solvents and commercially available reagents were purchased in reagent grades and used without further purification.

General methods: All air- and moisture-insensitive reactions were carried out under an ambient atmosphere and monitored by thin-layer chromatography (TLC) and Gas Chromatography-mass spectroscopy (GC-MS). TLCs were performed on silica gel 60 F_{254}, using aluminum plates and visualized by exposure to ultraviolet light. Flash column chromatography (FC) was performed using Merck silica gel 60 (230–400 mesh). Yields refer to purified compounds unless otherwise stated.

Setup: The photoelectrocatalytic reactions were conducted using IKA ElectraSyn 2.0 Pro equipment, with undivided cell and IKA electrodes (5x1x0.1 cm), fixing the current intensity or the voltage and using the corresponding vials. Each reaction mixture (rxm) was irradiated with one 18 W EvoluChem LEDs 450PF. The irradiance was measured by placing the lamp at 5 cm of the Electrasyn cell with a Blue-Wave UVIS-25 spectrometer (32-34 W $\times m^{-2}$). For light spectrum and other details, see: https://www.hepatochem.com/product/hck1012-xx-002/).

Analytical Information:

NMR spectra were recorded at 300 or 400 MHz for ^{1}H and 75 or 101 MHz for ^{13}C, using $CDCl_3$, MeOD-d_4 or DMSO-d_6 as solvent. For ^{1}H-NMR in $CDCl_3$, TMS was used as an internal standard (0.00 ppm). For ^{1}H-NMR in DMSO-d_6, the residual signal was used as the internal standard (2.50 ppm). Data are reported as (s = singlet, d = doublet, t = triplet, q = quartet, m = multiplet or unresolved, brs = broad signal, coupling constant(s) in Hz, integration). ^{13}C-NMR spectra were recorded with ^{1}H-decoupling at 101 MHz and referenced to $CDCl_3$ at 77.16 ppm or DMSO-d_6 at 39.52 ppm.

LRMS were obtained using an Agilent 5977B mass spectrometer with a quadrupole analyzer coupled with a gas chromatographer Agilent 8890. The oven temperature was: 3 min at 80 ºC, then 20 ºC/min ramp until 300 ºC, then 3 min at 300 ºC.

HRMS analyses were carried out in the electron impact mode (EI) at 70 eV using a quadrupole mass analyzer or by Q-TOF using electrospray ionization (ESI) mode.

GENERAL METHODS

Preparation of acridine catalysts

Preparation of 9-(2-Chlorophenyl)acridine (A1)

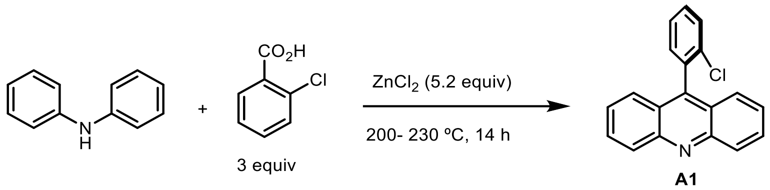

Following a procedure previously reported, 9-(2-Chlorophenyl)acridine (**A1**) was prepared.[1] Acridines **A2** to **A5** were prepared following the same reference. A sand bath was used for heating.

Preparation of bis(triphenylphosphine)palladium chloride

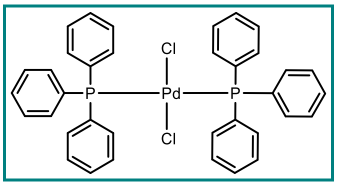

This catalyst was prepared according to a reported procedure.[2]

$PdCl_2$ (53 mg, 0.30 mmol) was added to an oven-dried Schlenck flask, followed by dry THF (3 mL) and LiCl (26 mg, 0.6 mmol) under Ar atmosphere. The reaction mixture was stirred under an Ar atmosphere for 5 min at room temperature (25 ºC). After this time, PPh_3 (157 mg, 0.60 mmol) was added to the resulting grey suspension, and the reaction mixture (rxm) was stirred for at least 2 h at room temperature. The formed pale-yellow suspension remained under the Ar atmosphere.

Preparation of starting materials

All substrates examined in this study were commercially available except for the following ones:

Synthesis of 4-phenylquinoline

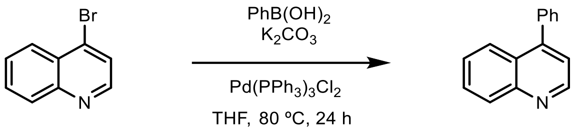

Following a reported protocol,[3] K_2CO_3 (0.5 mL, 2M) was added to a solution of 4-bromoquinoline (104 mg, 0.5 mmol) and phenylboronic acid (73 mg, 0.6 mmol) in THF dry (1 mL). The mixture was stirred at room temperature for 30 min under Ar atmosphere. $Pd(PPh_3)_2Cl_2$ (5 mol%) was added to the rxm and stirred at 80 ºC (sand bath) for 16 h. After reaching rT, extraction with EtOAc (3 x 10 mL) was followed by washing with brine (2 x 5 mL). The organic layers were collected and dried over $MgSO_4$, filtered, and concentrated *in vacuo* to give an orange oil. The crude was purified by FC using from 0% to 10% EtOAc in *n*-hexane as the eluent to give a white solid (90 mg, 0.44 mmol, 88%).

Synthesis of 4-(phenylethynyl)quinoline

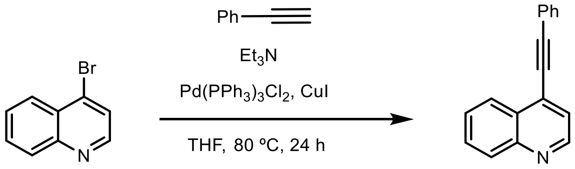

Following a reported protocol,[4] 4-bromoroquinoline (400 mg, 2.0 mmol), the palladium catalyst (5 mol%) and CuI (5 mol %) were added to a 20-mL-pressure tube. TEA (0.80 mmol, 4 equiv.) was degassed and added to the rxm, followed by a solution of phenylacetylene (0.32 mL, 3 mmol, 1.5 equiv.) in THF (16 mL). The rxm was put under the Ar atmosphere and stirred at 80 ºC (sand bath) for 24 h. After evaporation of volatiles, K_2CO_3 (aq. sat.,10 mL) was added, and the product was extracted with EtOAc (2x20 mL). The combined organic layers were dried with $MgSO_4$ and concentrated under vacuum. The crude product was purified by FC using from 0% to 30% EtOAc in *n*-hexane as the eluent to give a black oil (330 mg, 1.38 mmol, 70%).

Synthesis of (1*S**,2*R**,5*S**)-2-isopropyl-5-methylcyclohexyl quinoline-4-carboxylate

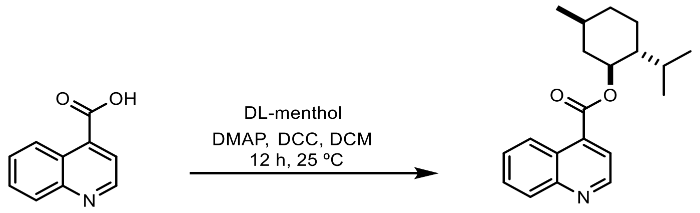

Following a reported protocol,[5] quinoline-4-carboxylic acid (415 mg, 2.4 mmol, 1.2 equiv.), menthol (312 mg, 2 mmol, 1 equiv.) and DMAP (25 mg, 0.2 mmol, 10 mol%) were added to a 50 mL round-bottomed flask, followed by CH_2Cl_2 (10 mL). Then, a solution of DCC (495 mg, 2.4 mmol, 1.2 equiv.) in CH_2Cl_2 (10 mL) was slowly added at room temperature. The rxm was stirred at room temperature for 12 h. At this point, the urea was filtered out, and the solution was concentrated *in vacuo*. Then, the residue was diluted with water and extracted with CH_2Cl_2 (3 x 20 mL). The combined organic layers were dried over $MgSO_4$ and filtered. The solvent was removed under reduced pressure, and the residue was purified by FC using a gradient from 0% to 30% of EtOAc in *n*-hexane as the eluent. The product was obtained as a white oil (500 mg, 1.6 mmol, 80%).

Synthesis of *N*-acetyl fasudil

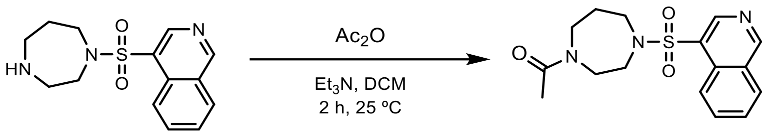

This compound was prepared according to a reported procedure.[6] The product was obtained as a white oil (55 mg, 0.16 mmol, 55%).

OPTIMIZATION OF REACTION CONDITIONS

Table S1: Hydroxymethylation of 2-phenylquinoline

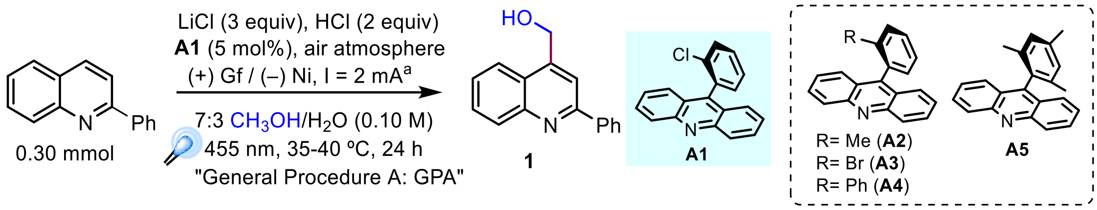

Entry	Deviation from the GPA	Yield (%)[b]
1	none	80 (78)[c]
2	Gf / Pt / Ni foam as cathode	10 / 40 / 60
3	Glassy Carbon as anode	55
4	16 h instead of 24 h	50
5	HNO_3 / $(PhO)_2P(O)OH$ / TFA, instead of HCl	40 /10 / 0
6	NaCl / KCl, instead of LiCl	67 /72
7	Argon atmosphere[d] / O_2 atmosphere[e]	35 / 18
8	*w/o* acid or *w/o* electricity	0
9	*w/o* **A1** or *w/o* light	0
10	**A2** / **A3** / **A4** / **A5** instead of **A1**	38 / 55 / 64 / 63

[a]The anodic potential *vs.* Ag/AgCl (0.10 M KCl) ranged from 1.20 V to 1.50 V over 24 h. [b]GC yield based on remaining SM without calibration. [c]Isolated pure product. [d]Three cycles of freeze-pump-thaw with Ar, then an Ar balloon connected. [e]Three cycles of freeze-pump-thaw with O_2, then an O_2 balloon connected.

The anodic potential *vs.* Ag/AgCl (0.10 M KCl) was measured by introducing the reference electrode in the reaction mixture and using an external multimeter. The values were taken at 30 min (1.5 V), 1 h (1.49 V), 16 h (1.25 V), and 24 h (1.49 V). Importantly, before switching on the light, the anodic potential *vs.* Ag/AgCl was 1.03 V, indicating that Cl_2 is not electrochemically generated in the absence of light (*vide infra* Figure S9, in accordance with entry 9 of Table S1).

Table S2: Hydroxymethylation of isoquinoline.

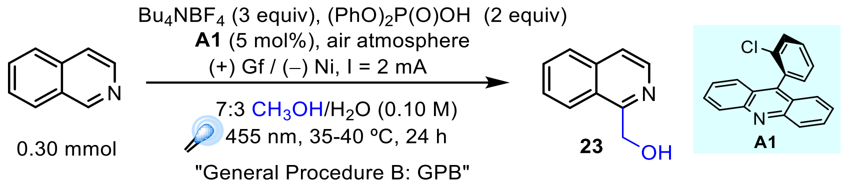

**Entry**	**Deviation from the GPB**	**Yield (%)**[a]
1	none	56 (51)[b]
2	HNO_3 / H_3PO_4, instead of $(PhO)_2P(O)OH$	30 / 0
3	Bu_4NPF_6/ $LiClO_4$, instead of Bu_4NBF_4	36 / 30
4	Argon atmosphere[c] / O_2 atmosphere[d]	40[e] / 0[f]
5	*w/o* acid or *w/o* electricity	0
6	*w/o* **A1** or *w/o* light	0

[a] GC yield based on remaining SM without calibration. [b] Isolated pure product. [c] Three cycles of freeze-pump-thaw with Ar, then an Ar balloon connected. [d] Three cycles of freeze-pump-thaw with O_2, then an O_2 balloon connected. [e] 10% of 1-methylisoquinoline was also obtained. [f] 12% of aldehyde in GCMS.

GENERAL PROCEDURES

General procedure A (GPA):

In a 10 mL ElectraSyn vial -equipped with a stirring bar- was added the azaarene (0.30 mmol), LiCl (38 mg, 0.90 mmol, 3 equiv., in 1.2 mL of H_2O) and 9-(2-chlorophenyl)acridine (**A1**, 4.5 mg, 0.015 mmol, 5 mol%), followed by MeOH (4.2 mL) and HCl (1 M, 0.6 mL, 2 equiv.). All reagents were added in open-air conditions. The electrodes Gf (+)/Ni (-) were then inserted, and the reaction was stirred under galvanostatic conditions (2 mA) using ElectraSyn 2.0 while irradiated with blue LEDs (455 nm) at a distance of 5 cm for 24 h at room temperature (30-35ºC). Once this time elapsed, a saturated solution of K_2CO_3 was added (3 mL), and the organic phase was extracted with EtOAc (3x10 mL). After collecting and drying the organic phases over $MgSO_4$, the solvent was removed under reduced pressure, and the residue was purified by FC.

GPB:

In a 10 mL ElectraSyn vial -equipped (Figure S1) with a stirring bar- was added the azaarene (0.30 mmol), Bu_4NBF_4 (294 mg, 0.9 mmol, 3 equiv.), diphenyl phosphate (150 mg, 0.6 mmol, 2 equiv.) and 9-(2-chlorophenyl)acridine (**A1**, 4.5 mg, 0.015 mmol, 5 mol%), followed by H_2O (1.8 mL) and MeOH (4.2 mL). All reagents were added in open-air conditions. The electrodes Gf(+)/Ni (-) were then inserted, and the reaction was stirred under galvanostatic conditions (2 mA) using ElectraSyn 2.0 while irradiated with blue LEDs (455 cm) at a distance of 5 cm for 24 h at room temperature (30-35ºC). Once this time elapsed, a saturated solution of K_2CO_3 was added, and the organic phase was extracted with EtOAc (3x10 mL). After collecting and drying the organic phases over $MgSO_4$, the solvent was removed under reduced pressure, and the residue was purified by FC.

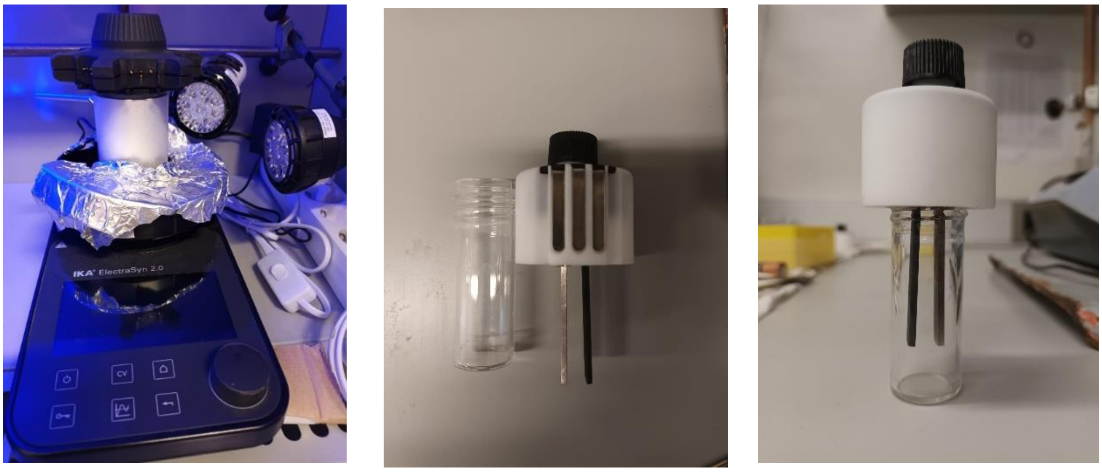

Figure S1: Equipment and electrodes used in the electrophotocatalytic reactions.

AMPLIFICATION WITH PARALLEL REACTIONS

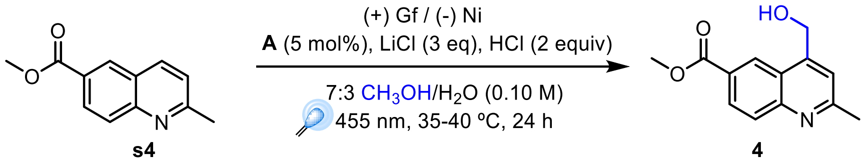

Four reactions were run simultaneously using a carrousel designed for the IKA ElectraSyn 2.0 Pro equipment (Figure S2). Each reaction was set in a 10 mL vial equipped with a stirring bar, which was fed with methyl-2-methylquinoline-6-carboxylate (0.5 mmol), LiCl (64 mg, 1.5 mmol, 3 equiv., in 2 mL of H_2O) and 9-(2-chlorophenyl)acridine (**A1**, 7.5 mg, 5 mol%), followed by MeOH (7 mL) and HCl (1 M, 1 mL, 2 equiv.). The electrodes Gf(+)/Ni (-) were then inserted, and the reaction was stirred under galvanostatic conditions (2.5 mA) for 35 h (6.5 F· mol^{-1}) at room temperature (30-35 ºC). Once this time elapsed, all the rxms were collected and washed with a saturated solution of K_2CO_3 (1x10 mL). The aqueous phase was extracted with EtOAc (3x 50 mL), and the collected organic layers were dried over $MgSO_4$. After removal of the solvent under reduced pressure, EtOAc was added, and the precipitated solid was filtered and washed with more EtOAc to give product **4** as a pure white solid (310 mg, 1.34 mmol, 67%).

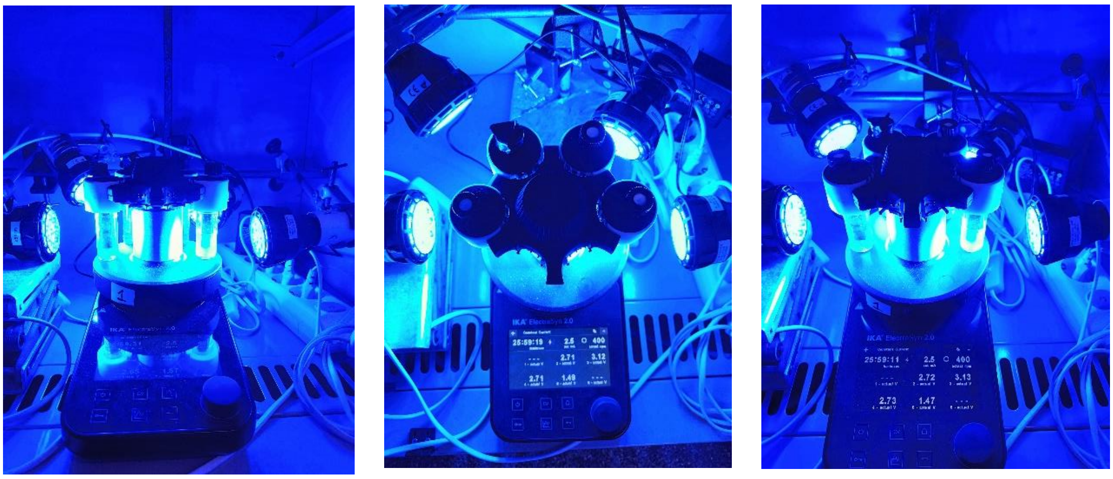

Figure S2: Set up used for the amplification.

USE OF A 1.5 V BATTERY AS A POWER SOURCE

In a 10 mL vial -equipped with a stirring bar- was added methyl-2-methylquinoline-6-carboxylate (**s4**, 60 mg, 0.30 mmol), LiCl (38 mg, 0.90 mmol, 3 equiv, in 1.2 mL of H_2O) and 9-(2-chlorophenyl)acridine (**A1**, 4.5 mg, 0.015 mmol, 5 mol%), followed by MeOH (4.2 mL) and HCl (1 M, 0.6 mL, 2 equiv). The electrodes Gf(+)/Ni (-) were then inserted, and the reaction was stirred under 1.5 V connected by a battery while irradiated with blue LEDs (455 nm) for 24 h at room temperature (30-35ºC). Once this time elapsed, a saturated solution of K_2CO_3 was added (3 mL), and the organic phase was extracted with EtOAc (3x10 mL). After collecting and drying the organic phases over $MgSO_4$, the solvent was removed under reduced pressure. EtOAc was added, and the precipitated solid was filtered and washed with more EtOAc to give product **4** as a white solid (42 mg, 60%).

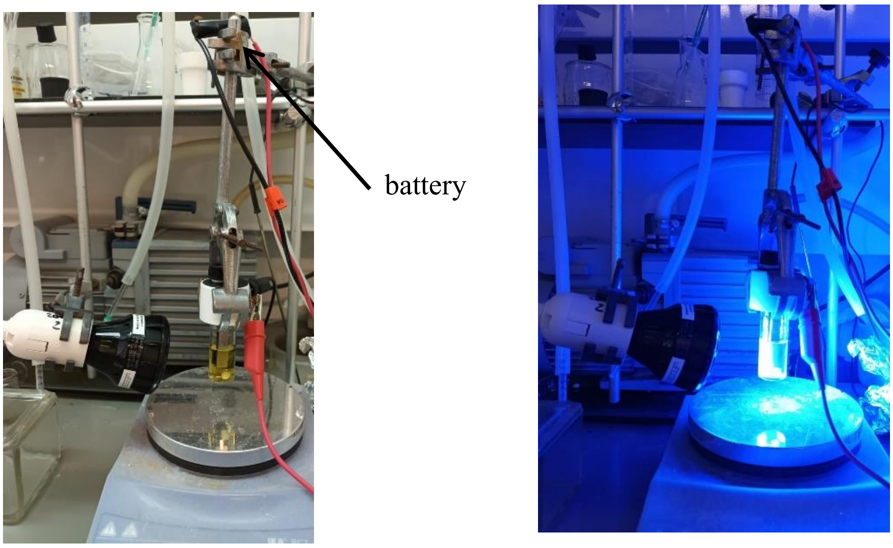

Figure S3: Reaction performed using a 1.5 V battery, and blue LEDs.

USE OF 1.5 V BATTERY UNDER SUNLIGHT IRRADIATION

In a 10 mL vial was added methyl-2-methylquinoline-6-carboxylate (60 mg, 0.30 mmol), LiCl (38 mg, 0.90 mmol, 3 equiv, in 1.2 mL of H_2O) and 9-(2-chlorophenyl)acridine (**A1**, 4.5 mg, 0.015 mmol, 5 mol%), followed by MeOH (4.2 mL) and HCl (1 M, 0.6 mL, 2 equiv). The electrodes Gf(+)/Ni (-) were then inserted, and the reaction was stirred under 1.5 V connected by a battery while irradiated with the sun for 48 h without stirring (about 4 days in total, Figure S4). Once this time elapsed, a saturated solution of K_2CO_3 was added (3 mL), and the organic phase was extracted with EtOAc (3x10 mL). After collecting and drying the organic phases over $MgSO_4$, the solvent was removed under reduced pressure. EtOAc was added, and the precipitated solid was filtered and washed with more EtOAc to give product **4** as a white solid (40 mg, 57%).

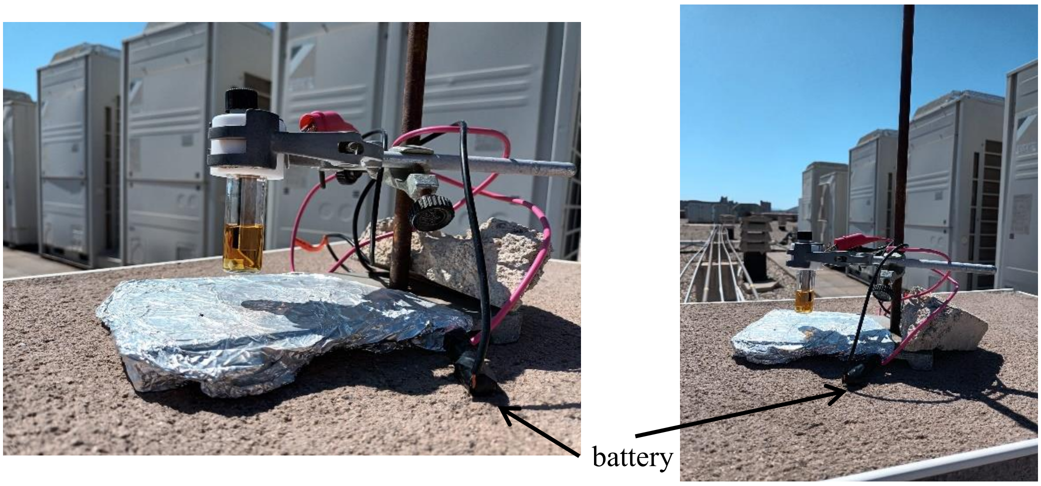

Figure S4: Reaction performed using a 1.5 V battery under solar irradiation.

REACTION AT 1 MMOL USING A 1.5 V BATTERY UNDER SUNLIGHT IRRADIATION

In a 20 mL vial was added methyl-2-methylquinoline-6-carboxylate (201 mg, 1 mmol), LiCl (127 mg, 3 mmol, 3 equiv, in 4 mL of H_2O) and 9-(2-chlorophenyl)acridine (**A1**, 15 mg, 0.05 mmol, 5 mol%), followed by MeOH (14 mL) and HCl (1 M, 2 mL, 2 equiv). The electrodes Gf(+)/Ni (-) were then inserted, and the reaction was stirred under 1.5 V connected by a battery while irradiated with the sun for 58 h without stirring (about 5 days in total, Figure S5). Once this time elapsed, a saturated solution of K_2CO_3 was added (3 mL), and the organic phase was extracted with EtOAc (3x10 mL). After collecting and drying the organic phases over $MgSO_4$, the solvent was removed under reduced pressure. EtOAc was added, and the precipitated solid was filtered and washed with more EtOAc to give product **4** as a white solid (102 mg, 44%).

(a) (b)

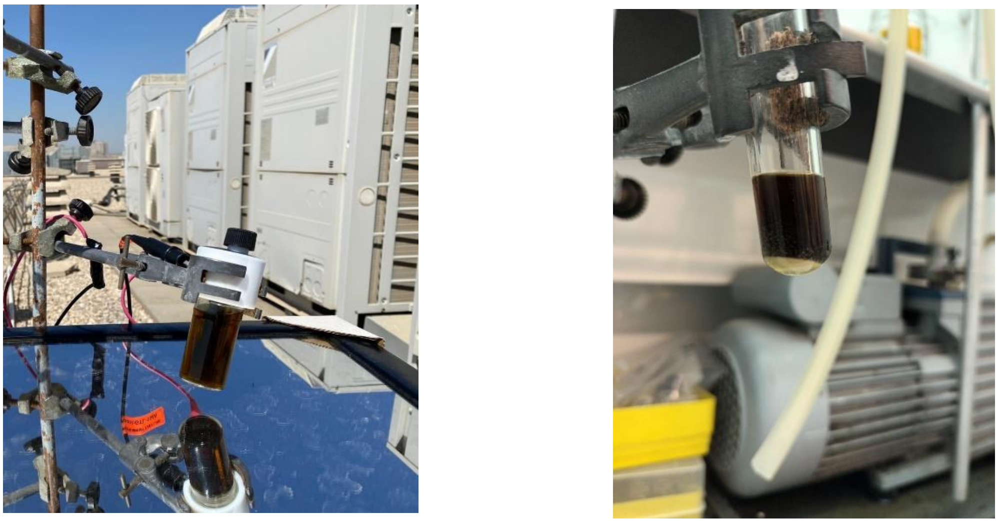

Figure S5: (a) Reaction performed using a 1.5 V battery under sunlight irradiation, using a mirror. (b) Precipitated product upon workup.

MECHANISTIC STUDIES

Addition of TEMPO

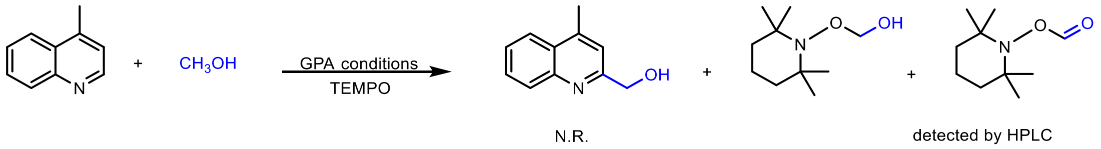

In a 10 mL vial equipped with a stirring bar was added lepidine (0.30 mmol), LiCl (38 mg, 0.90 mmol, 3 equiv., in 1.2 mL of H_2O), 9-(2-chlorophenyl)acridine (**A1**, 4.5 mg, 0.015 mmol, 5 mol%) and TEMPO (94 mg, 0.6 mmol, 2 equiv.), followed by MeOH (4.2 mL) and HCl (1 M, 0.6 mL, 2 equiv.). The electrodes Gf(+)/Ni (-) were then inserted, and the reaction was stirred under galvanostatic conditions (2 mA) for 24 h at room temperature. Once the time elapsed, the reaction mixture was analyzed by HPLC (ES+). While the hydroxymethyl derivative was not observed, an adduct of TEMPO with formaldehyde (**Ad1** in Figure S6) was detected, likely from trapping the hydroxymethyl radical and further oxidation.

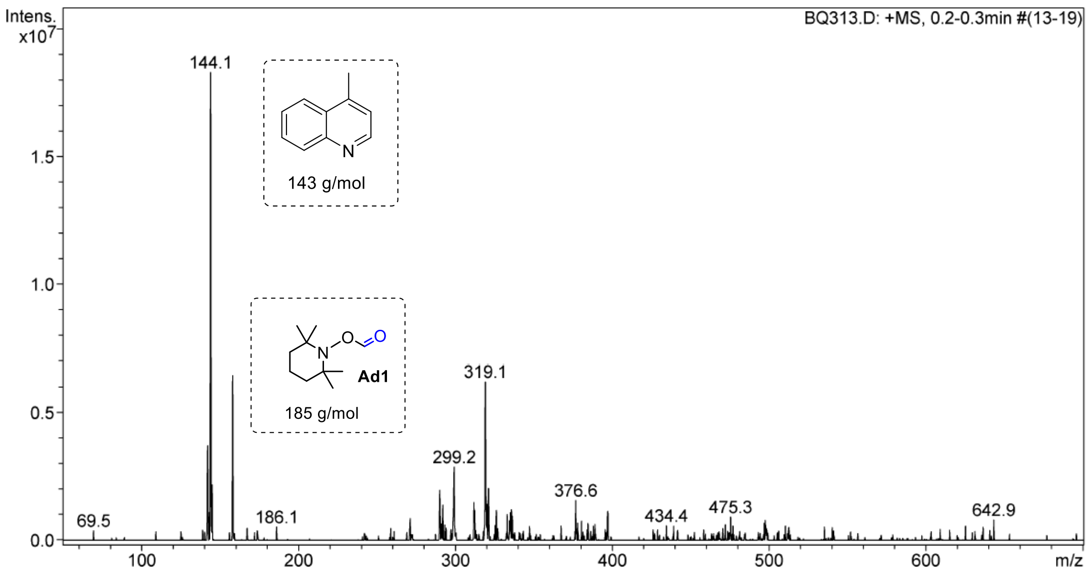

Figure S6: MS obtained for the reaction performed under GPA conditions and 2 equivalents of TEMPO.

Addition of 1,1-Diphenylethylene

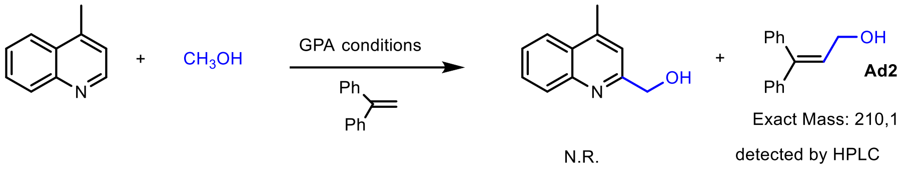

In a 10 mL vial equipped with a stirring bar was added lepidine (0.30 mmol), LiCl (38 mg, 0.90 mmol, 3 equiv., in 1.2 mL of H_2O), 9-(2-chlorophenyl)acridine (**A1**, 4.5 mg, 0.015 mmol, 5 mol%) and 1,1- diphenylethylene (DPE, 104 µL, 0.6 mmol, 2 equiv.), followed by MeOH (4.2 mL) and HCl (1 M, 0.6 mL, 2 equiv.). The electrodes Gf(+)/Ni (-) were then inserted, and the reaction was stirred under galvanostatic conditions (2 mA) for 24 h at room temperature. Once the time elapsed, the reaction mixture was analyzed by HPLC (ES+). While the hydroxymethyl derivative was not observed, an adduct of DPE with the hydroxymethyl radical (**Ad2** in Figure S7) was detected.

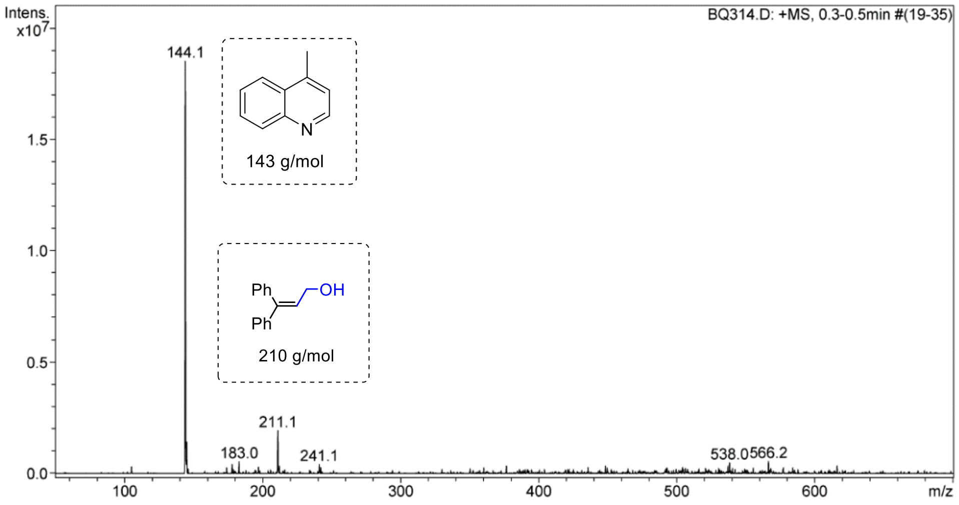

Figure S7: MS obtained for the reaction performed under GPA conditions and 2 equiv. of DPE.

Addition of $CuCl_2$

In a 10 mL vial equipped with a stirring bar was added lepidine (0.30 mmol), LiCl (38 mg, 0.90 mmol, 3 equiv., in 1.2 mL of H_2O), 9-(2-chlorophenyl)acridine (**A1**, 4.5 mg, 0.015 mmol, 5 mol%) and $CuCl_2$ (120 mg, 0.9 mmol, 3 equiv.), followed by MeOH (4.2 mL) and HCl (1 M, 0.6 mL, 2 equiv). The electrodes Gf (+)/Ni (-) were then inserted, and the reaction was stirred under galvanostatic conditions (2 mA) for 24 h at room temperature. Once the time elapsed, the reaction mixture was analyzed by GC/MS, and no reaction was observed. This result suggests that electron transfers are key steps of this reaction.

Trapping Cl radical with 1,1-Diphenylethylene

In a 10 mL vial equipped with a stirring bar was added 1,1-diphenylethylene (53 µL, 0.30 mmol), LiCl (38 mg, 0.90 mmol, 3 equiv., in 1.2 mL of H_2O), 9-(2-chlorophenyl)acridine (**A1**, 4.5 mg, 0.015 mmol, 5 mol%) followed by MeCN (4.2 mL) and HCl (1 M, 0.6 mL, 2 equiv.). The electrodes Gf(+)/Ni (-) were then inserted, and the reaction was stirred under galvanostatic conditions (2 mA) for 24 h at room temperature. Once the time elapsed, the reaction mixture was analyzed by HPLC (ES+), observing the formation of 2-chloro-1,1-diphenylethan-1-ol by MS (Figure S8) and ^{1}H-NMR (Figure S9).

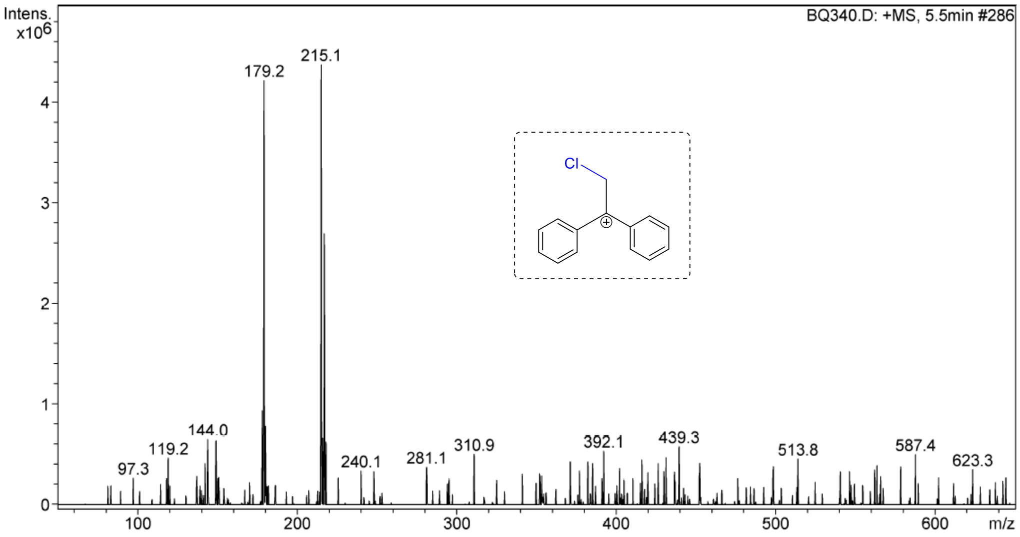

Figure S8: MS of the reaction performed without azaarene using 1 equiv. of DPE.

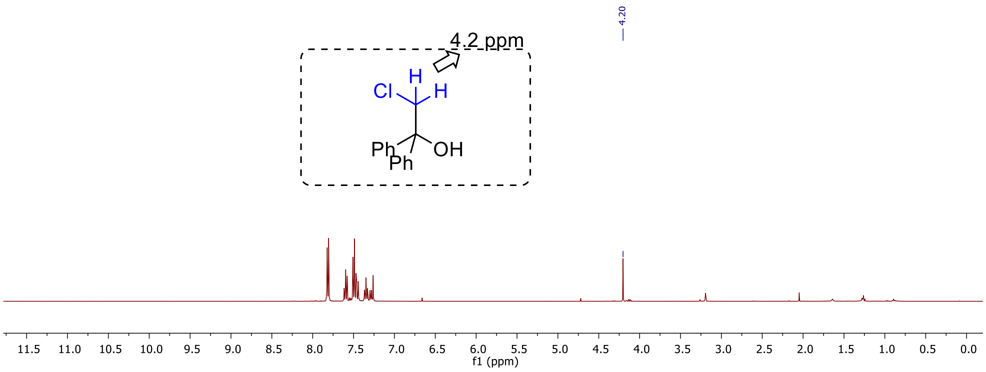

Figure S9: ^{1}H-NMR of the reaction performed without azaarene using 1 equiv. of DPE.

Cyclic Voltammetry (CV) measurements

CV measurements were performed using an EmStatblue+ potentiostat at a scan rate of 100 mV/s. A glassy carbon electrode of 3 mm diameter was used as a working electrode (polished with alumina 0.05 µm slurry), a platinum wire as a counter electrode, and a Ag/AgCl (aq., 0.1 M KCl) as a reference electrode. The measurements were performed in a 3 mL flask. Arrows indicate the sweep direction of the scans. $E_{p/2}$ were calculated as $E_{p/2} = (E_p - E_{onset})/2$. All measured potentials (*vs*. Ag/AgCl) were referred to the SCE by adding +0.0466 V.

As observed in Figure S10, the oxidation potential of anion chloride is dependent on the media, being shifted to higher potentials in an acidic/aqueous media, such as the one used in the reactions described in this work.

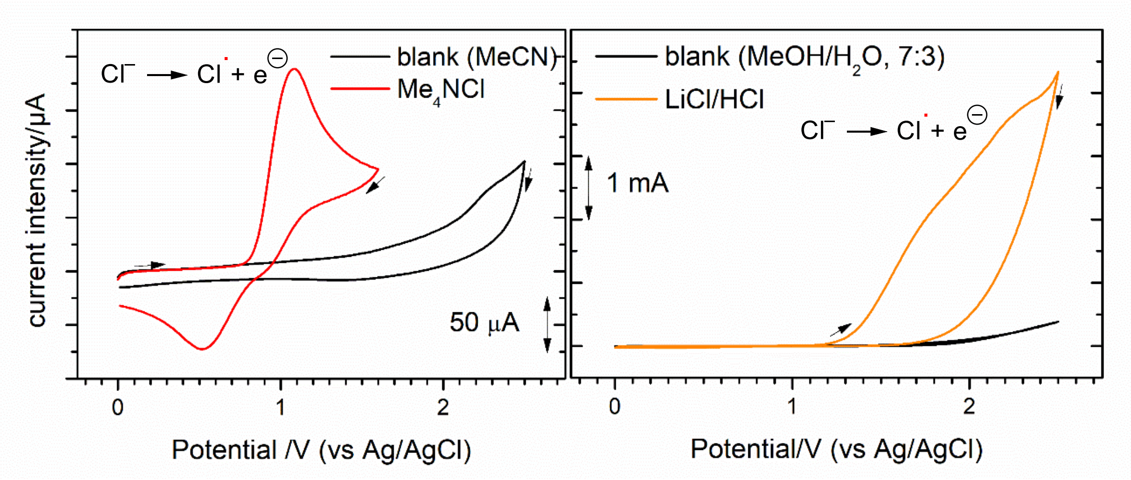

Figure S10: **Left**: CV of blank solution (0.1 M Bu_4NPF_6 in MeCN, black line) and after addition of Me_4NCl (0.3 M, red line). **Right**: CV of blank solution (0.05 M Bu_4NPF_6 in MeCN:H_2O (7:3), black line), and after addition of LiCl (16 mg) and HCl (0.3 mL at 37%) $[Cl^-]_f$ = 1.2 M, $[H^+]_f$ = 1.1 M (orange line). CVs plotted using the IUPAC convention.

According to these CVs, the oxidation potential of chloride anion to the corresponding radical in MeCN is $E_{p/2}$ = +0.95 V (*vs*. Ag/AgCl (0.1 M KCl) [$E_{p/2}$ = +1.00 V *vs*. SCE], and in acidic MeOH/H_2O media is $E_{p/2}$ > +1.60 V (*vs*. Ag/AgCl (0.1 M KCl) [$E_{p/2}$ > +1.65 V *vs*. SCE].

The observed potential of our reaction model ranged from +1.2 to +1.5 V (*vs*. Ag/AgCl (0.1 M KCl) [$E_{p/2}$ = +1.25 to +1.55 V *vs*. SCE] over 24 h, which is lower than the one required for the oxidation of chloride anion under the same media. This indicates that the electrochemical oxidation of chloride anions is unlikely under the reaction conditions used.

On the other hand, the reduction potential of the protonated catalyst **A1** was also measured under the reaction conditions (MeCN:H_2O (7:3)) using TFA or HCl (Figure S11, red lines). These CVs were compared to the CVs of both acids (green lines).

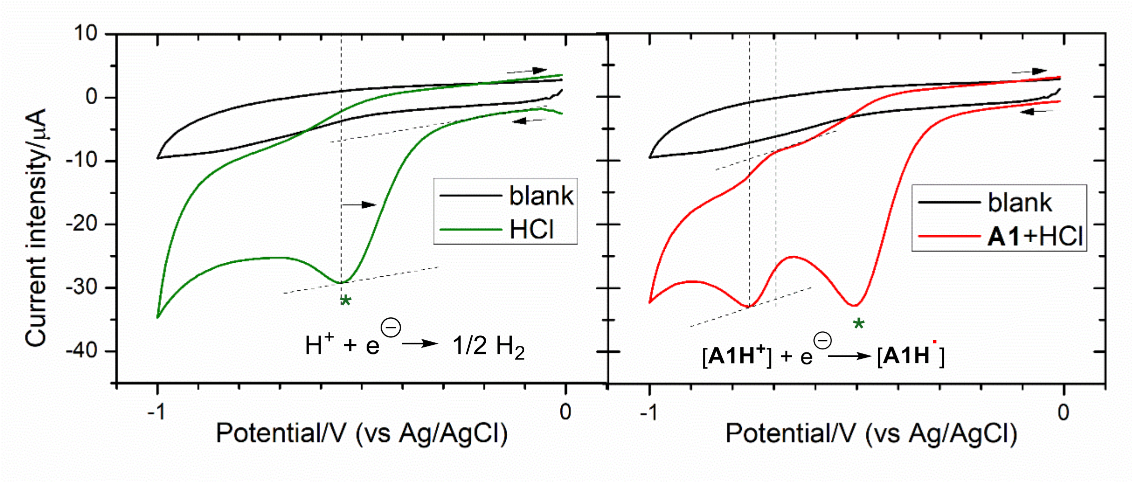

Figure S11: CVs of blank solution (0.1 M LiCl in MeOH:H_2O (7:3), black line) and after addition of HCl (green line), or after addition of **A1** ([**A1**] = $5\cdot10^{-3}$ M + HCl (0.3 mL at 37%)) (red line). CVs plotted using the IUPAC convention.

According to these results, it seems plausible to say that the current of the first peak is related to the reduction of H^+ due to the acid excesses ($E_{p/2}$ = –0.43 V (*vs*. Ag/AgCl (0.1 M KCl)) [$E_{p/2}$ = –0.38 V *vs*. SCE], and the second peak is the more relevant, resulting from the reduction of the [**A1H**]$^+$ catalyst ($E_{1/2}$ = –0.72 V (*vs*. Ag/AgCl (0.1 M KCl)) [$E_{1/2}$ = –0.67 V *vs*. SCE]. This value is similar to the one reported for this redox pair in MeCN (–0.56 V vs. SCE).[7]

CHARACTERIZATION OF PRODUCTS

(2-Phenyl-4-yl)methanol (1):

Following GPA with 2-phenylquinoline (62 mg, 0.30 mmol) in 24 h. The product was obtained as a white oil (55 mg, 0.23 mmol, 78%) after FC using a gradient from 0% to 30% of EtOAc in *n*-hexane as the eluent. The spectroscopy data matched with previously reported in the literature.[8]

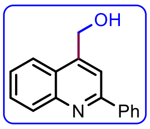

TLC: Rf = 0.48 (7:3 hexane/EtOAc, UV).

GC (Ti= 80 ºC): Rt 9.5 min.

MS: *m/z* (%) 235 (M^+ 100), 234 (68), 206 (64), 204 (41), 205 (32).

^{1}H NMR (400 MHz, DMSO-d$_6$): δ 8.29 – 8.22 (m, 2H), 8.17 (d, J = 1.2 Hz, 1H), 8.10 (dd, J = 8.6, 1.3 Hz, 1H), 8.07 (dd, J = 8.4, 1.4 Hz, 1H), 7.77 (ddd, J = 8.4, 6.8, 1.4 Hz, 1H), 7.62 – 7.48 (m, 4H), 5.75 – 5.61 (m, 1H), 5.11 (d, J = 4.2 Hz, 2H).

^{13}C NMR (101 MHz, DMSO-d$_6$): δ 156.3, 149.2, 147.8, 139.3, 130.1, 130.0, 129.3, 127.5, 126.7, 125.1, 123.9, 115.7, 60.4.

(2-Methyl-4-yl)methanol (2):

Following GPA at 4 mA with quinaldine (40 µL, 0.30 mmol) in 24 h. The product was obtained as a yellow solid (46 mg, 0.26 mmol, 89%) after FC using a gradient from 0% to 50% of EtOAc in n-hexane as the eluent. The spectroscopy data matched with previously reported in the literature.[9]

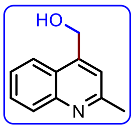

TLC: Rf = 0.17 (7:3 hexane/EtOAc, UV)

GC (Ti= 80 ºC): Rt 6.7 min.

MS: *m/z* (%) 144 (M^+ 100), 173 (94).

^{1}H NMR (400 MHz, CDCl$_3$): δ 8.00 (d, J = 8.5 Hz, 1H), 7.85 (dd, J = 8.3, 1.4 Hz, 1H), 7.70 – 7.59 (m, 1H), 7.46 (t, J = 7.6 Hz, 1H), 7.39 (s, 1H), 5.17 (s, 2H), 2.63 (s, 3H).

^{13}C NMR (101 MHz, CDCl$_3$): δ 159.0, 147.3, 146.5, 129.3, 128.8, 125.8, 124.1, 122.6, 119.1, 61.2, 25.1.

(2-Methyl-7-chloroquinoline -4-yl)methanol (3):

Following GPA with 2-methyl-7-chloroquinoline (53 mg, 0.30 mmol) in 24 h. The product was obtained as a white solid (40 mg, 0.19 mmol, 64%) after FC using a gradient from 0% to 50% of EtOAc in *n*-hexane as the eluent. The spectroscopy data matched with previously reported in the literature.[9]

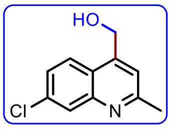

TLC: Rf = 0.20 (7:3 hexane/EtOAc, UV)

GC (Ti= 80 ºC): Rt 7.6 min.

MS: *m/z* (%) 207 (M^+ 100), 178 (92), 209 (32).

^{1}H NMR (400 MHz, CDCl$_3$/MeOD): δ 7.88 (d, J = 2.2 Hz, 1H), 7.74 (d, J = 8.9 Hz, 1H), 7.39 – 7.35 (m, 1H), 7.35 (d, J = 2.1 Hz, 1H), 5.01 (s, 2H), 2.61 (s, 3H).

^{13}C NMR (101 MHz, $CDCl_3$/MeOD): *δ* 160.3, 147.5, 147.4, 135.2, 127.2, 126.6, 124.1, 122.5, 119.2, 60.6, 24.7.

Methyl 4-(hydroxymethyl)-2-methylquinoline-6-carboxylate (4):

Following GPA with methyl-2-methylquinoline-6-carboxylate (60 mg, 0.30 mmol) in 24 h. After concentrating the rxm under vacuum, EtOAc (5 mL) was added and the white precipitated was filtered out and washed with EtOAc (2x 5 mL) to obtain the pure product (50 mg, 0.22 mmol, 72%). The spectroscopy data matched with previously reported in the literature. [10]

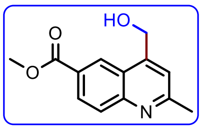

TLC: Rf = 0.25 (6:4 hexane/EtOAc, UV)

GC (Ti= 80 ºC): Rt 8.8 min.

MS: *m/z* (%) 200 (M^+ 100), 231 (93), 142 (92), 202 (42), 144 (36), 207 (32).

^{1}H NMR (400 MHz, DMSO-d_6): *δ* 8.64 (d, *J* = 1.9 Hz, 1H), 8.16 (d, *J* = 10.7 Hz, 1H), 8.00 (d, *J* = 8.8 Hz, 1H), 7.53 (s, 1H), 5.65 (t, *J* = 5.3 Hz, 1H), 5.03 (d, *J* = 6.5 Hz, 2H), 3.92 (s, 3H), 2.68 (s, 3H).

^{13}C NMR (101 MHz, DMSO-d_6): *δ* 166.5, 161.9, 149.6, 149.2, 129.7, 128.5, 126.6, 123.8, 120.5, 60.2, 52.8, 25.6.

(2-Methyl-6-bromoquinoline -4-yl)methanol (5):

Following GPA at 4 mA with of 2-methyl-6-bromoquinoline (66 mg, 0.30 mmol) in 24 h. The product was obtained as a white solid (45 mg, 0.18 mmol, 60%) after FC using a gradient from 0% to 50% of EtOAc in n-hexane as the eluent. The spectroscopy data matched with previously reported in the literature.[10]

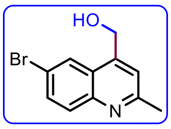

TLC: Rf = 0.27 (6:4 hexane/EtOAc, UV)

GC (Ti= 80 ºC): Rt 7.9 min.

MS: *m/z* (%) 251 (M^+ 100), 253 (94), 222 (68), 224 (65), 143 (52), 144 (33).

^{1}H NMR (400 MHz, $CDCl_3$/MeOD): *δ* 7.94 (d, *J* = 2.2 Hz, 1H), 7.75 (d, *J* = 9.0 Hz, 1H), 7.63 (dd, *J* = 9.0, 2.1 Hz, 1H), 7.39 (t, 1H), 4.96 (s, 2H), 2.59 (s, 3H).

^{13}C NMR (101 MHz, $CDCl_3$/MeOD): *δ* 159.5, 146.5, 145.5, 132.6, 129.9, 125.4, 125.2, 119.8, 119.7, 60.4, 24.6.

(2-Methyl-6-fluoroquinoline -4-yl)methanol (6):

Following GPA with 2-methyl-6-fluoroquinoline (48 mg, 0.30 mmol) in 24 h. The product was obtained as a white solid (37 mg, 0.19 mmol, 65%) after FC using a gradient from 0% to 60% of EtOAc in *n*-hexane as the eluent. The spectroscopy data matched with previously reported in the literature.[10]

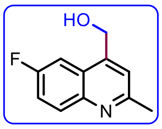

TLC: Rf = 0.14 (7:3 hexane/EtOAc, UV)

GC (Ti= 80 ºC): Rt 7.0 min.

MS: *m/z* (%) 162 (M^+ 100), 191 (90).

^{1}H NMR (400 MHz, $CDCl_3$): δ 8.03 (dd, J = 9.2, 5.5 Hz, 1H), 7.51 (dd, J = 9.8, 2.8 Hz, 1H), 7.47 – 7.39 (m, 2H), 5.11 (s, 2H), 2.71 (s, 3H).

^{13}C NMR (101 MHz, $CDCl_3$): δ 160.0 (d, J = 247.2 Hz), 158.3, 145.3 (d, J = 5.3 Hz), 144.8, 131.5 (d, J = 9.2 Hz), 124.8 (d, J = 9.3 Hz), 119.9, 119.2 (d, J = 25.4 Hz), 106.6 (d, J = 22.4 Hz), 61.7, 25.2.

^{19}F NMR (377 MHz, $CDCl_3$): δ -113.30.

(4-Methylquinolin-2-yl)methanol (7):

Following GPA at 4 mA with lepidine (43 µL, 0.30 mmol) in 24 h. The product was obtained as a white solid (49 mg, 0.28 mmol, 95%) after FC using a gradient from 0% to 50% of EtOAc in *n*-hexane as the eluent. The spectroscopy data matched with previously reported in the literature.[11]

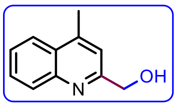

TLC: Rf = 0.2 (7:3 hexane/EtOAc, UV).

GC (Ti= 80 ºC): Rt 6.7 min.

MS: *m/z* (%) 172 (M^+ 100), 173 (82), 144 (73), 143 (43), 115 (33), 142 (32).

^{1}H NMR (400 MHz, $CDCl_3$): δ 8.08 (d, J = 8.4 Hz, 1H), 7.98 (d, J = 8.3 Hz, 1H), 7.71 (t, J = 7.6 Hz, 1H), 7.56 (t, J = 7.6 Hz, 1H), 7.13 (s, 1H), 4.87 (s, 2H), 2.70 (s, 3H).

^{13}C NMR (101 MHz, $CDCl_3$): δ 158.5, 146.4, 145.2, 129.5, 129.1, 127.6, 126.1, 123.8, 118.9, 63.9, 18.8.

(4-Methyl-6-bromoquinolin-2-yl)methanol (8):

Following GPA with 4-methyl-6-bromoquinoline (67 mg, 0.30 mmol) in 24 h. The product was obtained as a yellow oil (41 mg,0.16 mmol, 55%) after FC using a gradient from 0% to 50% of EtOAc in n-hexane as the eluent. The spectroscopy data matched with previously reported in the literature.[12]

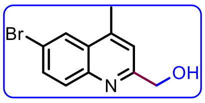

TLC: Rf = 0.23 (7:3 hexane/EtOAc, UV)

GC (Ti= 80 ºC): Rt 7.8 min.

MS: *m/z* (%) 252 (M^+ 100), 250 (95), 251 (87), 222 (81), 253 (79), 224 (66), 142 (59), 143 (48), 115 (41), 141 (35), 223 (33).

^{1}H NMR (400 MHz, $CDCl_3$): δ 8.09 (d, J = 2.2 Hz, 1H), 7.90 (d, J = 8.9 Hz, 1H), 7.75 (dd, J = 8.9, 2.2 Hz, 1H), 7.13 (s, 1H), 4.84 (s, 2H).

^{13}C NMR (101 MHz, $CDCl_3$): δ 159.2, 145.1, 144.2, 132.8, 130.8, 128.8, 126.3, 120.1, 119.7, 64.0, 18.7.

(4-Phenylquinolin-2-yl)methanol (9):

Following GPA with 4-phenylquinoline (62 mg, 0.30 mmol) in 24 h. The product was obtained as a yellow solid (45 mg,0.19 mmol, 65%) after FC using a gradient from 0% to 40% of EtOAc in *n*-hexane as the eluent.

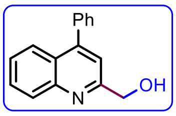

TLC: Rf = 0.25 (7:3 hexane/EtOAc, UV).

GC (Ti= 80 ºC): Rt 8.8 min.

MS: *m/z* (%) 234 (M^+ 100), 235 (74), 206 (56), 204 (42).

HRMS (EI-TOF): *m/z* calcd for $C_{16}H_{13}NO$ 235.0997, found 235.0969.

^{1}H NMR (400 MHz, $CDCl_3$): δ 8.14 (dt, J = 8.4, 1.0 Hz, 1H), 7.90 (dd, J = 8.5, 1.4 Hz, 1H), 7.76 – 7.67 (m, 1H), 7.56 – 7.40 (m, 6H), 7.25 (s, 1H), 4.96 (s, 2H).

^{13}C NMR (101 MHz, $CDCl_3$): δ 158.6, 149.3, 147.2, 137.9, 129.6, 129.4, 128.9, 128.6, 128.5, 126.4, 126.2, 125.9, 118.6, 64.2.

(4-Bromoquinolin-2-yl)methanol (10):

Following GPA with 4-bromoquinoline (62 mg, 0.30 mmol) in 24 h. The product was obtained as a red solid (36 mg), contaminated with (4-chloroquinolin-2-yl)methanol after FC using a gradient from 0% to 40% of EtOAc in n-hexane as the eluent. The ratio of the inseparable mixture was calculated according to ^{1}H-NMR as 29 mg (0.122 mmol, 41%) of the 4-bromo derivative and 7 mg (0.03 mmol, 12%) of the chloro derivative (53% overall yield). The spectroscopy data of the major product matched with previously reported in the literature. [11]

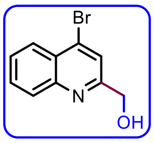

TLC: Rf = 0.17 (8:2 hexane/EtOAc, UV).

GC (Ti= 80 ºC): Rt 7.2 min.

MS: *m/z* (%) 238 (M^+ 100), 236 (96), 128 (79), 237 (76), 208 (74), 239 (73), 129 (60), 210 (55), 127 (43), 101 (42).

^{1}H NMR (400 MHz, $CDCl_3$): δ 8.17 (d, J = 9.3 Hz, 1H), 8.05 (d, J = 8.5 Hz, 1H), 7.76 (t, J = 8.4 Hz, 1H), 7.65 – 7.60 (m, 2H), 4.89 (s, 3H), 4.24 (s, 1H).

^{13}C NMR (101 MHz, $CDCl_3$): δ 159.2, 147.3, 134.7, 130.7, 129.0, 127.5, 126.8, 124.1, 122.3, 63.9.

(4-(Phenylethynyl)quinolin-2-yl)methanol (11):

Following GPA at 1.5 V cell voltage with 4-(phenylethynyl)quinoline (69 mg, 0.30 mmol) in 24 h, observing a current of 0.7 mA. The product was obtained as a green solid (40 mg, 0.154 mmol, 51%) after FC using a gradient from 0% to 60% of EtOAc in *n*-hexane as the eluent.

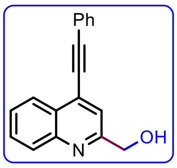

TLC: Rf = 0.25 (7:3 hexane/EtOAc, UV).

GC (Ti= 80 ºC): Rt 18.181 min

GC/MS: *m/z* (%) 258 (M^+ 100), 259 (80), 230 (40).

HRMS (EI-TOF): *m/z* calcd for $C_{18}H_{13}NO$ 259.0979, found 259.0971.

^{1}H NMR (400 MHz, $CDCl_3$): δ 8.36 (d, J = 8.3 Hz, 1H), 8.10 (d, J = 8.4 Hz, 1H), 7.77 (t, J = 7.7 Hz, 1H), 7.69 – 7.65 (m, 2H), 7.62 (d, J = 8.0 Hz, 1H), 7.49 (s, 1H), 7.45 – 7.40 (m, 3H), 4.93 (s, 2H).

^{13}C NMR (101 MHz, $CDCl_3$): δ 158.4, 146.6, 132.0, 130.5, 130.2, 129.4, 129.0, 128.6, 127.1, 126.9, 125.9, 122.1, 120.8, 98.6, 84.9, 64.0.

(3-Methylquinoline-2,4-diyl)dimethanol (12):

Following GPA with 3-methylquinoline (43 mg, 0.30 mmol) in 24 h. The product was obtained as a white solid (45 mg, 0.22 mmol, 74%) after FC using a gradient from 0% to 80% of EtOAc in *n*-hexane as the eluent.

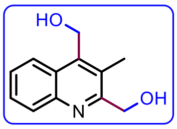

TLC: Rf = 0.35 (6:4 hexane/EtOAc, UV)

GC (Ti= 80 ºC): Rt 15.5 min.

GC/MS: *m/z* (%) 203 (M^+ 100), 167 (42), 281 (35), 231 (34).

HRMS (EI-TOF): *m/z* calcd for $C_{12}H_{13}NO_2$ 203.0946, found 203.0937.

^{1}H NMR (400 MHz, MeOD-d_4): δ 8.26 (d, J = 8.5 Hz, 1H), 8.04 (d, J = 10.0 Hz, 1H), 7.68 (t, J = 8.3 Hz, 1H), 7.63 – 7.56 (m, 1H), 5.12 (s, 2H), 4.88 (s, 2H), 2.54 (s, 3H).

^{13}C NMR (101 MHz, DMSO-d_6): δ 159.8, 145.3, 143.6, 129.1, 128.6, 128.1, 126.9, 126.5, 124.9, 64.3, 55.9, 13.5.

2,4-Bis(hydroxymethyl)quinoline-8-sulfonic acid (13):

Following GPA with 8-quinolinesulfonic acid (63 mg, 0.30 mmol) in 24 h. After filtration from the reaction mixture, the product was obtained as a white solid (40 mg, 0.15 mmol, 50%) and washed with EOtAc.

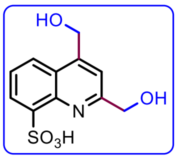

HRMS (ESI-TOF): *m/z* $(M+H)^+$ calcd for $C_{11}H_{13}NO_5S$ 270.0431, found 270.0451.

^{1}H NMR (400 MHz, DMSO-d_6): δ 8.37 (t, J = 9.6 Hz, 2H), 8.07 (s, 1H), 8.00 – 7.90 (m, 1H), 5.30 (s, 2H), 5.21 (s, 2H).

^{13}C NMR (101 MHz, DMSO-d_6): δ 161.2, 160.9, 137.3, 132.2, 131.5, 129.1, 126.6, 125.0, 115.96, 60.6, 60.4.

[2,2'-Biquinolin]-4-ylmethanol (14):

Following GPA with 2,2'-biquinoline (77 mg, 0.30 mmol) in 48 h. The product was obtained as a yellow solid (45 mg, 0.16 mmol, 53%) after FC using a gradient from 0% to 40% of EtOAc in *n*-hexane as the eluent.

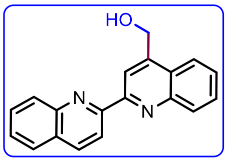

TLC: Rf = 0.4 (7:3 hexane/EtOAc, UV)

HRMS (EI-TOF): *m/z* calcd for $C_{19}H_{14}N_2O$ 286.1106, found 286.1105.

^{1}H NMR (400 MHz, DMSO-d$_6$): δ 8.93 (s, 1H), 8.81 (d, *J* = 8.6 Hz, 1H), 8.56 (d, *J* = 8.6 Hz, 1H), 8.20 (d, *J* = 7.8 Hz, 2H), 8.09 (dd, *J* = 14.7, 7.9 Hz, 2H), 7.84 (q, *J* = 7.8, 7.2 Hz, 2H), 7.68 (s, 2H), 5.76 (t, *J* = 5.6 Hz, 1H), 5.16 (s, 2H).

^{13}C NMR (101 MHz, DMSO-d$_6$): δ 156.0, 155.6, 149.4, 147.7, 147.5, 137.6, 130.7, 130.4, 130.1, 129.7, 128.6, 128.4, 127.7, 127.6, 126.1, 123.9, 119.3, 115.9, 60.4.

(1S*,2R*,5S*)-2-Isopropyl-5-methylcyclohexyl 2-(hydroxymethyl)quinoline-4-carboxylate (15):

Following GPA with ((1S*,2R*,5S*)-2-isopropyl-5-methylcyclohexyl quinoline-4-carboxylate) (94 mg, 0.30 mmol) in 24 h. The product was obtained as a red oil (45 mg, 0.13 mmol, 44%) after FC using a gradient from 0% to 50% of EtOAc in *n*-hexane as the eluent. Some starting material remained, and minor amounts of the corresponding formaldehyde were observed.

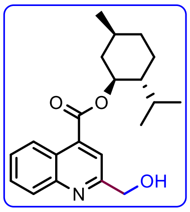

TLC: Rf = 0.4 (6:4 hexane/EtOAc, UV).

GC (Ti= 80 ºC): Rt 18.874 min

GC/MS: *m/z* (%) 204 (M^+ 80), 186 (60), 341 (40).

HRMS (EI-TOF): *m/z* calcd for $C_{21}H_{27}NO_3$ 341.1991, found 341.1973.

^{1}H NMR (400 MHz, $CDCl_3$): δ 8.70 (ddd, *J* = 8.5, 1.5, 0.6 Hz, 1H), 8.13 (ddd, *J* = 8.5, 1.5, 0.6 Hz, 1H), 7.77 (ddd, *J* = 8.5, 6.9, 1.5 Hz, 1H), 7.74 (s, 1H), 7.64 (ddd, *J* = 8.5, 6.9, 1.5 Hz, 1H), 5.10 (td, *J* = 10.9, 4.4 Hz, 1H), 4.98 (s, 2H), 2.22 (dtd, *J* = 11.9, 4.1, 1.8 Hz, 1H), 1.97 (pd, *J* = 7.0, 2.8 Hz, 1H), 1.81 – 1.72 (m, 2H), 1.70 – 1.51 (m, 3H), 1.29 – 1.09 (m, 2H), 0.97 (d, *J* = 6.5 Hz, 3H), 0.93 (d, *J* = 7.0 Hz, 3H), 0.85 (d, *J* = 6.9 Hz, 3H).

^{13}C NMR (101 MHz, $CDCl_3$): δ 165.6, 158.5, 147.7, 136.7, 130.0, 129.2, 127.7, 125.6, 124.5, 119.3, 76.1, 64.2, 47.1, 40.9, 34.2, 31.5, 26.4, 23.4, 22.0, 20.8, 16.2.

(2-Phenylpyridin-4-yl)methanol (16a):

Following GPA with 2-phenylpyridine (43 µL, 0.30 mmol) in 24 h. The products were obtained as yellow oils (C6 isomer: 4.5 mg, 0.02 mmol, 8%) (C4 isomer: 29 mg, 0.16 mmol, 52 %) after FC using a gradient from 0% to 40% of EtOAc in n-hexane as the eluent. The spectroscopy data matched with previously reported in the literature.[13]

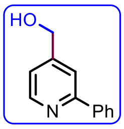

TLC: Rf = 0.26 (7:3 hexane/EtOAc, UV)

^{1}H NMR (400 MHz, $CDCl_3$): δ 8.60 (d, *J* = 6.6 Hz, 1H), 7.95 (d, *J* = 7.4 Hz, 2H), 7.69 (s, 1H), 7.44 (dt, *J* = 13.6, 7.0 Hz, 3H), 7.19 (d, *J* = 4.9 Hz, 1H), 4.76 (s, 2H).

^{13}C NMR (101 MHz, $CDCl_3$): δ 157.7, 150.8, 149.6, 139.2, 129.0, 128.7, 127.0, 119.6, 118.0, 63.6.

(2-Phenylpyridin-6-yl)methanol (16b):

A very minor amount of this isomer was obtained, which complicates to obtain clean samples for the NMR spectra.

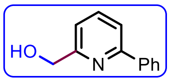

TLC: Rf = 0.53 (7:3 hexane/EtOAc, UV)

^{1}H NMR (400 MHz, $CDCl_3$): δ 8.02 (d, J = 8.2 Hz, 2H), 7.76 (t, 1H), 7.66 (d, 1H), 7.56 – 7.39 (m, 3H), 7.17 (d, 1H), 4.82 (s, 2H).

^{13}C NMR (101 MHz, $CDCl_3$): δ 138.7, 137.4, 129.2, 129.2, 128.7, 126.8, 119.0, 118.7, 63.8.

Ethyl 2-(hydroxymethyl)isonicotinate (17):

Following GPA with ethyl isonicotinate (46 µL, 0.30 mmol) in 24 h. The product was obtained as a white oil (19 mg, 0.10 mmol, 35%) after FC using a gradient from 0% to 50% of EtOAc in *n*-hexane as the eluent. The spectroscopy data matched with previously reported in the literature. [14] Unidentified byproducts are obtained.

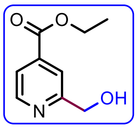

TLC: Rf = 0.28 (7:3 hexane/EtOAc, UV)

GC (Ti= 80 ºC): Rt 5.692 min.

GC/MS: *m/z* (%) 181 (M^+ 100), 152 (90), 181 (61), 136 (40), 124 (39), 153 (33).

^{1}H NMR (400 MHz, $CDCl_3$): δ 8.70 (d, J = 5.1 Hz, 1H), 7.83 (dd, J = 1.6, 0.9 Hz, 1H), 7.77 (ddt, J = 5.2, 1.6, 0.7 Hz, 1H), 4.84 (s, 2H), 4.42 (q, J = 7.1 Hz, 2H), 1.41 (t, J = 7.1 Hz, 3H).

^{13}C NMR (101 MHz, $CDCl_3$): δ 165.0, 160.2, 149.3, 138.4, 121.6, 119.8, 64.2, 61.9, 14.2.

2-(Hydroxymethyl)isonicotinonitrile (18):

Following GPA with 4-cyanopyridine (31 mg, 0.30 mmol) in 24 h. The product was obtained as a yellow oil (17 mg, 0.13 mmol, 42%) after FC using a gradient from 0% to 50% of EtOAc in *n*-hexane as the eluent. The spectroscopy data matched with previously reported in the literature.[10] According to ^{1}H-NMR, some 2,6-bis(hydroxymethyl)isonicotinonitrile was obtained as a byproduct, complicating the purification. However, this byproduct could not be isolated in pure form.

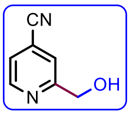

TLC: Rf = 0.4 (7:3 hexane/EtOAc, UV)

^{1}H NMR (400 MHz, $CDCl_3$): δ 8.75 (d, J = 4.9 Hz, 1H), 7.58 (s, 1H), 7.45 (d, J = 5.7 Hz, 1H), 4.85 (s, 2H), 3.24 (s, 1H).

^{13}C NMR (101 MHz, $CDCl_3$): δ 161.2, 149.7, 123.8, 122.2, 121.1, 116.4, 64.1.

(4-Chloropyridin-2-yl)methanol (19):

Following GPA with 4-chloropyridine (34 mg, 0.30 mmol) in 24 h. The product was obtained as a white solid (26 mg, 0.18 mmol, 60%) after FC using a gradient from 0% to 40% of EtOAc in *n*-hexane as the eluent. The spectroscopy data matched with previously reported in the literature.[13]

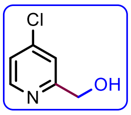

TLC: Rf = 0.35 (6:4 hexane/EtOAc, UV)

GC (Ti= 80 ºC): 4.031 min

GC/MS: *m/z* (%) 142 (M^+ 100), 143 (39), 114 (38), 144 (33).

^{1}H NMR (400 MHz, $CDCl_3$): δ 8.45 (d, J = 5.3 Hz, 1H), 7.35 – 7.29 (m, 1H), 7.22 (d, J = 7.3 Hz, 1H), 4.75 (s, 2H), 3.56 (s, 1H).

^{13}C NMR (101 MHz, $CDCl_3$): δ 161.0, 149.5, 144.8, 122.8, 120.8, 64.0.

(4-Chloropyridin-2-yl)methan-D$_2$-ol (20):

Following GPA with 4-chloropyridine (34 mg, 0.30 mmol) in 24 h. The product was obtained as a white solid (22 mg, 0.15 mmol, 51%) after FC using a gradient from 0% to 50% of EtOAc in *n*-hexane as the eluent.

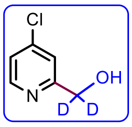

TLC: Rf = 0.34 (6:4 hexane/EtOAc, UV)

GC (Ti= 80 ºC): 4.010 min

GC/MS: *m/z* (%) 143 (M^+ 100), 145 (92), 115 (43), 144 (35).

HRMS (ESI-TOF): *m/z* $(M+H)^+$ calcd for $C_6H_4D_2ClNO$ 146.0336, found 146.0341.

^{1}H NMR (400 MHz, $CDCl_3$): δ 8.45 (d, J = 5.3 Hz, 1H), 7.32 (d, J = 1.7 Hz, 1H), 7.22 (dd, J = 5.4, 2.0 Hz, 1H).

^{13}C NMR (101 MHz, $CDCl_3$): δ 161.0, 149.5, 144.8, 122.8, 120.9.

6-(Hydroxymethyl)nicotinamide (21):

Following GPA at 1.5 V cell voltage with nicotinamide (37 mg, 0.30 mmol) in 48 h, observing a current intensity of 1 mA. The product was obtained as a yellow solid (25 mg, 0.16 mmol, 55%) after FC using a gradient from 0% to 40% of EtOAc: EtOH: NH_3 (49:49:2) in *n*-hexane as the eluent.

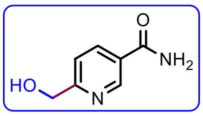

TLC: Rf = 0.35 (6:4 hexane/ EtOAc: EtOH:NH_3 ((49:49:2)), UV)

GC (Ti= 80 ºC): 13.020 min

GC/MS: *m/z* (%) 151 (M^+, 100), 152 (90).

HRMS (EI-TOF): *m/z* calcd for $C_7H_8N_2O_2$ 152.0586, found 152.0574.

^{1}H NMR (400 MHz, DMSO-d_6): δ 8.93 (dd, J = 2.3, 0.8 Hz, 1H), 8.21 (dd, J = 8.1, 2.3 Hz, 1H), 8.12 (s, 1H), 7.58 – 7.47 (m, 2H), 5.54 (t, J = 5.8 Hz, 1H), 4.60 (d, J = 5.8 Hz, 2H).

^{13}C NMR (101 MHz, DMSO-d_6): δ 166.9, 165.2, 148.2, 136.1, 128.3, 119.8, 64.5.

3-(cyclopropylmethoxy)-N-(3,5-dichloro-2-(hydroxymethyl)pyridin-4-yl)-4-(difluoromethoxy)benzamide (22):

Following GPA at 1.5 V cell voltage with Roflumilast (129 mg, 0.30 mmol) in 48 h, observing a current intensity of 1.9 mA. The product was obtained as a green solid (51 mg, 0.12 mmol, 39%) after FC using a gradient from 0% to 70% of EtOAc in *n*-hexane as the eluent. Some starting material remained unreacted.

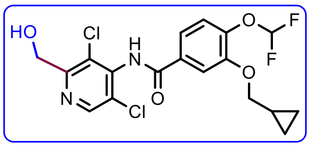

TLC: Rf = 0.30 (6:4 hexane/EtOAc, UV)

HRMS (EI-TOF): *m/z* calcd for $C_{18}H_{16}Cl_2F_2N_2O_4$ 432.0455, found 432.0473.

^{1}H NMR (400 MHz, $CDCl_3$): δ 8.54 (s, 1H), 7.91 (s, 1H), 7.58 (d, J = 2.1 Hz, 1H), 7.48 (dd, J = 8.3, 2.1 Hz, 1H), 7.29 – 7.26 (m, 1H), 6.74 (t, J = 74.8 Hz, 1H), 4.79 (s, 2H), 3.95 (d, J = 6.9 Hz, 2H), 1.34 – 1.28 (m, 1H), 0.70 – 0.63 (m, 2H), 0.37 (dt, J = 6.0, 4.7 Hz, 2H).

^{13}C NMR (101 MHz, $CDCl_3$): δ 163.8, 155.3, 150.9, 146.1, 140.2, 130.7, 127.8, 126.0, 122.3, 119.9, 115.6 (t, J = 261.6 Hz), 114.2, 74.2, 61.7, 10.0, 3.3.

^{19}F NMR (377 MHz, $CDCl_3$): δ -82.05.

Isoquinolin-1-ylmethanol (23):

Following GPB with isoquinoline (36 µL, 0.30 mmol) in 24 h. The product was obtained as a white solid (24 mg, 0.15 mmol, 50%) after FC using a gradient from 0% to 40% of EtOAc in *n*-hexane as the eluent. The spectroscopy data matched with previously reported in the literature.[9]

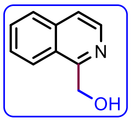

TLC: Rf = 0.30 (7:3 hexane/EtOAc, UV)

GC (Ti= 80 ºC): 6.171 min

GC/MS: *m/z* (%) 130 (M^+ 100), 158 (61), 159 (57), 128 (40), 129 (35).

^{1}H NMR (400 MHz, $CDCl_3$): δ 8.46 (d, 1H), 7.93 (d, J = 9.2 Hz, 1H), 7.87 (d, J = 8.2 Hz, 1H), 7.72 (t, J = 8.2 Hz, 1H), 7.66 – 7.58 (m, 2H), 5.24 (s, 2H).

^{13}C NMR (101 MHz, $CDCl_3$): δ 157.4, 140.4, 135.9, 130.4, 127.5, 127.3, 124.9, 123.1, 120.3, 61.4.

(6-Bromoisoquinolin-1-yl)methanol (24):

Following GPB with 6-bromoisoquinoline (62 mg, 0.30 mmol) in 24 h. The product was obtained as a red oil (34 mg, 0.144 mmol, 48%) after FC using a gradient from 0% to 40% of EtOAc in *n*-hexane as the eluent. The spectroscopy data matched with previously reported in the literature.[11] According to ^{1}H-NMR, some 6-bromoisoquinoline-1-carbaldehyde was obtained as a byproduct.

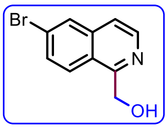

TLC: Rf = 0.35 (7:3 hexane/EtOAc, UV)

GC (Ti= 80 ºC): 6.677 min

GC/MS: *m/z* (%) 207 (M^+ 90), 209 (85), 237 (60), 235 (64).

^{1}**H NMR** (400 MHz, $CDCl_3$): δ 8.46 (d, J = 5.8 Hz, 1H), 8.03 (d, J = 1.9 Hz, 1H), 7.79 (d, J = 8.9 Hz, 1H), 7.69 (dd, J = 8.9, 1.9 Hz, 1H), 7.50 (d, J = 5.8 Hz, 1H), 5.20 (s, 2H), 4.95 (d, J = 51.3 Hz, 1H).

^{13}**C NMR** (101 MHz, $CDCl_3$): δ 157.7, 141.5, 137.0, 131.1, 129.5, 125.2, 124.9, 123.4, 119.2, 61.4.

(7-Bromoisoquinolin-1-yl)methanol (25):

Following GPB with 7-bromoisoquinoline (62 mg, 0.30 mmol) in 24 h. The product was obtained as an orange oil (34 mg, 0.144 mmol, 48%) after FC using a gradient from 0% to 50% of EtOAc in *n*-hexane as the eluent. According to ^{1}H-NMR, some 7-bromoisoquinoline-1-carbaldehyde was obtained as a byproduct.

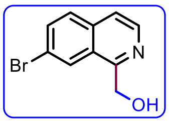

TLC: Rf = 0.25 (7:3 hexane/EtOAc, UV)

GC (Ti= 80 ºC): 6.801 min

HRMS (EI-TOF): *m/z* calcd for $C_{10}H_8BrNO$ 236.9789, found 236.9755.

GC/MS: *m/z* (%) 207 (M^+ 100), 209 (90), 235 (60).

^{1}**H NMR** (400 MHz, $CDCl_3$): δ 8.49 (d, J = 5.8 Hz, 1H), 8.08 (s, 1H), 7.80 (d, J = 8.8 Hz, 1H), 7.75 (d, J = 8.8 Hz, 1H), 7.58 (d, J = 5.6 Hz, 1H), 5.19 (s, 2H).

^{13}**C NMR** (101 MHz, $CDCl_3$): δ 156.6, 140.9, 134.3, 134.0, 129.1, 125.9, 125.7, 121.4, 120.0, 61.4.

(4-Bromoisoquinolin-1-yl)methanol (26):

Following GPB with 4-bromoisoquinoline (62 mg, 0.30 mmol) in 24 h. The product was obtained as a red oil (22 mg, 0.093 mmol, 31%) after FC using a gradient from 0% to 50% of EtOAc in *n*-hexane as the eluent. The spectroscopy data matched with previously reported in the literature.[10] According to ^{1}H-NMR, some 4-bromoisoquinoline-1-carbaldehyde and isoquinolin-1-ylmethanol were formed as byproducts, complicating the purification.

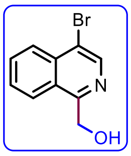

TLC: Rf = 0.20 (7:3 hexane/EtOAc, UV)

GC (Ti= 80 ºC): 7.345 min

GC/MS: *m/z* (%) 238 (M^+ 100), 236 (90), 251 (51).

^{1}**H NMR** (400 MHz, $CDCl_3$): δ 8.64 (s, 1H), 8.21 (d, J = 8.5 Hz, 1H), 7.92 (dd, J = 8.4, 1.1 Hz, 1H), 7.83 (ddd, J = 8.3, 7.0, 1.2 Hz, 1H), 7.71 – 7.65 (m, 1H), 5.19 (d, J = 2.6 Hz, 2H), 4.74 – 4.64 (m, 1H).

^{13}**C NMR** (101 MHz, $CDCl_3$): δ 157.0, 142.1, 134.5, 131.7, 128.5, 126.8, 126.0, 123.5, 118.9, 61.4.

1-(4-((1-(hydroxymethyl)isoquinolin-4-yl)sulfonyl)-1,4-diazepan-1-yl)ethan-1-one (27):
Following GPB with *N*-acetyl fasudil (108 mg, 0.30 mmol) in 24 h. The product was obtained as a green oil (40 mg, 0.110 mmol, 37%) after FC using a gradient from 0% to 10% of MeOH in dichloromethane (DCM) as the eluent. The spectroscopy data matched with previously reported in the literature.[15] The corresponding formaldehyde was detected as a byproduct after the reaction. Furthermore, after some days in the freezer, the product was completely oxidized to the corresponding formaldehyde.

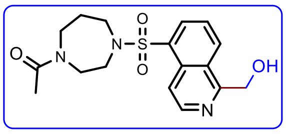

TLC: Rf = 0.4 (10:1 DCM/MeOH, UV)

^{1}H NMR (400 MHz, $CDCl_3$) for a 2:1 mixture of rotamers: δ 8.60 (s, 1H), 8.37 (s, 2H), 8.19 (d, J = 12.5 Hz, 1H), 7.69 (d, J = 8.4 Hz, 1H), 5.27 (s, 2H), 4.84 (s, 1H), 3.73 – 3.70 (m, 1H), 3.67 – 3.57 (m, 3H), 3.51 – 3.35 (m, 4H), 2.06 (s, 1H), 2.04 (s, 2H), 2.01 – 1.95 (m, 2H).

^{13}C NMR (101 MHz, $CDCl_3$) for a 2:1 mixture of rotamers: δ 170.2, 170.0, 158.6, 158.5, 142.64, 142.60, 135.11, 135.06, 132.9, 132.8, 131.8, 128.96, 128.93, 126.2, 125.9, 125.8, 117.0, 116.9, 61.8, 50.8, 50.0, 49.1, 48.4, 47.8, 47.6, 46.8, 44.5, 29.0, 27.6, 21.5, 21.0.

Phenanthridine-6-methyl (28):
Following GPA with phenantridine (54 mg, 0.30 mmol) in 24 h. The product was obtained as a white solid (40 mg, 0.21 mmol, 70%) after FC using a gradient from 0% to 10% of EtOAc in *n*-hexane as the eluent. The spectroscopy data matched with previously reported in the literature.[16]

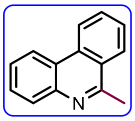

TLC: Rf = 0.4 (9:1 hexane/EtOAc, UV).

GC (Ti= 80 ºC): 7.580 min.

GC/MS: *m/z* (%) 193 (M^+ 100).

^{1}H NMR (300 MHz, $CDCl_3$): δ 8.59 (d, J = 8.7 Hz, 1H), 8.51 (d, J = 8.1 Hz, 1H), 8.19 (d, J = 7.7 Hz, 1H), 8.10 (d, J = 8.1 Hz, 1H), 7.86 – 7.76 (m, 1H), 7.72 (d, J = 8.4 Hz, 1H), 7.67 (s, 1H), 7.66 – 7.56 (m, 1H), 3.03 (s, 3H).

^{13}C NMR (101 MHz, $CDCl_3$): δ 158.8, 143.6, 132.5, 130.4, 129.3, 128.6, 127.2, 126.5, 126.3, 125.8, 123.7, 122.3, 121.9, 23.4.

Phenanthridine-6-carbaldehyde (29):
Following GPB with phenantridine (54 mg, 0.30 mmol) in 24 h, but fixing the voltage at 1.5 V. The product was obtained as a yellow solid (40 mg, 0.19 mmol, 64%) after FC using a gradient from 0% to 10% of EtOAc in *n*-hexane as the eluent. The spectroscopy data matched with previously reported in the literature.[17] When the same reaction was conducted under Ar atmosphere, 55% of the product was obtained (34 mg, 0.16 mmol).

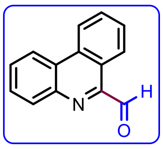

TLC: Rf = 0.5 (9:1 hexane/EtOAc, UV)

GC (Ti= 80 ºC): 7.967 min

GC/MS: *m/z* (%) 179 (M^+ 100), 207 (55), 178 (38), 142 (32).

^{1}H NMR (400 MHz, $CDCl_3$): δ 10.41 (s, 1H), 9.41 (d, J = 9.0 Hz, 1H), 8.66 (d, J = 8.3 Hz, 1H), 8.61 (d, J = 9.6 Hz, 1H), 8.34 (s, 1H), 7.90 (t, J = 7.7 Hz, 1H), 7.79 (d, J = 18.4 Hz, 3H).

^{13}C NMR (101 MHz, $CDCl_3$): δ 195.7, 150.2, 143.3, 133.4, 131.3, 131.2, 129.9, 129.2, 128.7, 126.9, 125.6, 123.5, 122.2, 121.9.

Phenanthridine-6-carbaldehyde-D (30):

Following GPB with phenantridine (27 mg, 0.15 mmol) in 24 h, but fixing the voltage at 1.5 V. The product was obtained as a white solid (25 mg, 0.12 mmol, 80%) after FC using a gradient from 0% to 10% of EtOAc in *n*-hexane as the eluent.

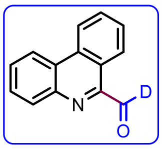

TLC: Rf = 0.50 (9:1 hexane/EtOAc, UV)

GC (Ti= 80 ºC): 7.947 min

GC/MS: *m/z* (%) 180 (M^+ 100), 208 (66),142 (48), 178 (37), 179 (35), 151 (31).

HRMS (EI-TOF): *m/z* calcd for $C_{14}H_8DNO$ 208.0747, found 208.0749.

^{1}H NMR (400 MHz, $CDCl_3$): δ 9.42 (d, J = 8.2 Hz, 1H), 8.75 – 8.51 (m, 2H), 8.40 – 8.24 (m, 1H), 7.96 – 7.68 (m, 4H).

^{13}C NMR (101 MHz, $CDCl_3$): δ 195.3, 150.2, 143.3, 133.4, 131.2, 131.1, 129.9, 129.2, 128.7, 126.9, 125.6, 123.5, 122.2, 121.9.

4-Methyl-2-phenylquinoline (31):

In a two-dram vial equipped with a stirring bar, was added 2-phenylquinoline (66 mg, 0.30 mmol), pyridine *N*-oxide (PyNO, 8.4 mg, 0.09 mmol, 30 mol%), and 9-(2-Chlorophenyl)acridine (**A1**, 4.4 mg, 0.015 mmol, 5 mol%), followed by a mixture of $MeOH/H_2O$ (7:3, 3 mL). Then, TFA (45 µL, 0.60 mmol, 2 equiv.) was added, and the mixture was stirred and irradiated with blue LEDs at room temperature for 24h. The product was obtained as a white solid (53 mg, 0.24 mmol, 80%) after FC using a gradient from 0% to 10% of EtOAc in *n*-hexane as the eluent. The spectroscopy data matched with previously reported in the literature.[18]

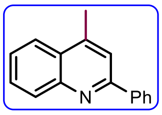

TLC: Rf = 0.42 (9:1 Hexane/EtOAc, UV).

GC (Ti= 80 ºC): 8.512 min.

GC/MS: *m/z* (%) 204 (M^+ 100), 219 (88), 218 (83), 217 (48), 220 (36).

^{1}H NMR (300 MHz, $CDCl_3$): δ 8.22 – 8.13 (m, 3H), 8.00 (d, J = 9.3 Hz, 1H), 7.77 – 7.69 (m, 2H), 7.58 – 7.49 (m, 3H), 7.47 (d, J = 7.3 Hz, 1H), 2.77 (s, 3H).

^{13}C NMR (101 MHz, $CDCl_3$): δ 157.1, 148.1, 144.8, 139.8, 130.3, 129.3, 129.2, 128.8, 127.5, 126.0, 123.6, 119.8, 19.0.

2-Phenylquinoline-4-carbaldehyde (32):

To an undivided three-necked flask were added (2-phenyl-4-yl)methanol (70 mg, 0.30 mmol), $^{n}Bu_4NBF_4$ (164 mg, 0.5 mmol, 0.05 M), TFA (27 µL, 0.36 mmol, 1.2 equiv.) and CH_3CN (10 mL). The flask was equipped with graphite felt as anode and platinum plate electrode as cathode. The reaction mixture was stirred and electrolyzed at a constant current (5 mA) under air at 60 °C (oil bath) for 5 h. The product was obtained as a white solid (28 mg, 0.12 mmol, 40%) after FC using a gradient from 0% to 20% of EtOAc in *n*-hexane as the eluent. The spectroscopy data matched with previously reported in the literature. [19]

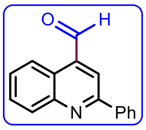

TLC: Rf = 0.4 (9:1 Hexane/EtOAc, UV).

GC (Ti= 80 ºC): 8.850 min.

GC/MS: *m/z* (%) 204 (M^+ 100), 233 (97), 205 (42).

^{1}H NMR (300 MHz, $CDCl_3$): δ 10.60 (s, 1H), 9.00 (dd, J = 8.5, 1.5 Hz, 1H), 8.30 – 8.19 (m, 4H), 7.83 (ddd, J = 8.4, 6.9, 1.5 Hz, 1H), 7.71 (ddd, J = 8.3, 6.9, 1.4 Hz, 1H), 7.61 – 7.49 (m, 3H).

^{13}C NMR (101 MHz, $CDCl_3$): δ 192.9, 157.4, 149.4, 138.5, 137.7, 130.3, 130.3, 130.0, 129.0, 128.9, 127.4, 124.1, 124.0, 122.9.

UNSUCCESSFUL SUBSTRATES

Table S3: Unsuccessful starting materials.

Substrate	Method: result	Substrate	Method: result
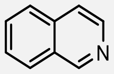	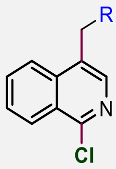 **GPA:** R= OH (26%) R= H (19%)	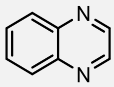	**GPA**: Decompose
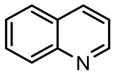	**GPA:** Many products	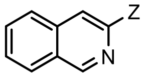 Z= CN or CO_2Me	**GPB**: Decompose
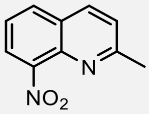	**GPA:** No reaction	Camptothecin	**GPA or GPB:** Low conversion
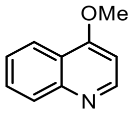	**GPA or GPB:** No reaction	Chinchonine	**GPA or GPB:** Low conversion
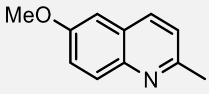	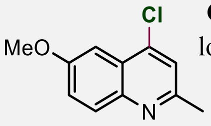 **GPA:** low yield	Quinine	**GPA or GPB:** Low conversion
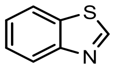	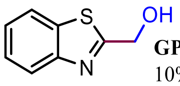 **GPA:** 10%	Nicotine	**GPA or GPB:** Low conversion
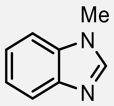	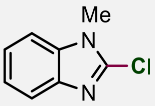 **GPA:** 16%	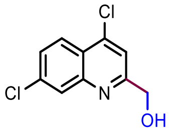	**GPA** Low reproducibility. 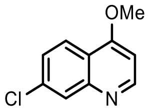 Byproduct

NMR SPECTRA OF SYNTHESIZED COMPOUNDS

^{1}H NMR (400 MHz, DMSO-d$_6$) **(1)**

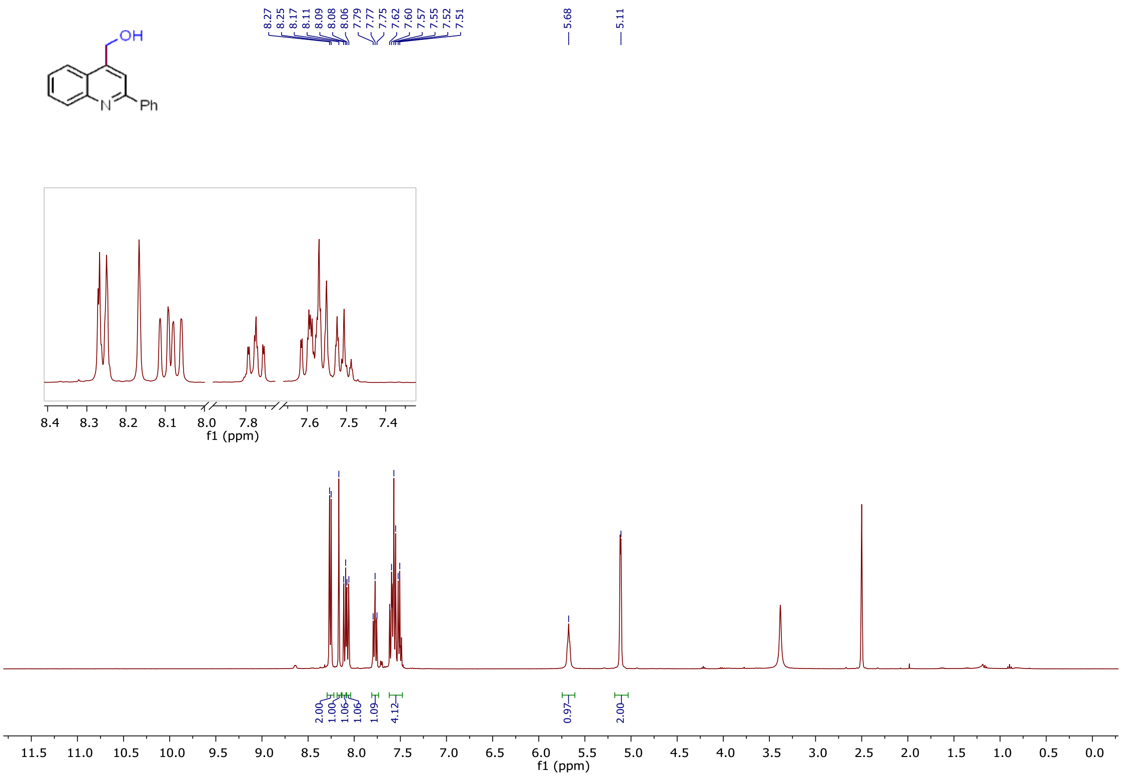

^{13}C NMR (101 MHz, DMSO-d$_6$) **(1)**

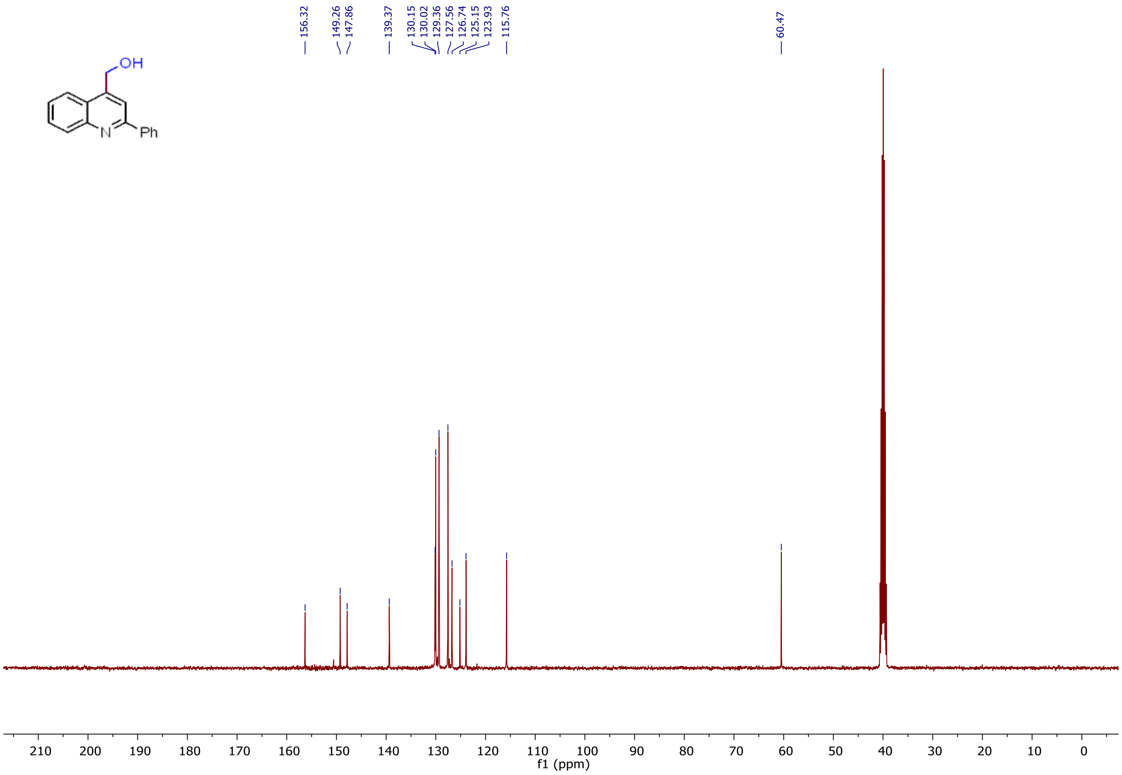

^{1}H NMR (400 MHz, $CDCl_3$) **(2)**

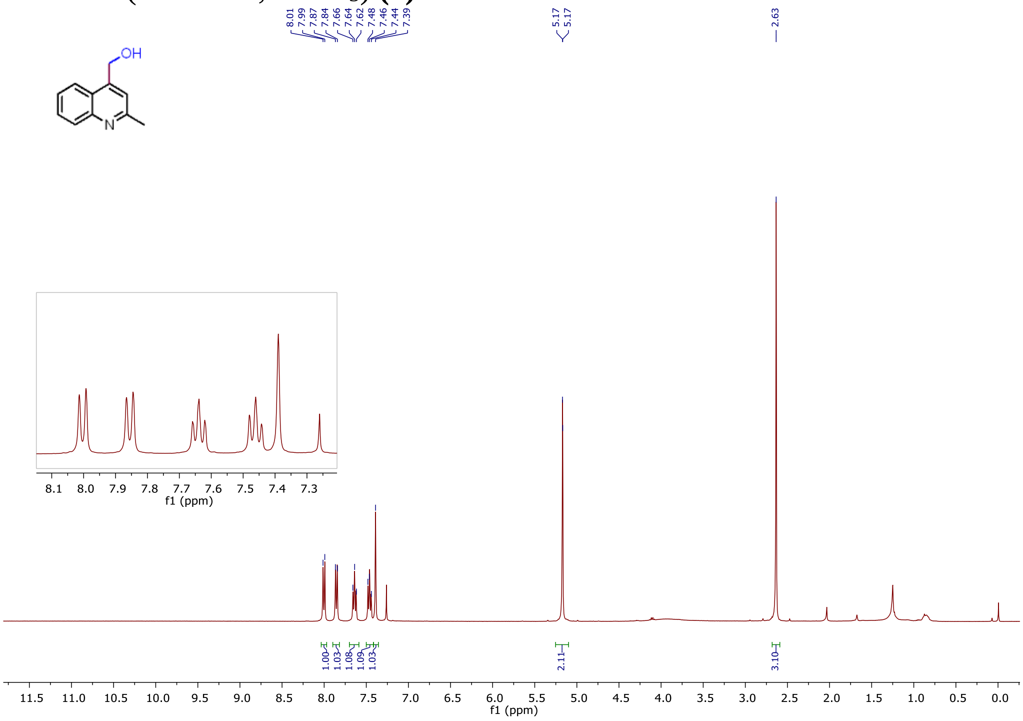

^{13}C NMR (101 MHz, $CDCl_3$) **(2)**

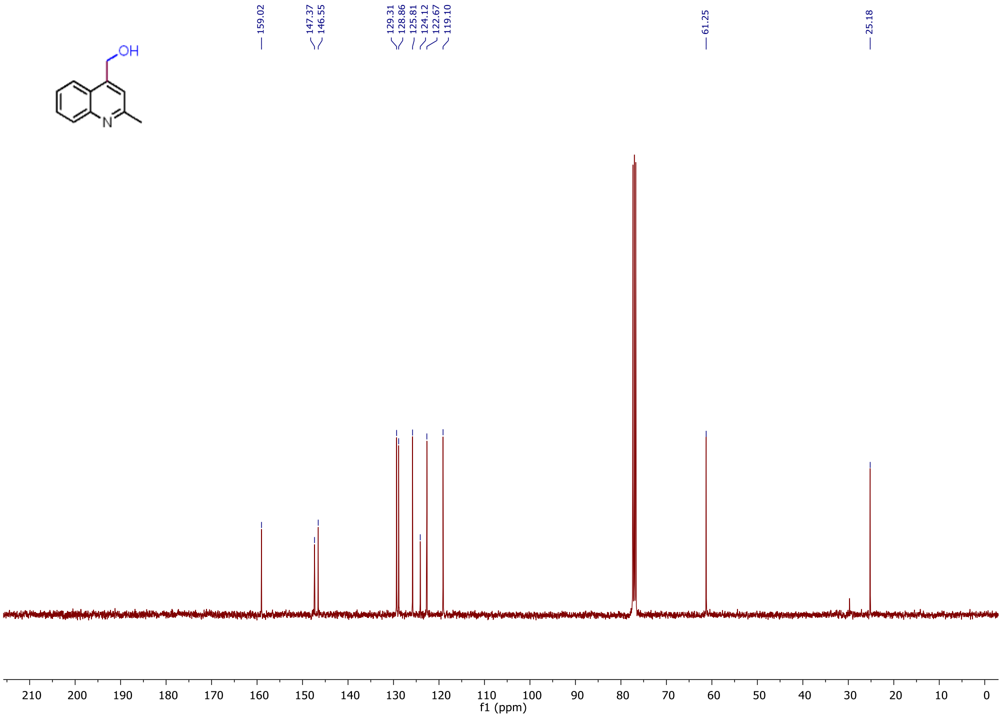

^{1}H NMR (400 MHz, $CDCl_3$/MeOD) **(3)**

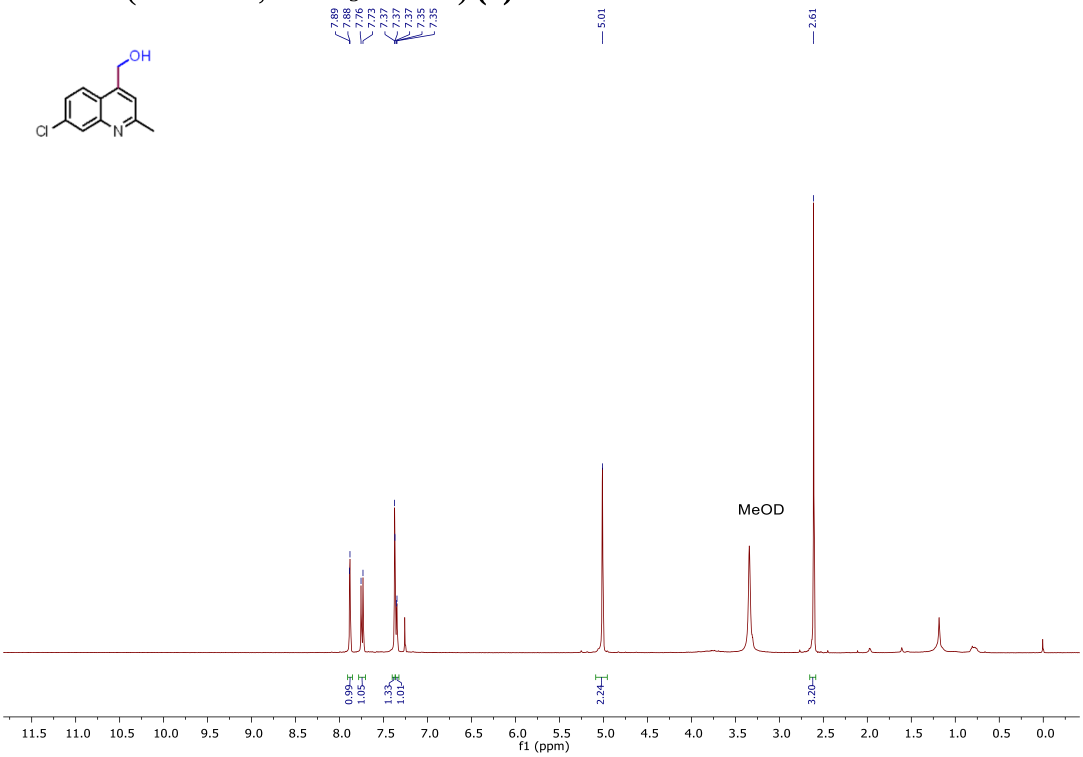

^{13}C NMR (101 MHz, $CDCl_3$/MeOD) **(3)**

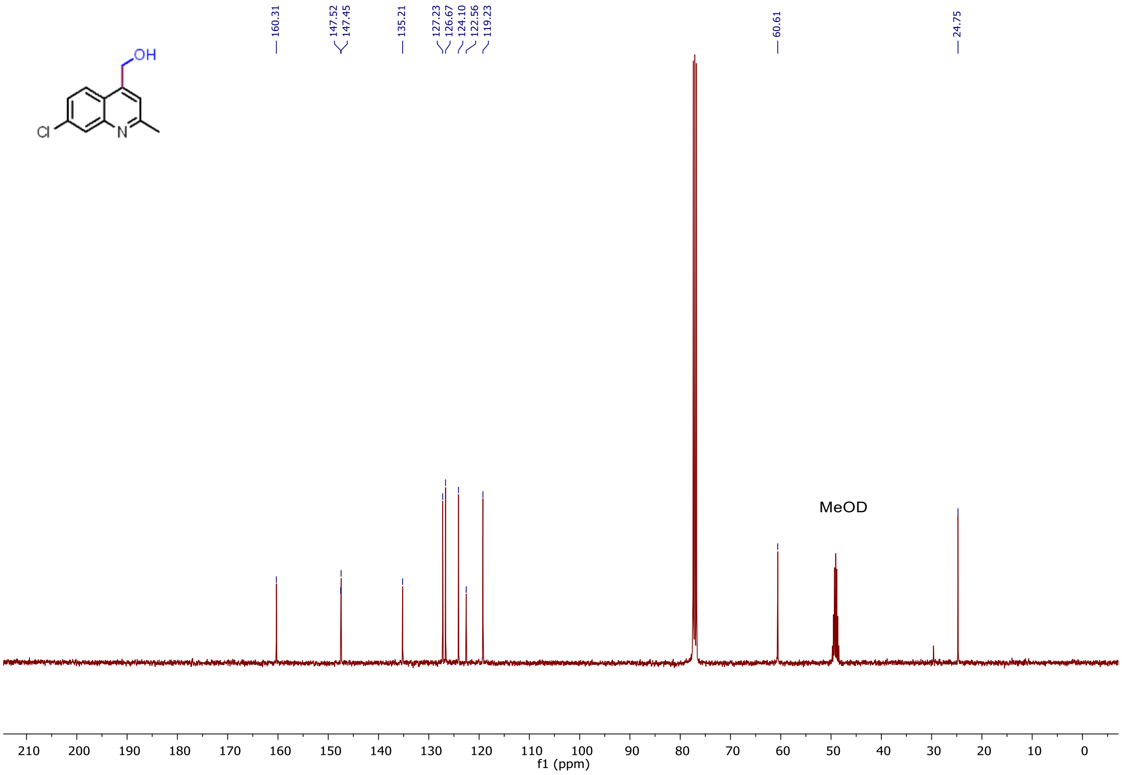

^{1}H NMR (400 MHz, DMSO-d6) **(4)**

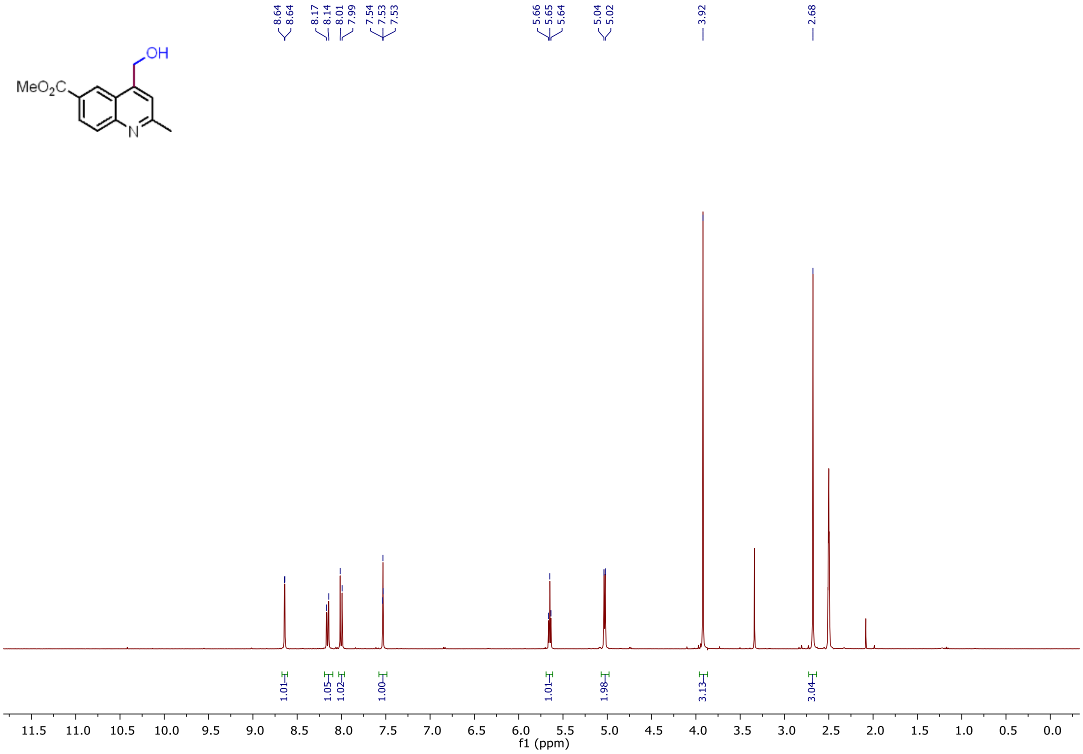

^{13}C NMR (101 MHz, DMSO-d6) **(4)**

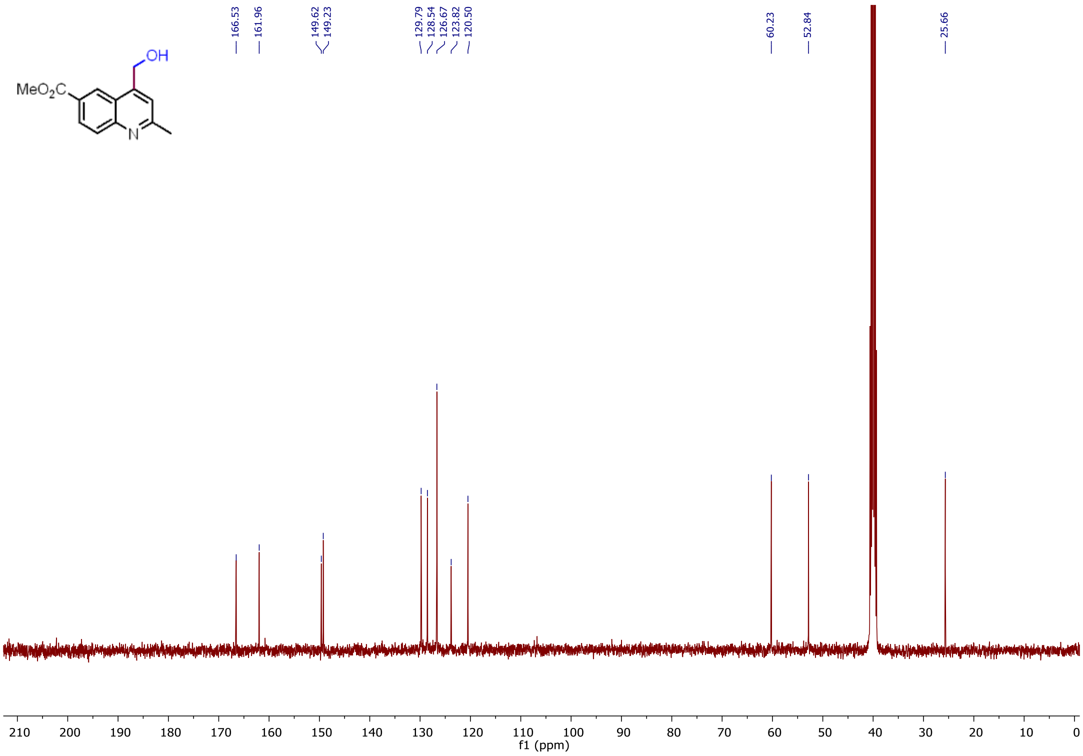

^{1}H NMR (400 MHz, $CDCl_3$/MeOD) **(5)**

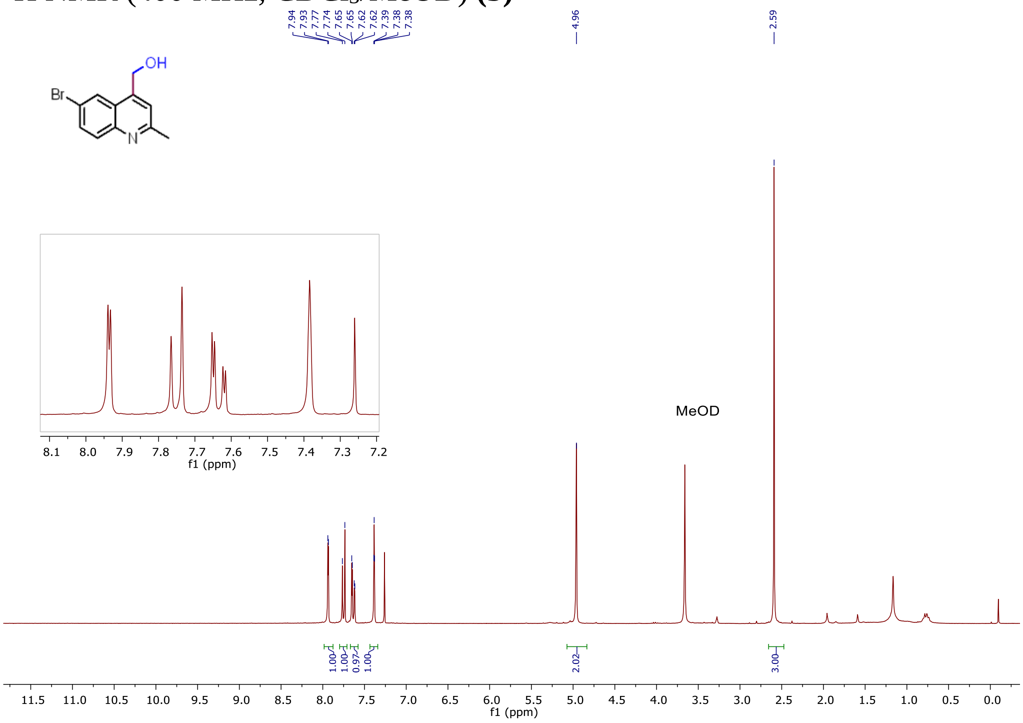

^{13}C NMR (101 MHz, $CDCl_3$/MeOD) **(5)**

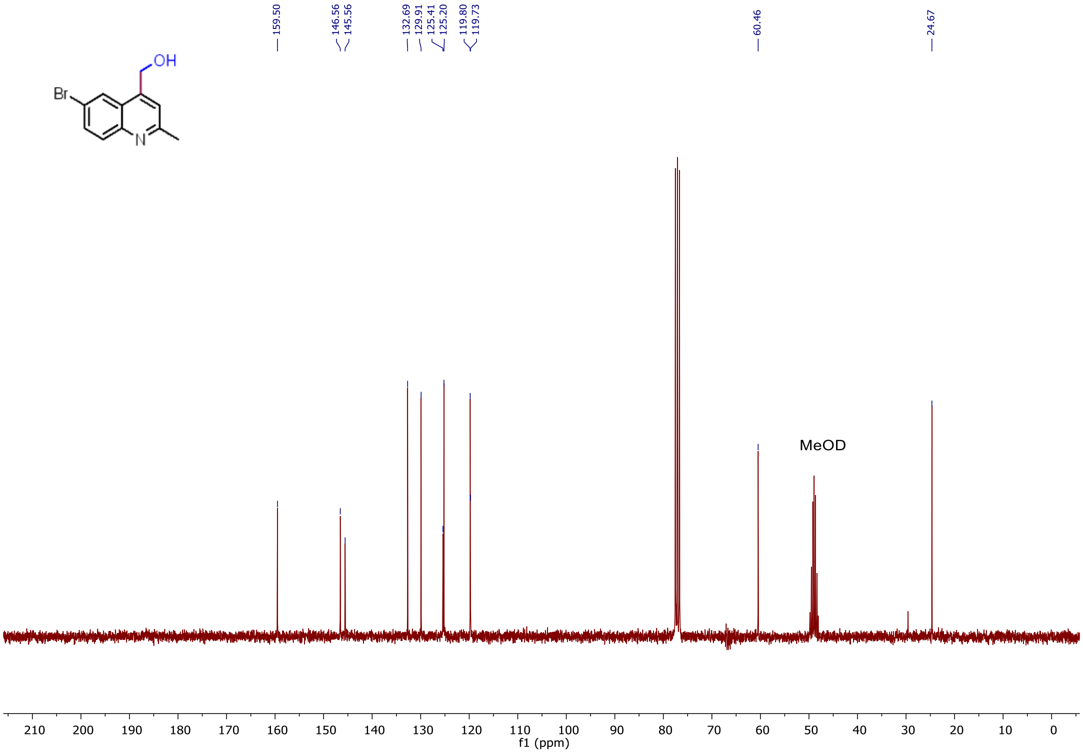

^{1}H NMR (400 MHz, $CDCl_3$) **(6)**

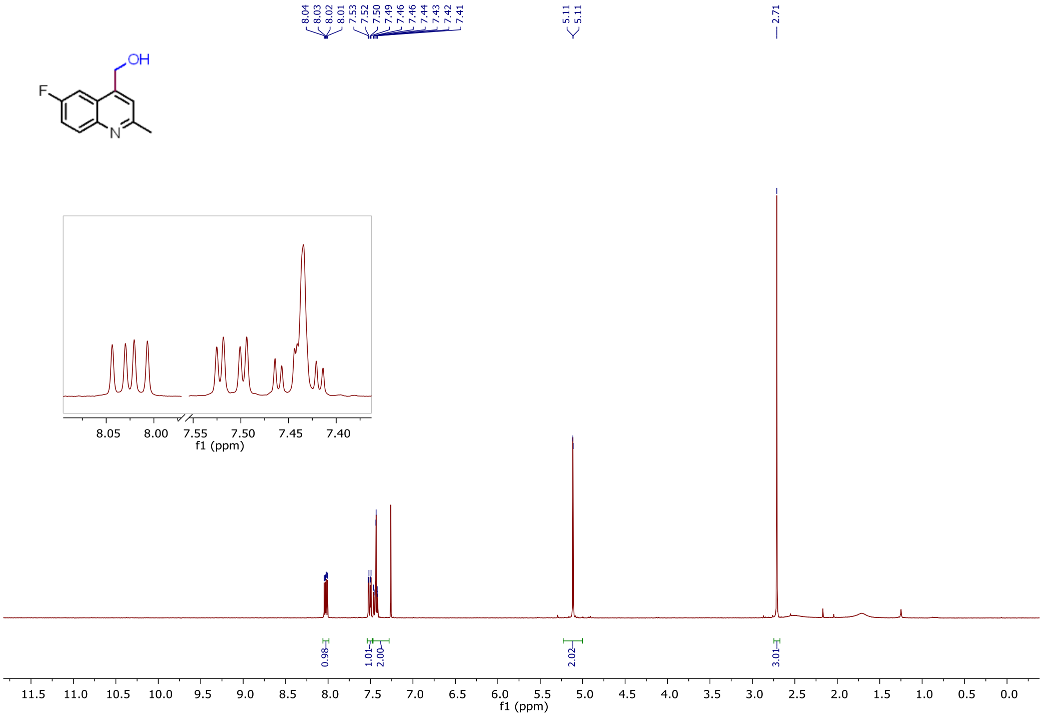

^{13}C NMR (101 MHz, $CDCl_3$) **(6)**

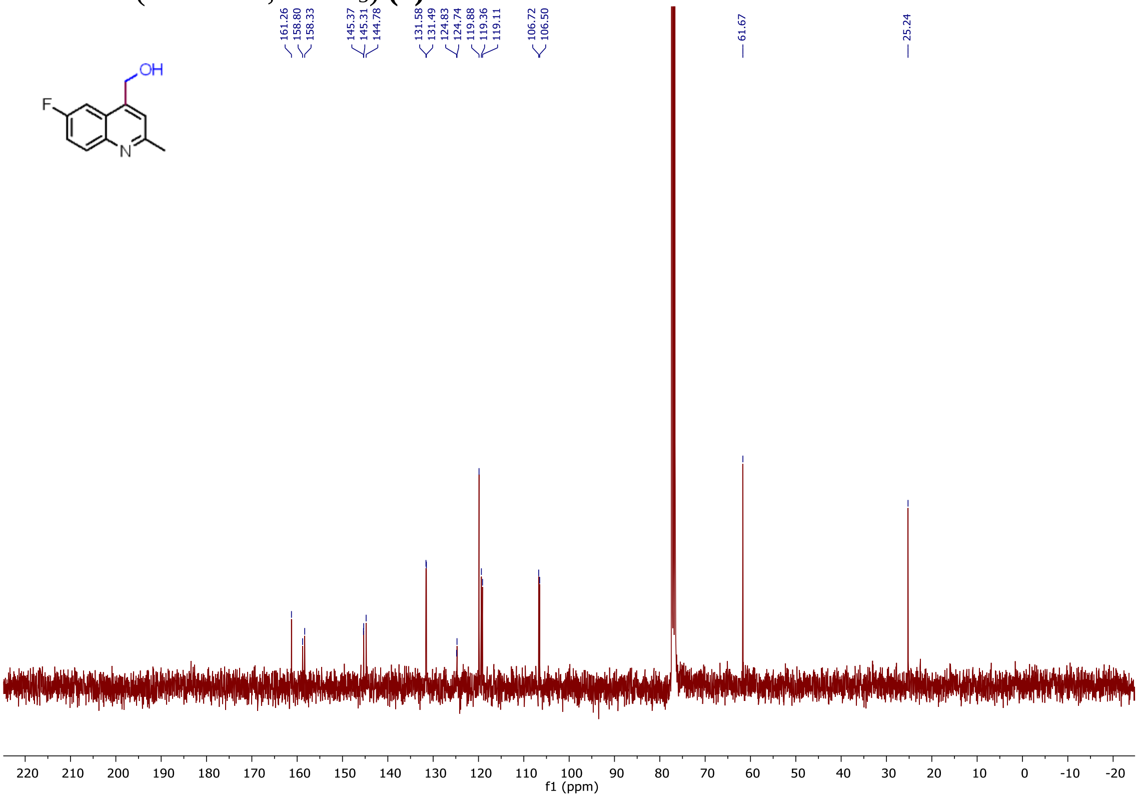

^{19}F NMR (101 MHz, $CDCl_3$) **(6)**

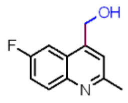

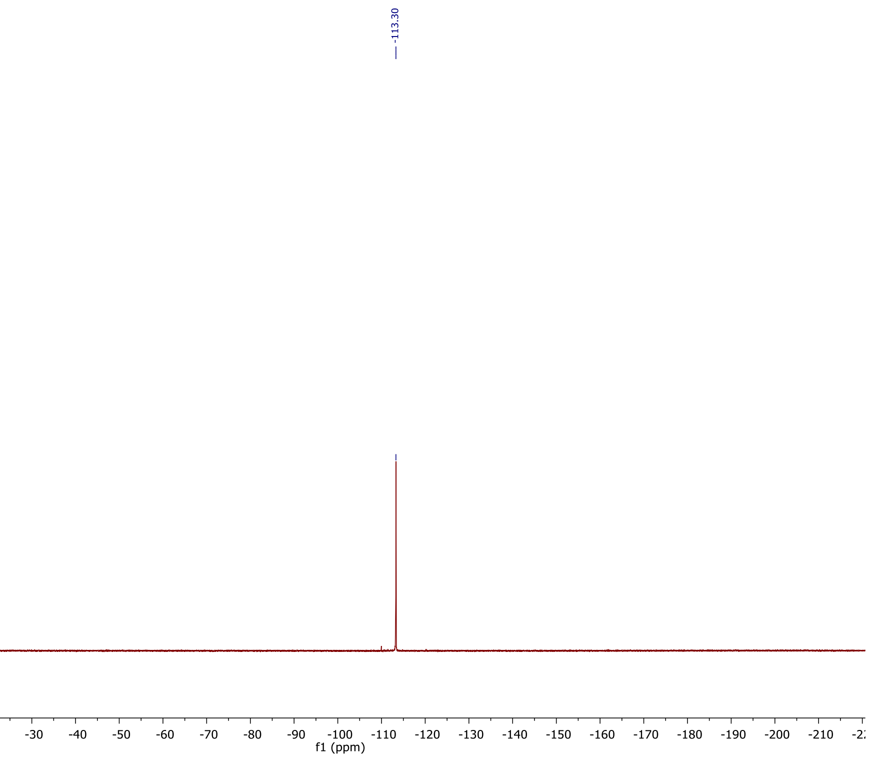

^{1}H NMR (400 MHz, CDCl$_3$) **(7)**

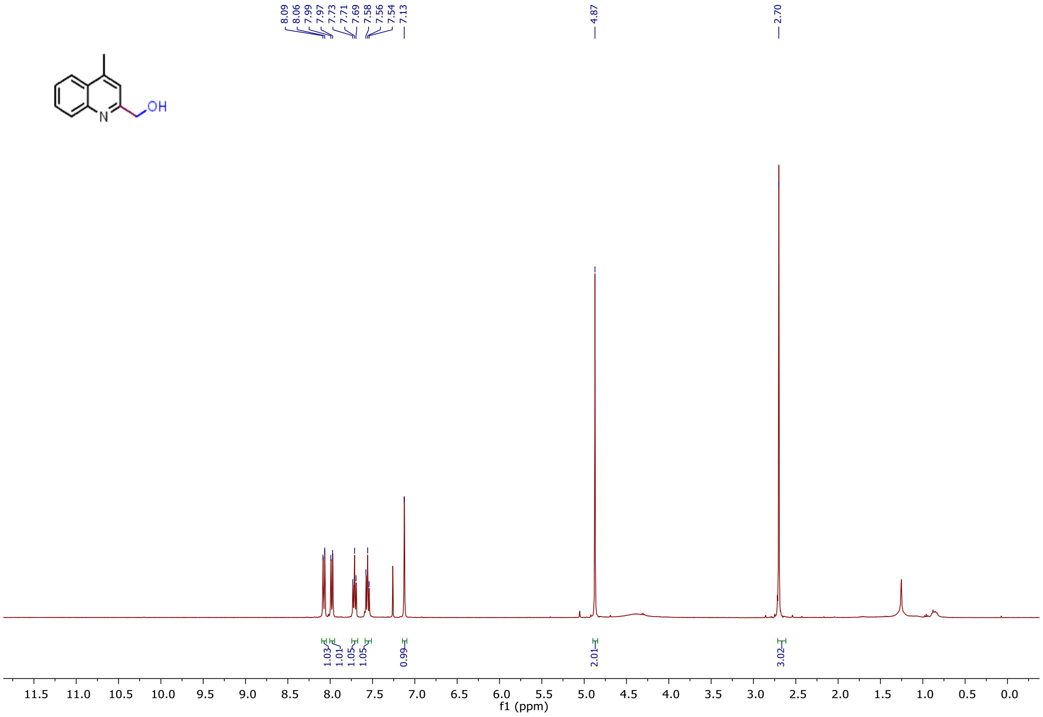

^{13}C NMR (101 MHz, CDCl$_3$) **(7)**

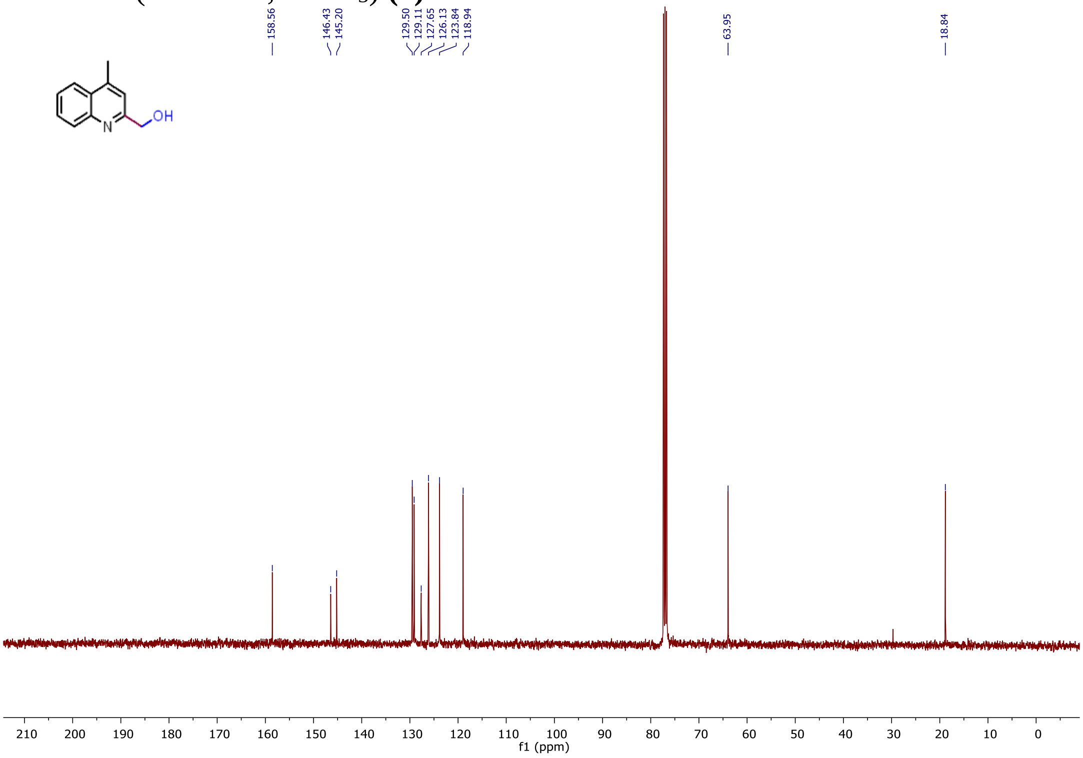

^{1}H NMR (400 MHz, CDCl$_3$) **(8)**

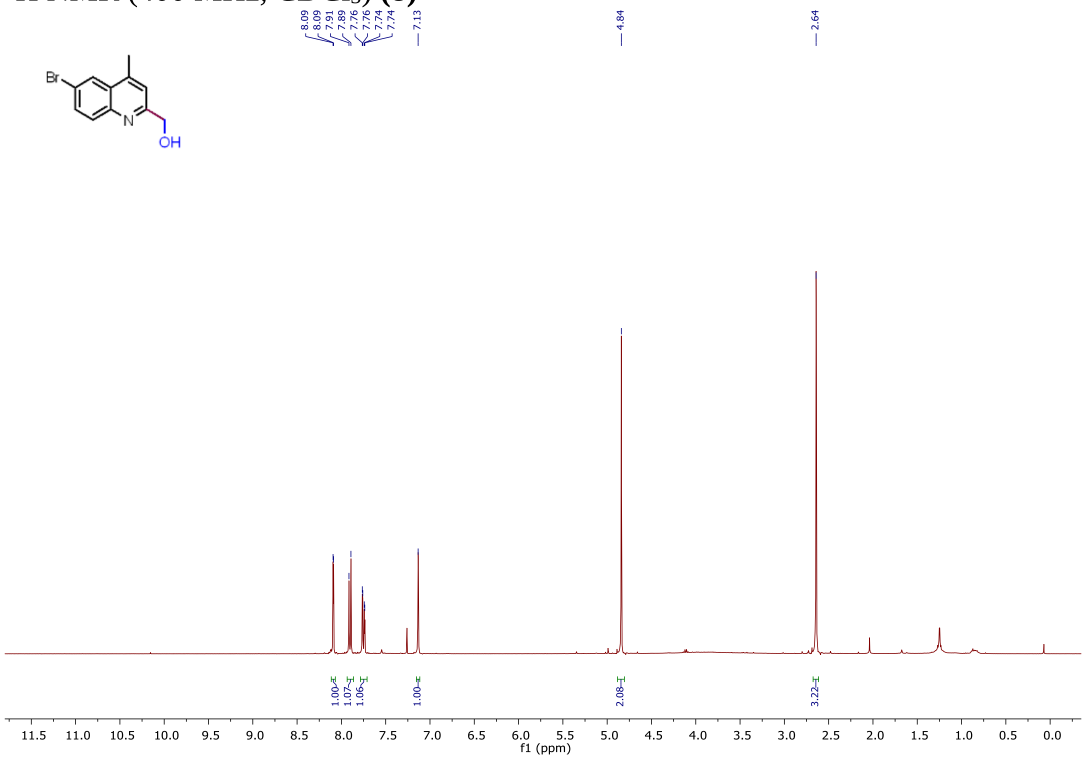

^{13}C NMR (101 MHz, CDCl$_3$) **(8)**

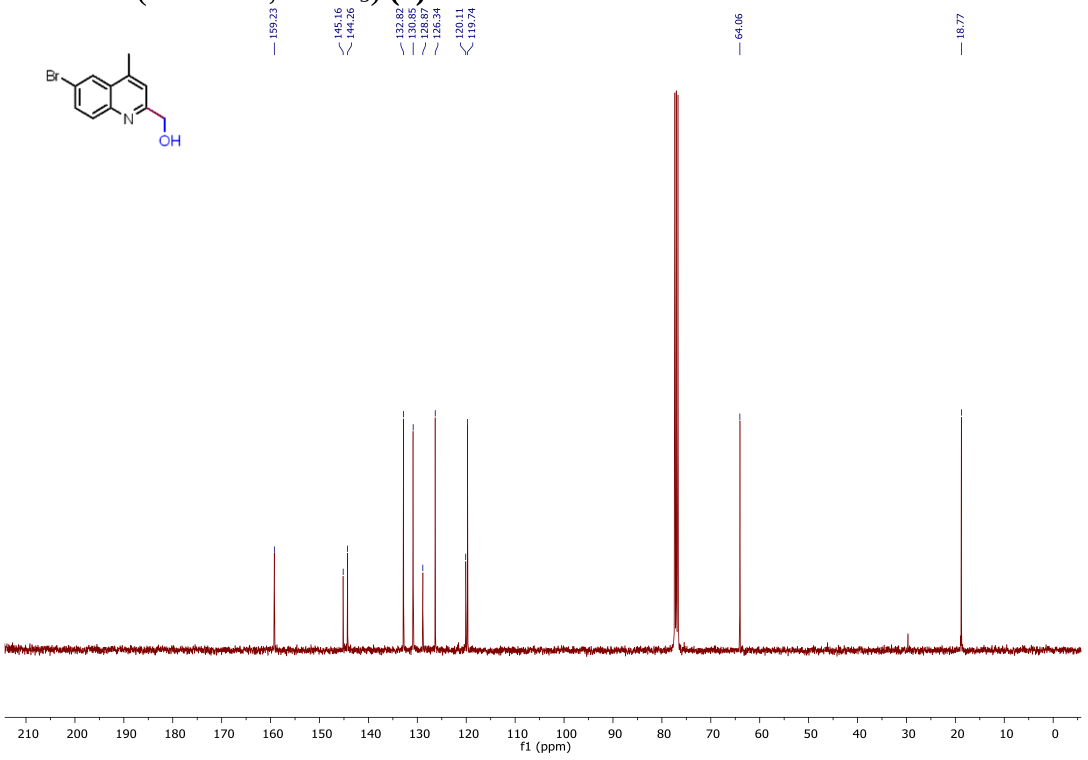

^{1}H NMR (400 MHz, $CDCl_3$) **(9)**

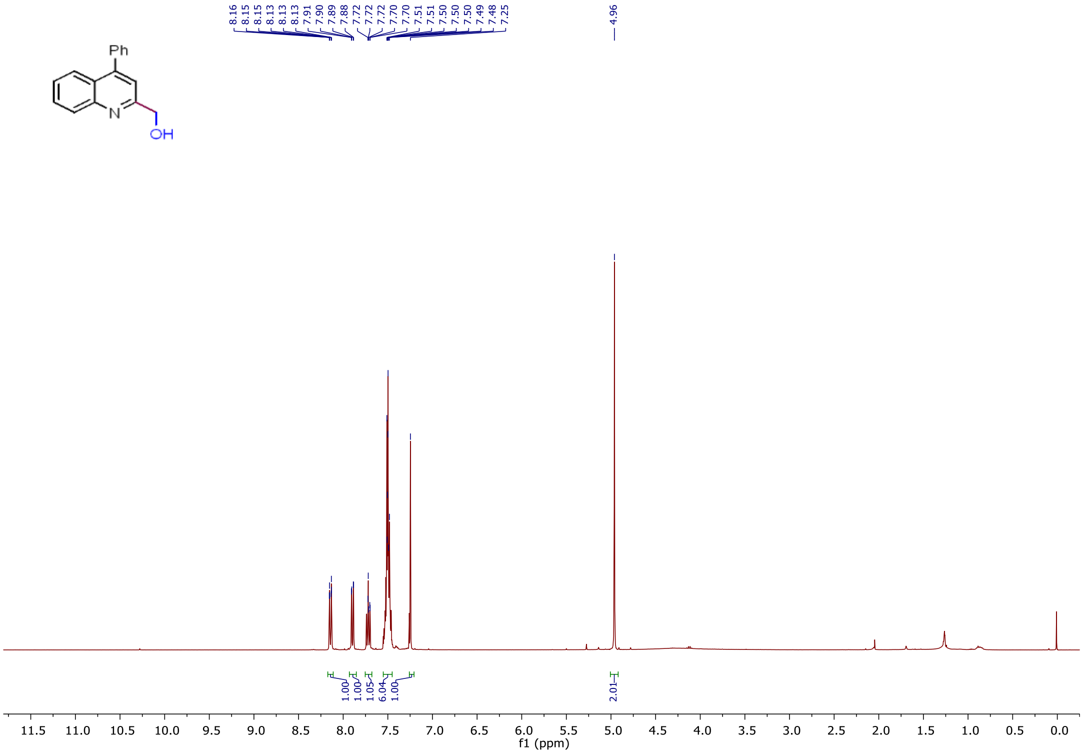

^{13}C NMR (101 MHz, $CDCl_3$) **(9)**

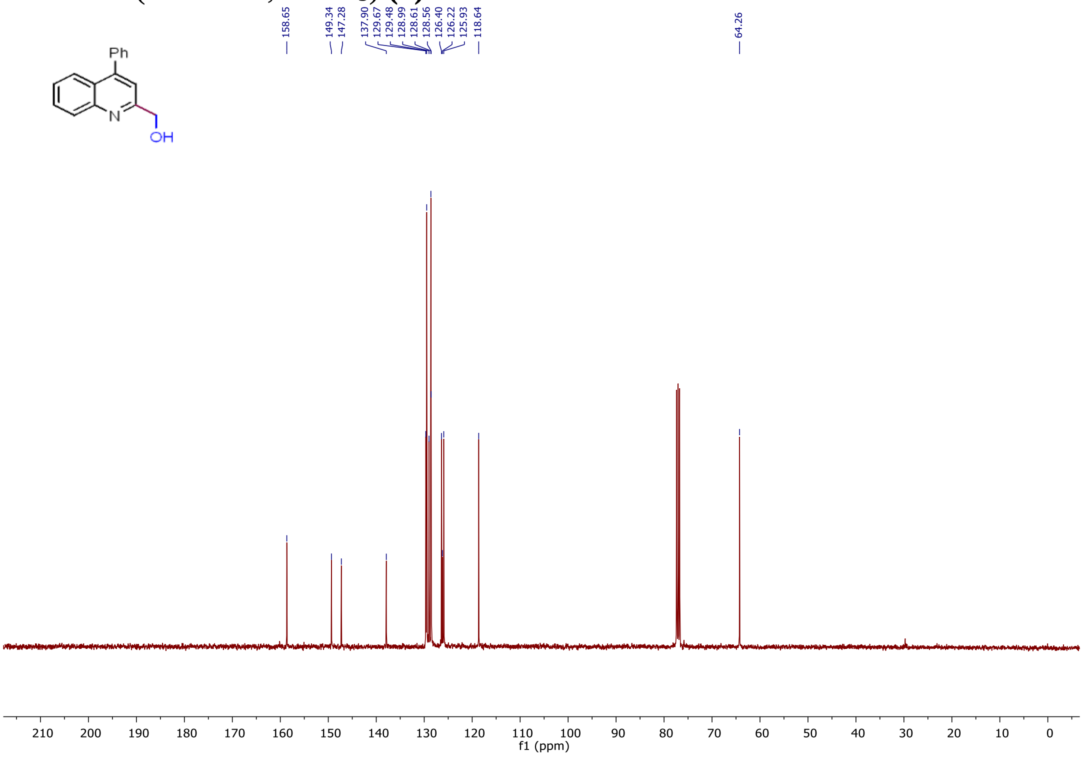

^{1}H NMR (400 MHz, $CDCl_3$) **(10)**

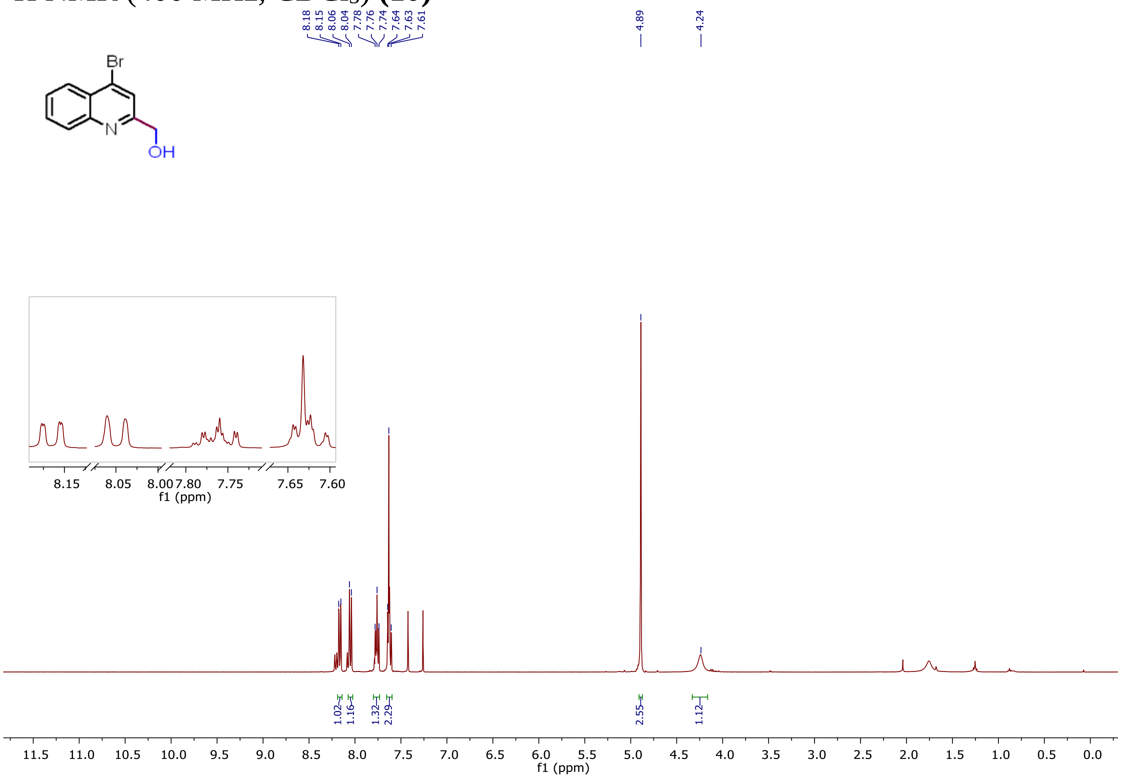

^{13}C NMR (101 MHz, $CDCl_3$) **(10)**

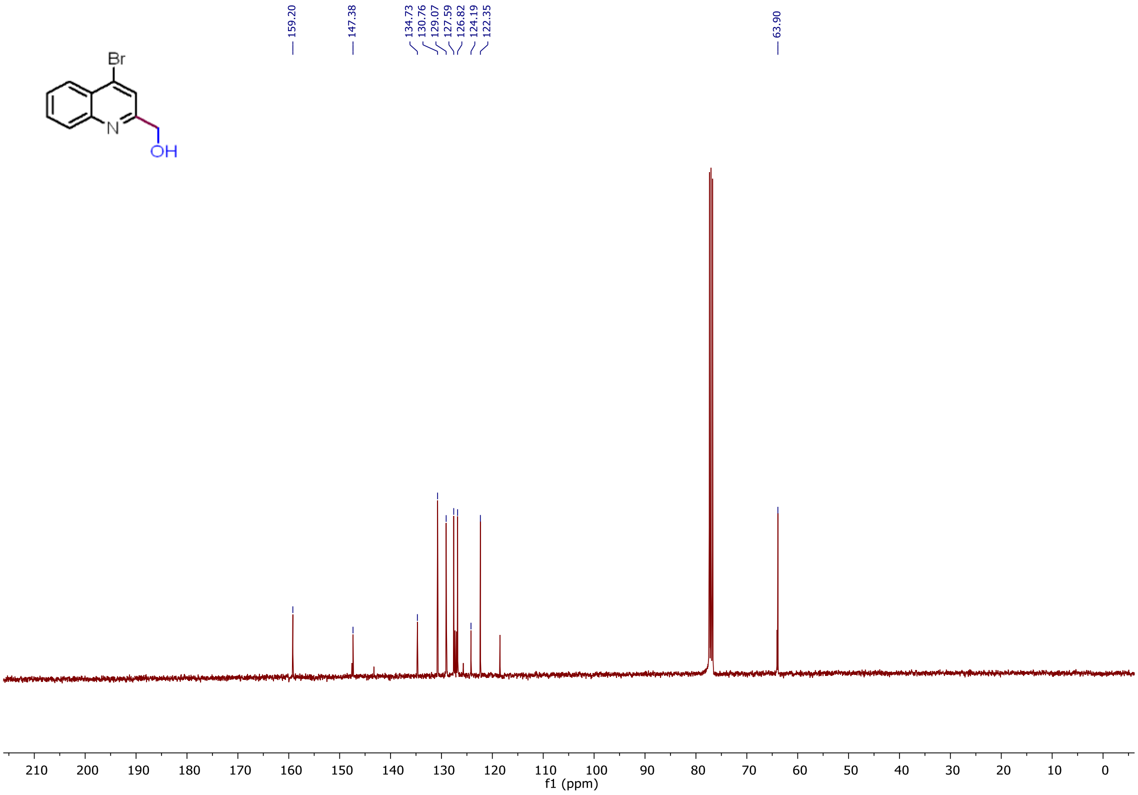

^{1}H NMR (400 MHz, $CDCl_3$) **(11)**

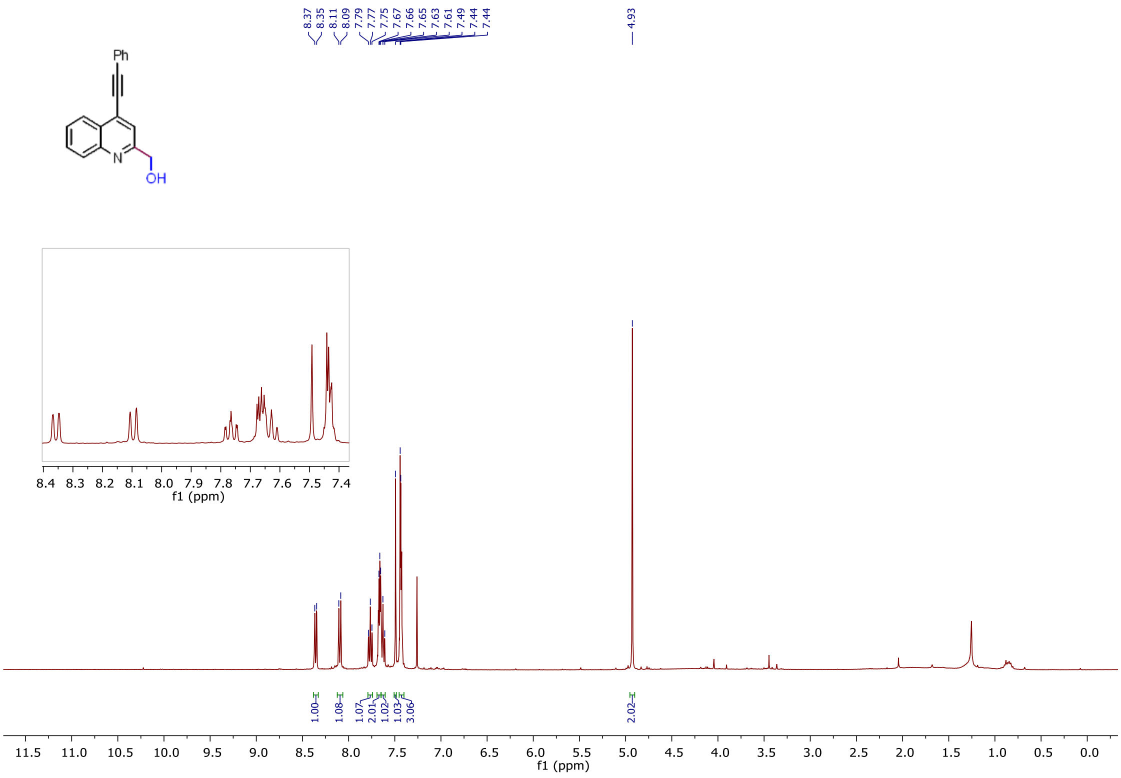

^{13}C NMR (101 MHz, $CDCl_3$) **(11)**

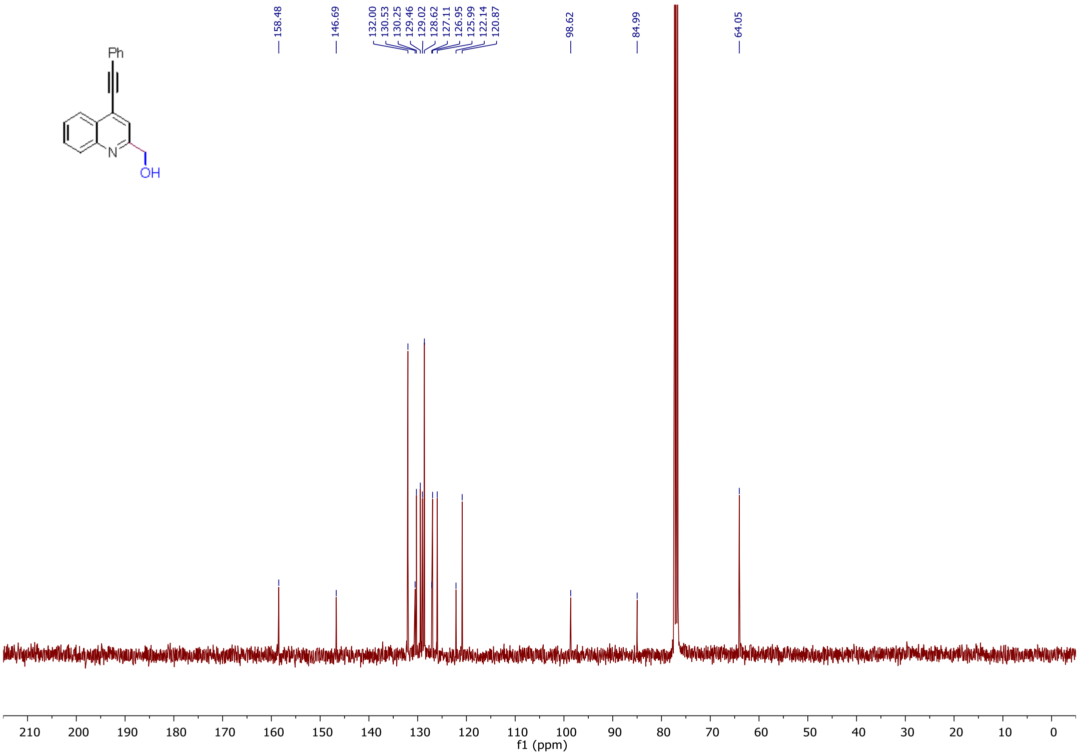

^{1}H NMR (400 MHz, MeOD-d4) **(12)**

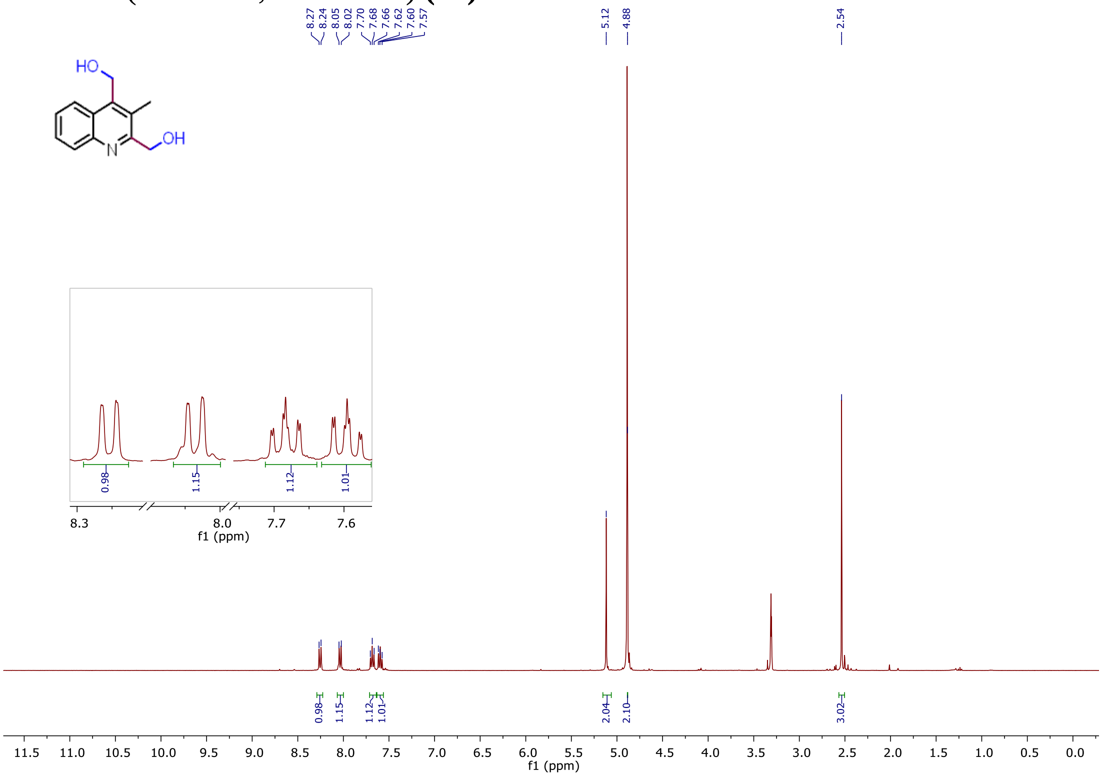

^{13}C NMR (101 MHz, DMSO-d_6) **(12)**

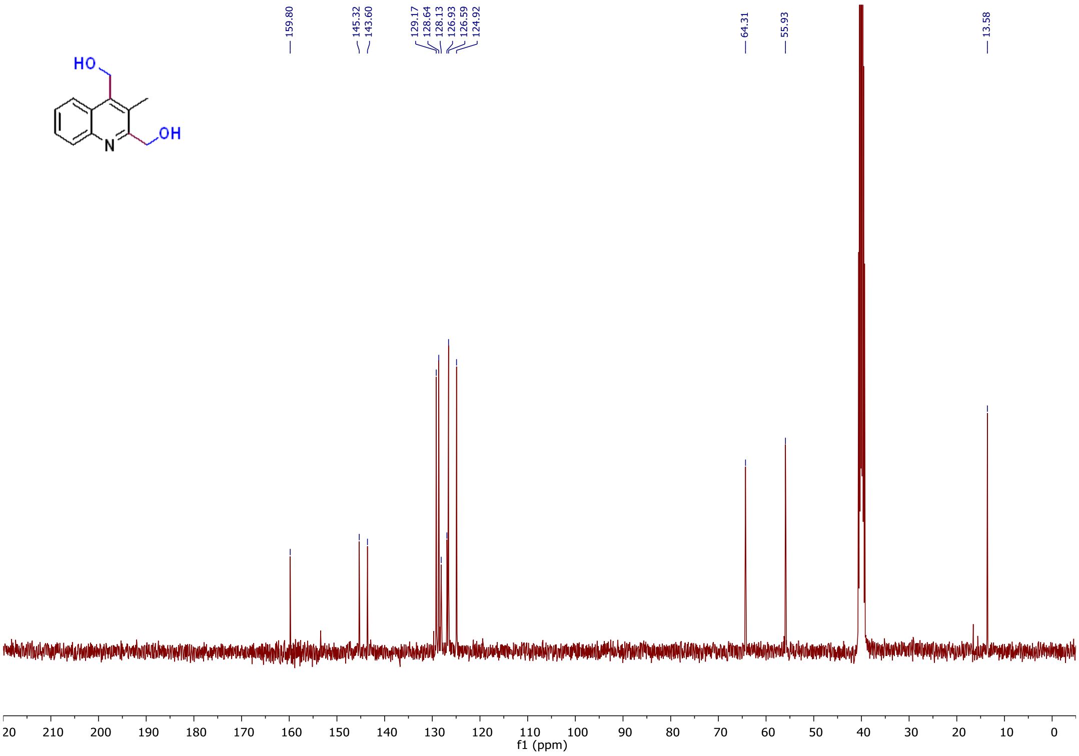

^{1}H NMR (400 MHz, DMSO-d6) **(13)**

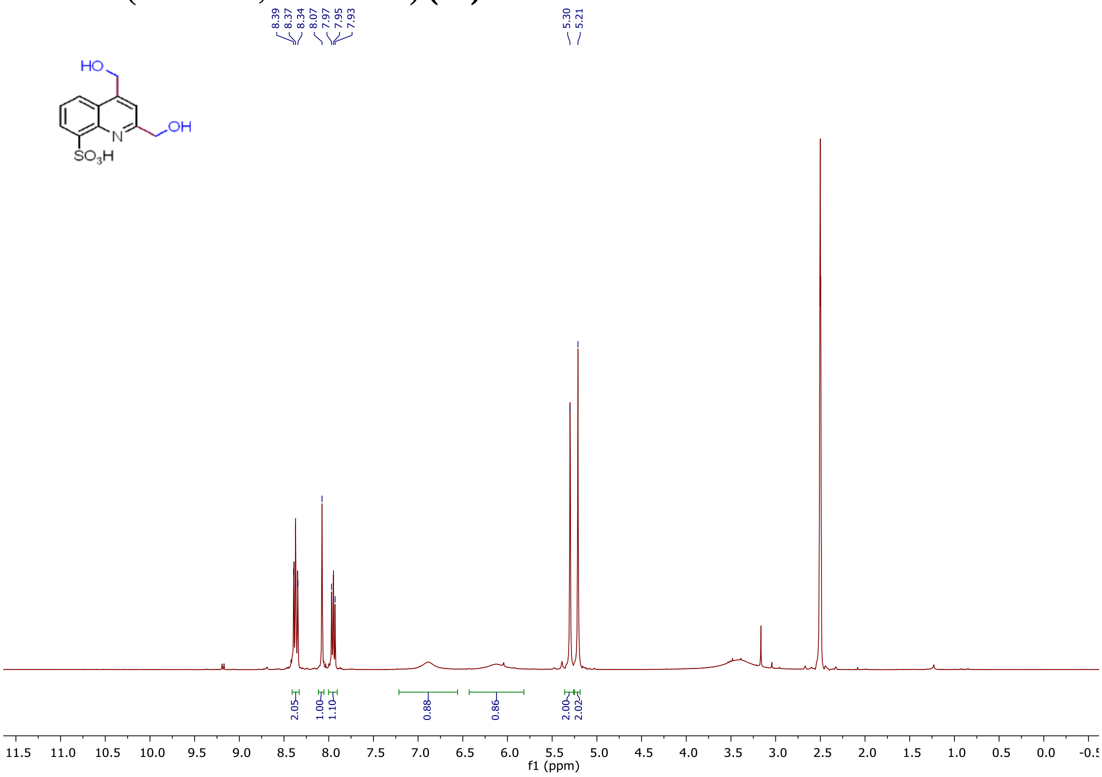

^{13}C NMR (101 MHz, DMSO-d6) **(13)**

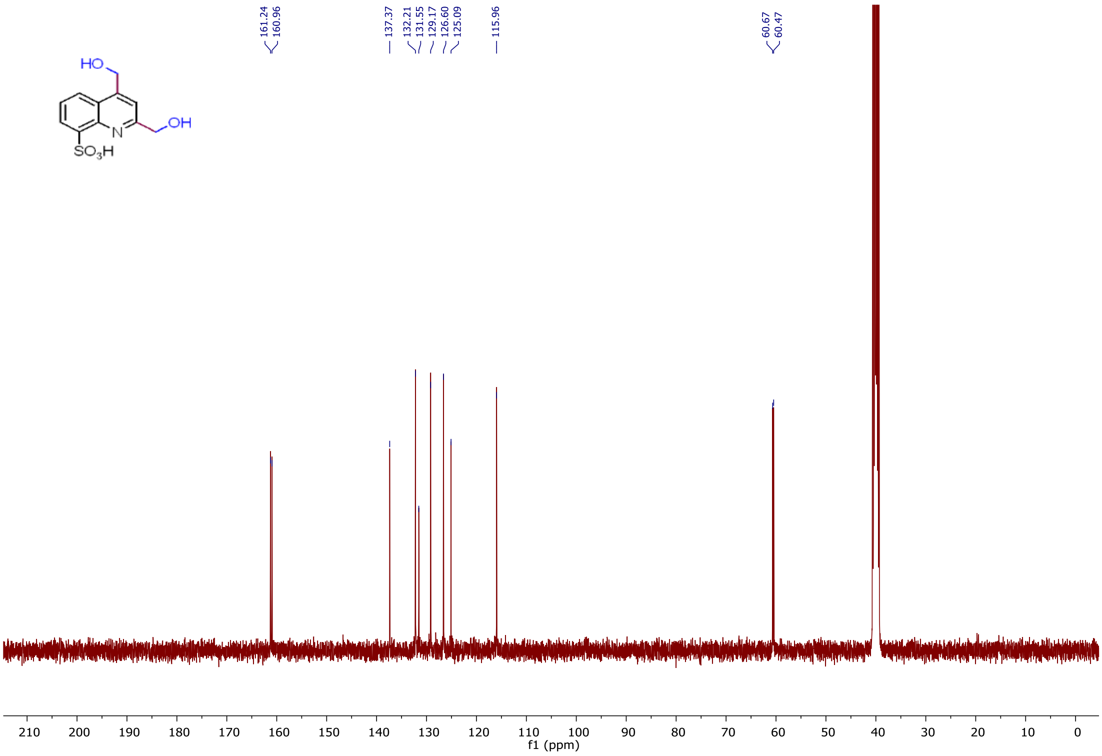

^{1}H NMR (400 MHz, DMSO-d$_6$) **(14)**

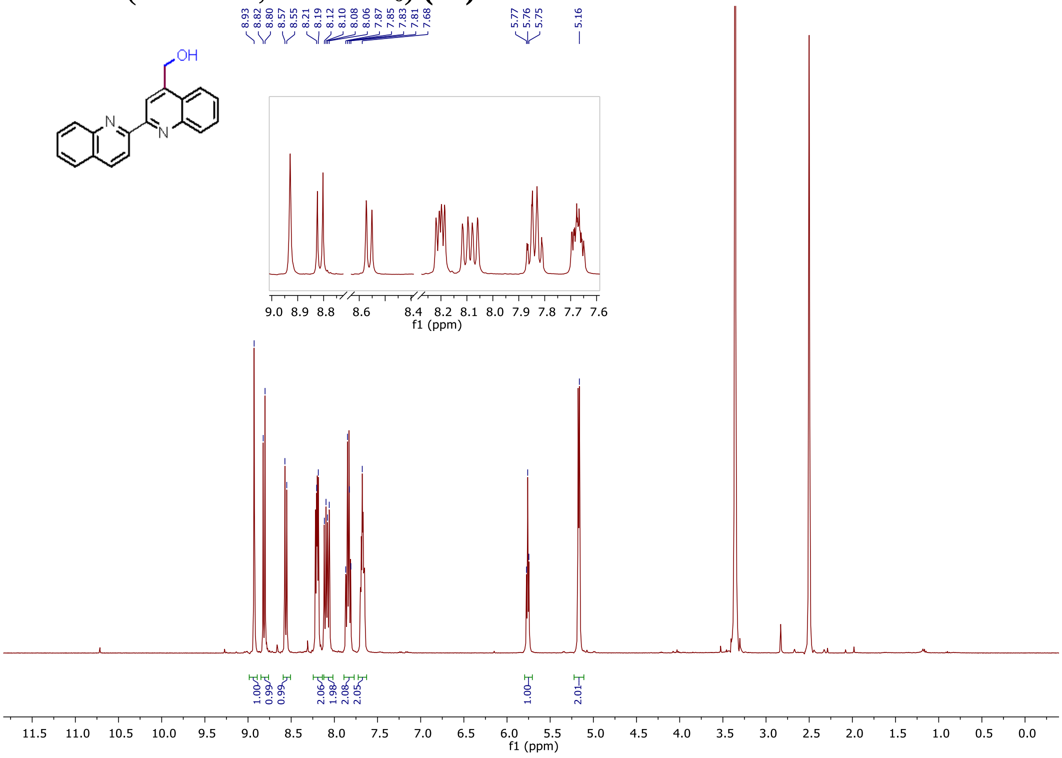

^{13}C NMR (101 MHz, DMSO-d$_6$) **(14)**

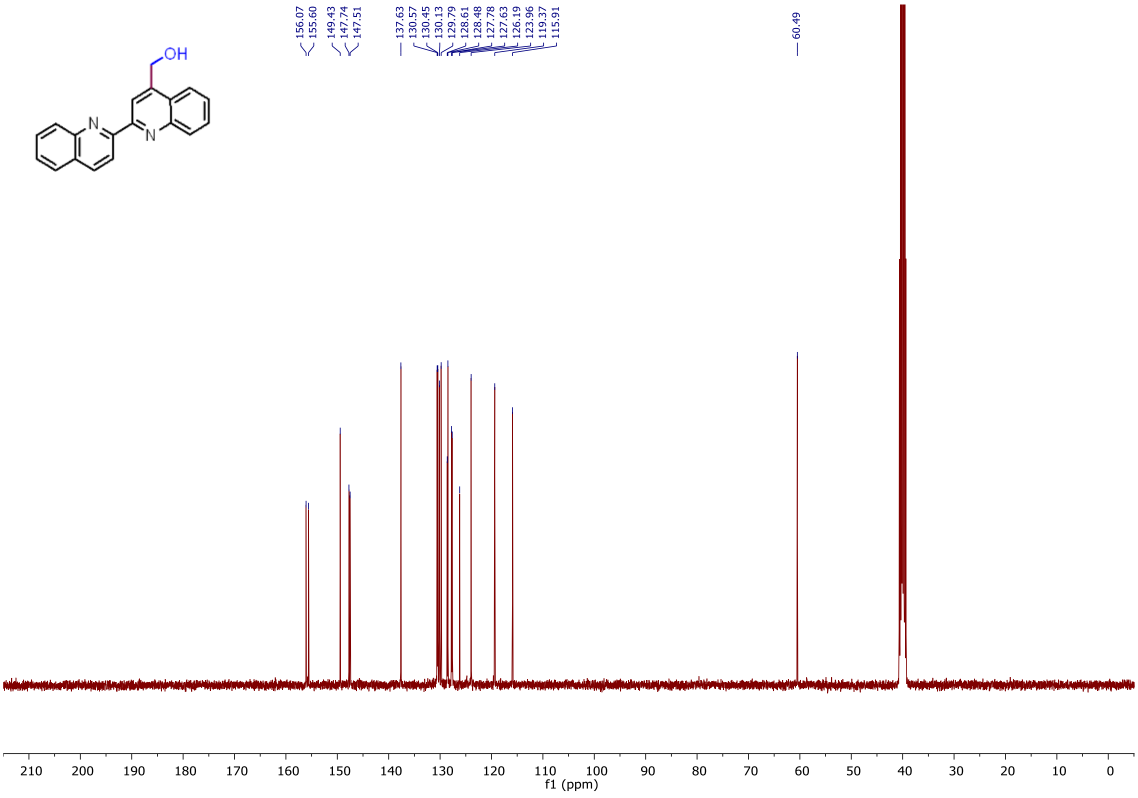

^{1}H NMR (400 MHz, $CDCl_3$) **(15)**

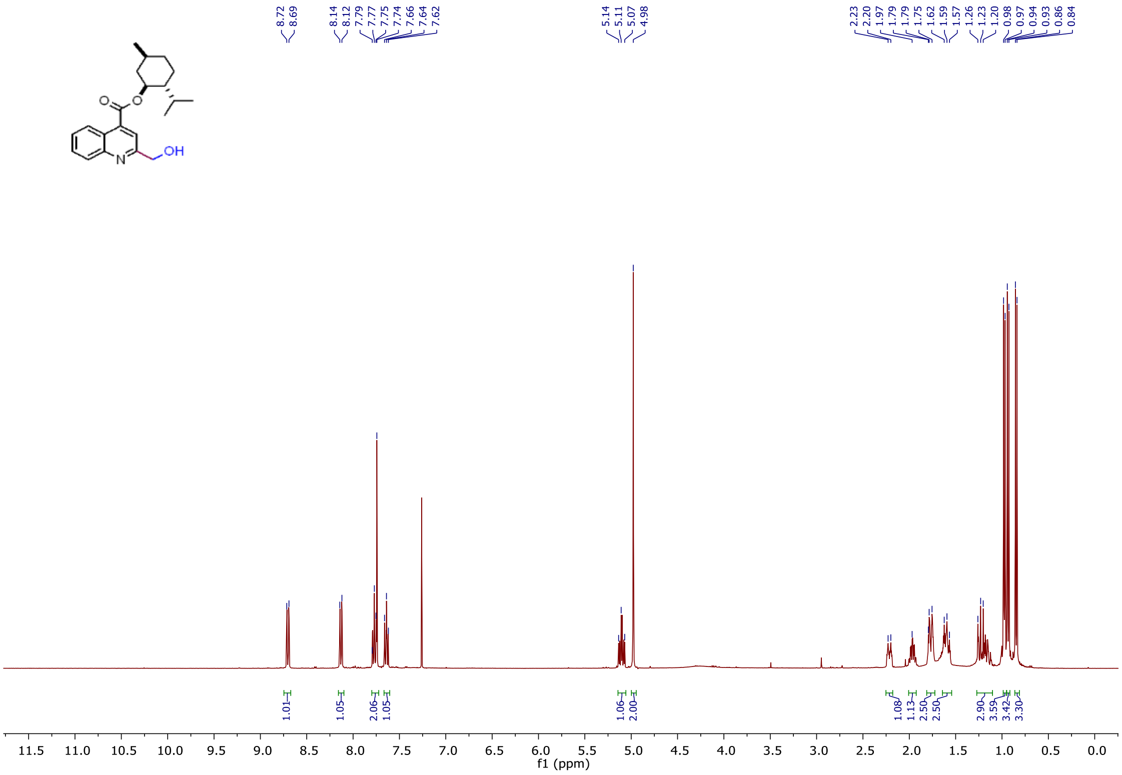

^{13}C NMR (101 MHz, $CDCl_3$) **(15)**

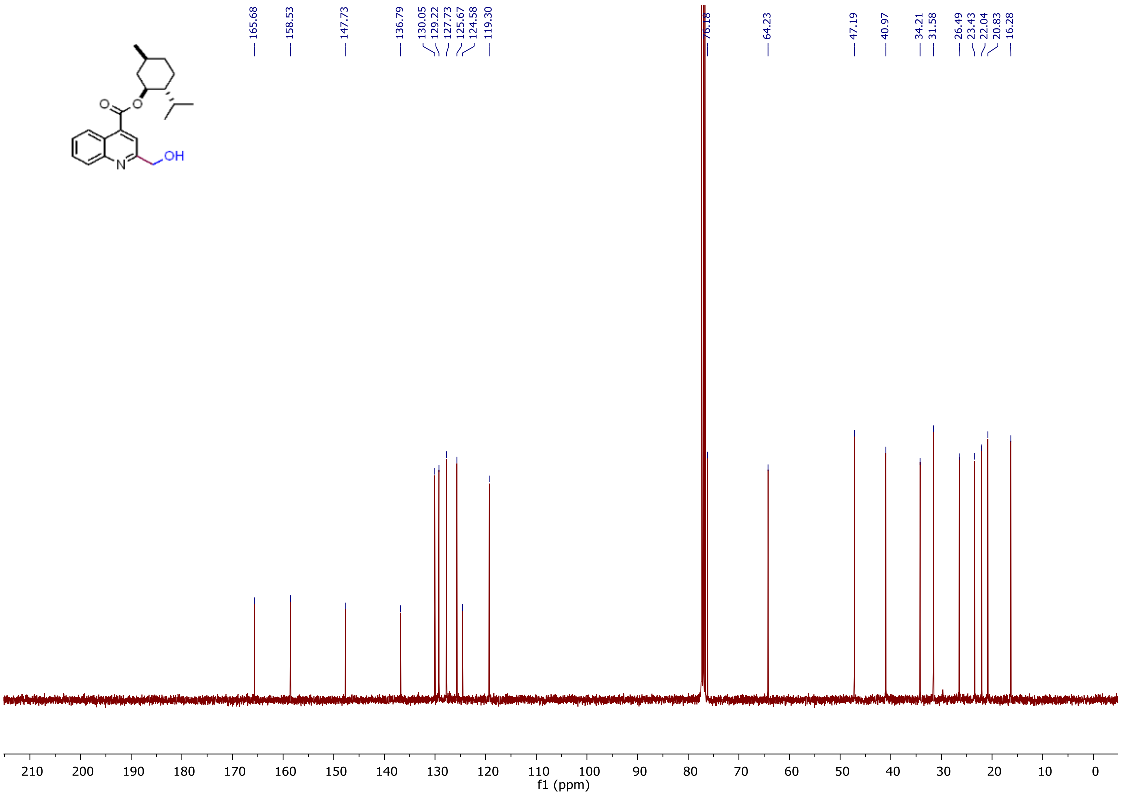

^{1}H NMR (400 MHz, $CDCl_3$) **(16a)**

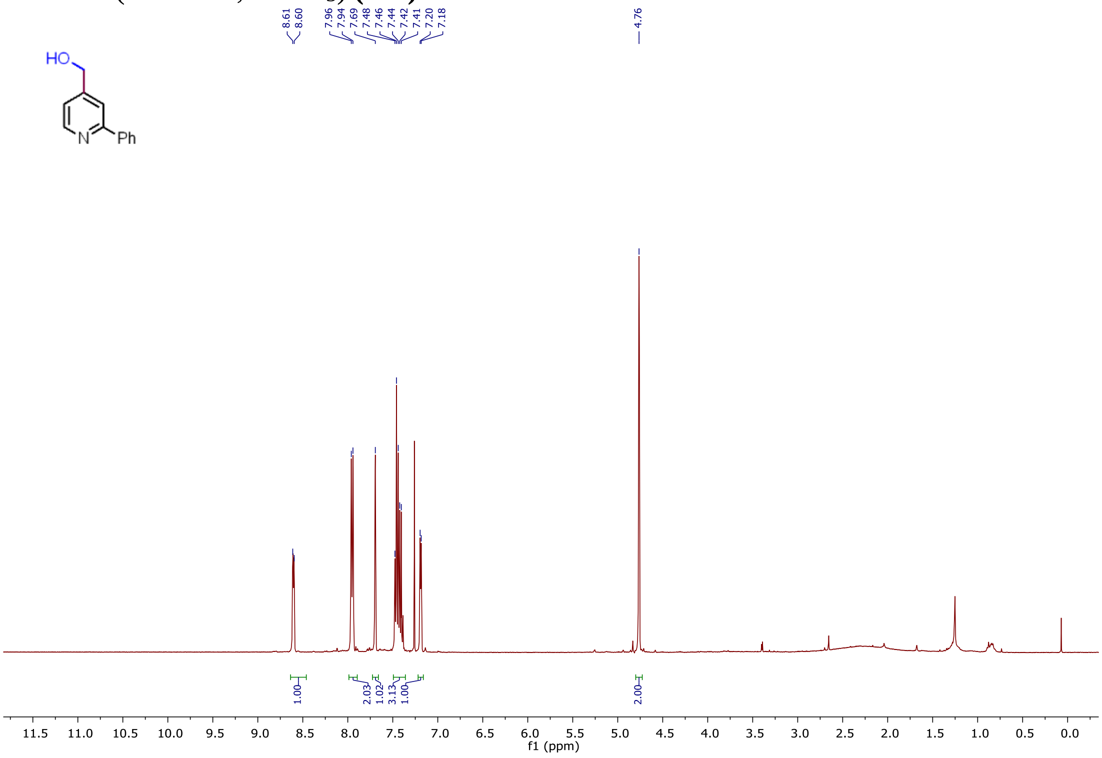

^{13}C NMR (101 MHz, $CDCl_3$) **(16a)**

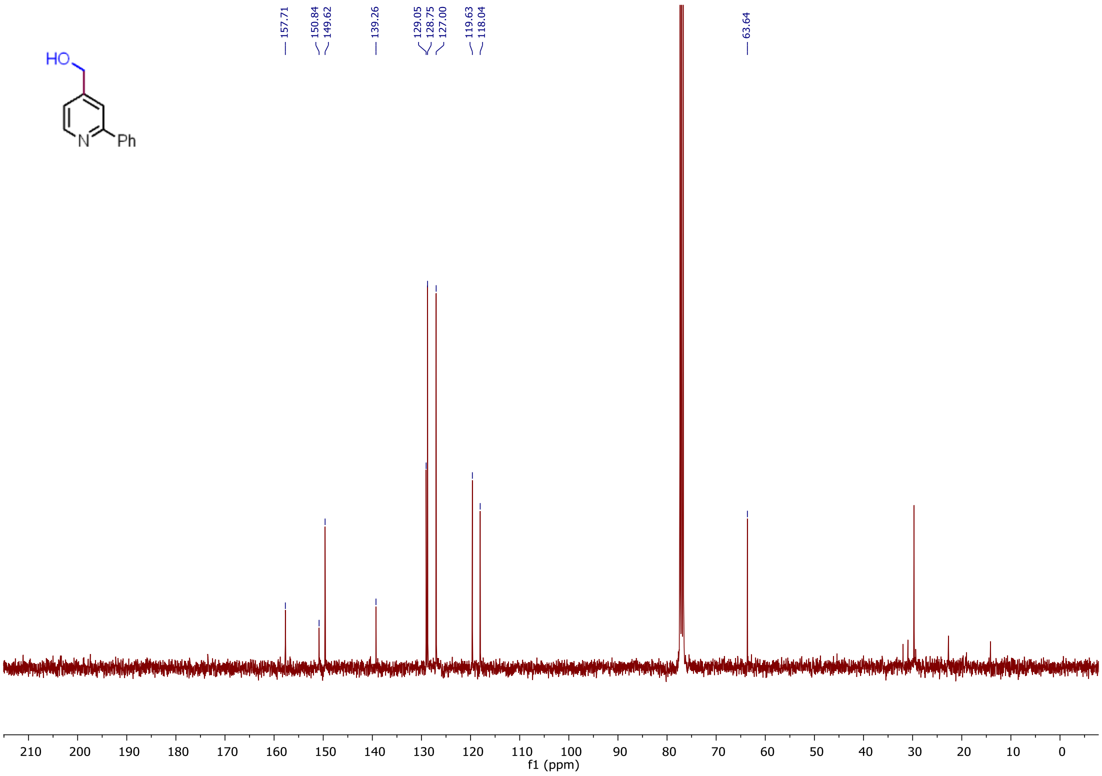

^{1}H NMR (400 MHz, $CDCl_3$) **(16b)**

A minor amount of this isomer was obtained, which complicates obtaining clean samples for the NMR spectra

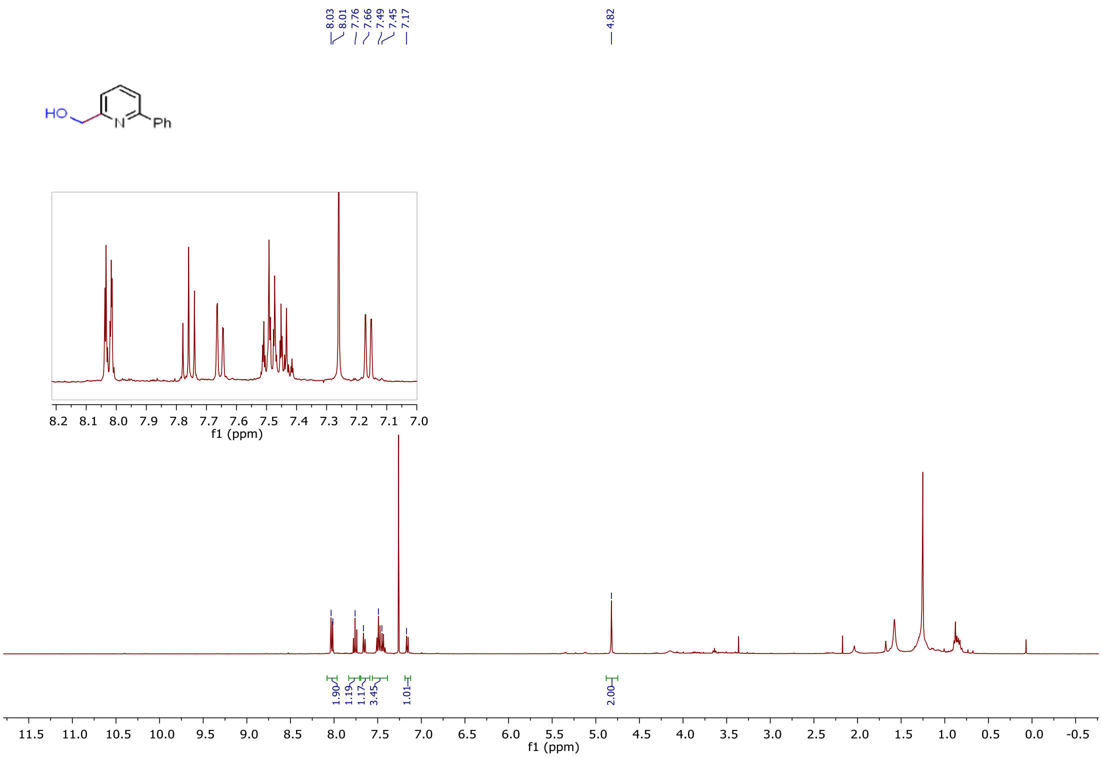

^{13}C NMR (101 MHz, $CDCl_3$) **(16b)**

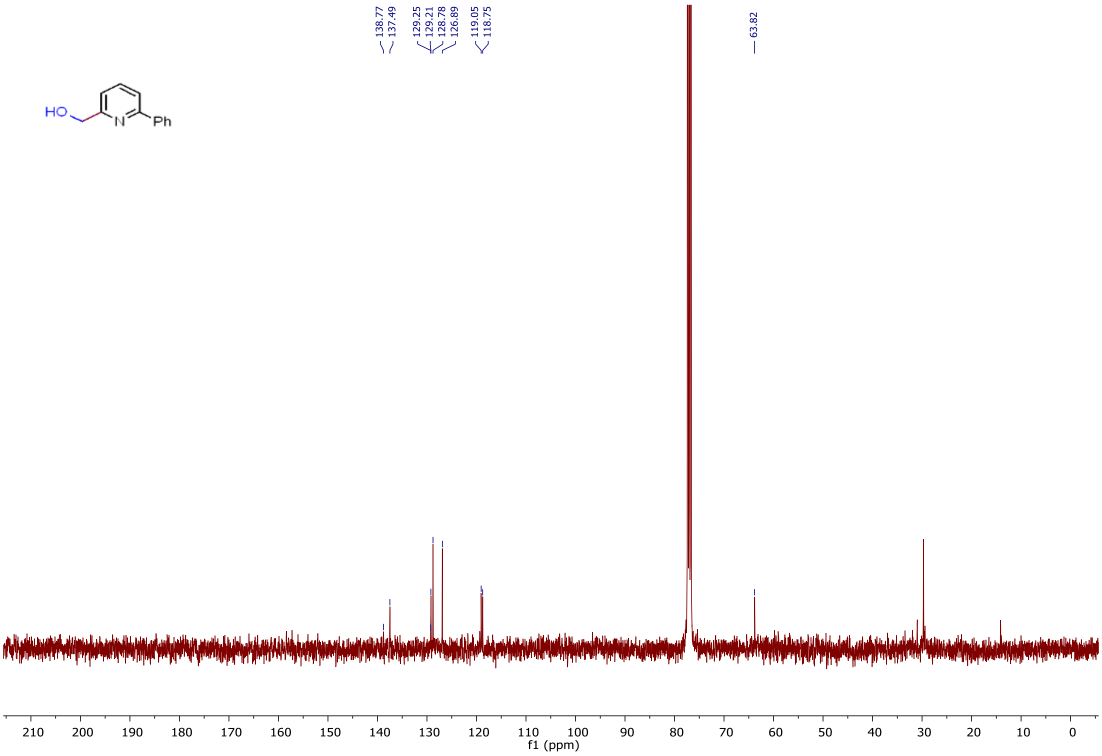

^{1}H NMR (400 MHz, $CDCl_3$) **(17)**

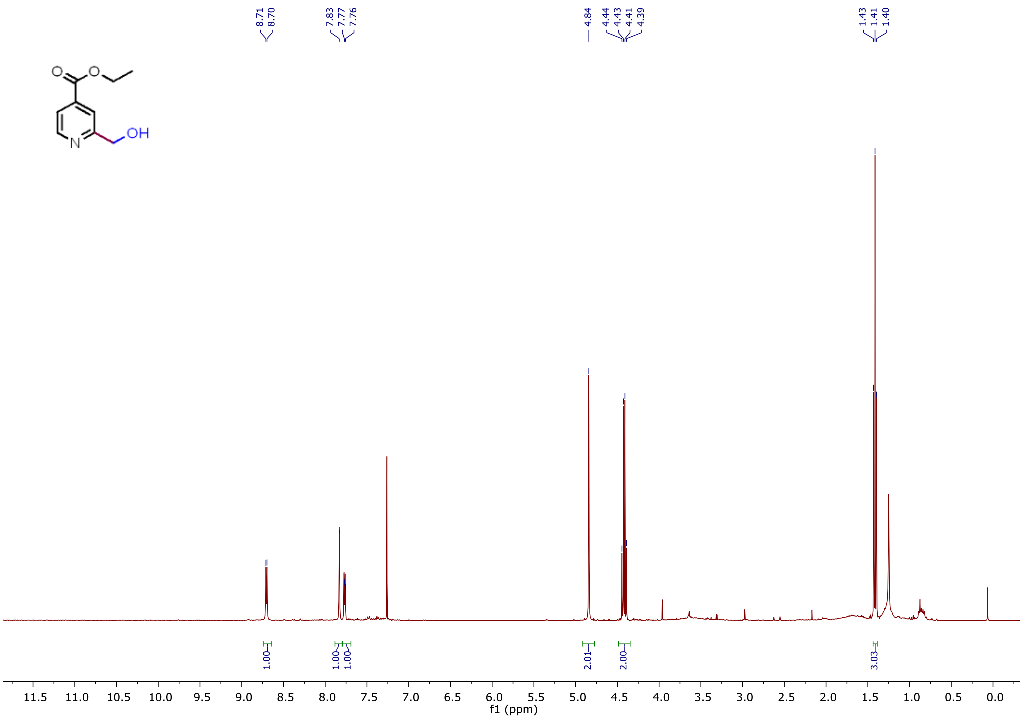

^{13}C NMR (101 MHz, $CDCl_3$) **(17)**

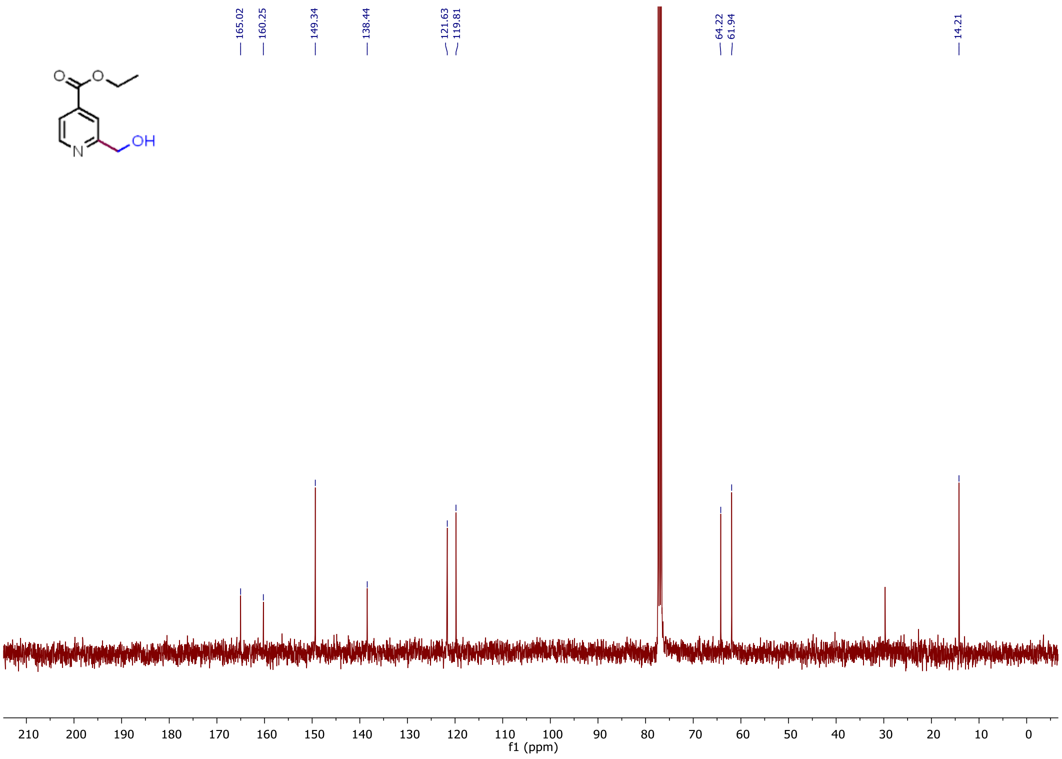

^{1}H NMR (400 MHz, $CDCl_3$) **(18)**

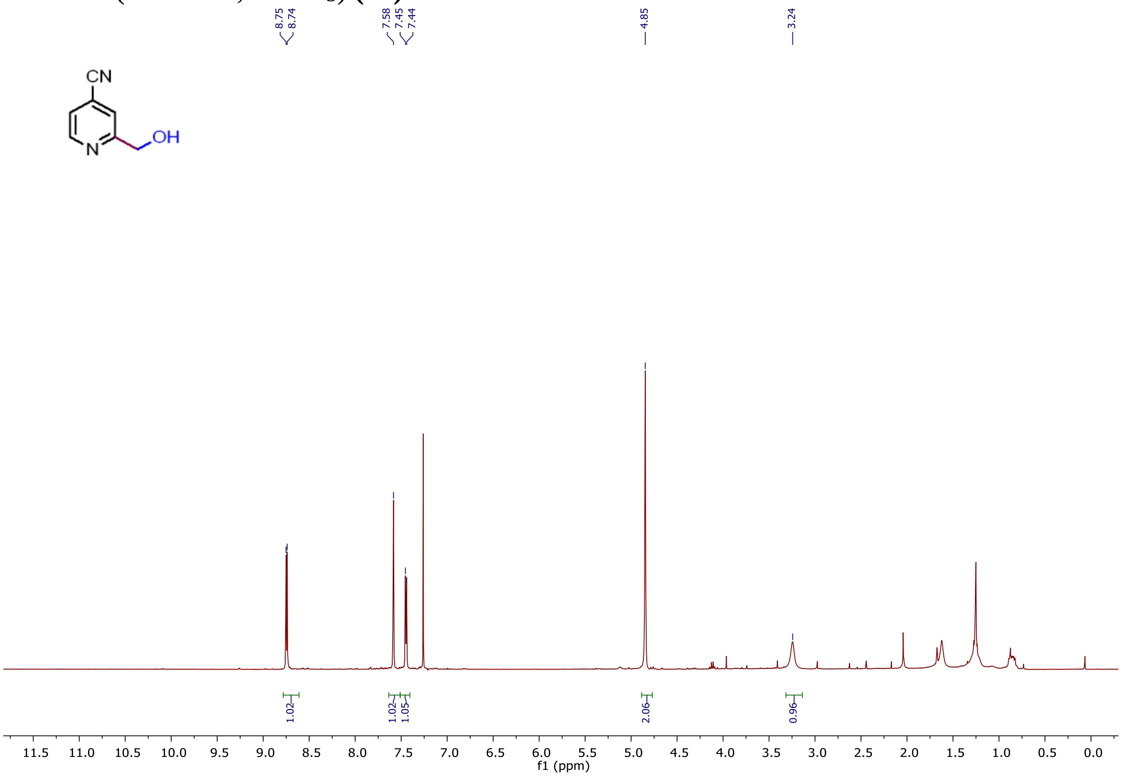

^{13}C NMR (101 MHz, $CDCl_3$) **(18)**

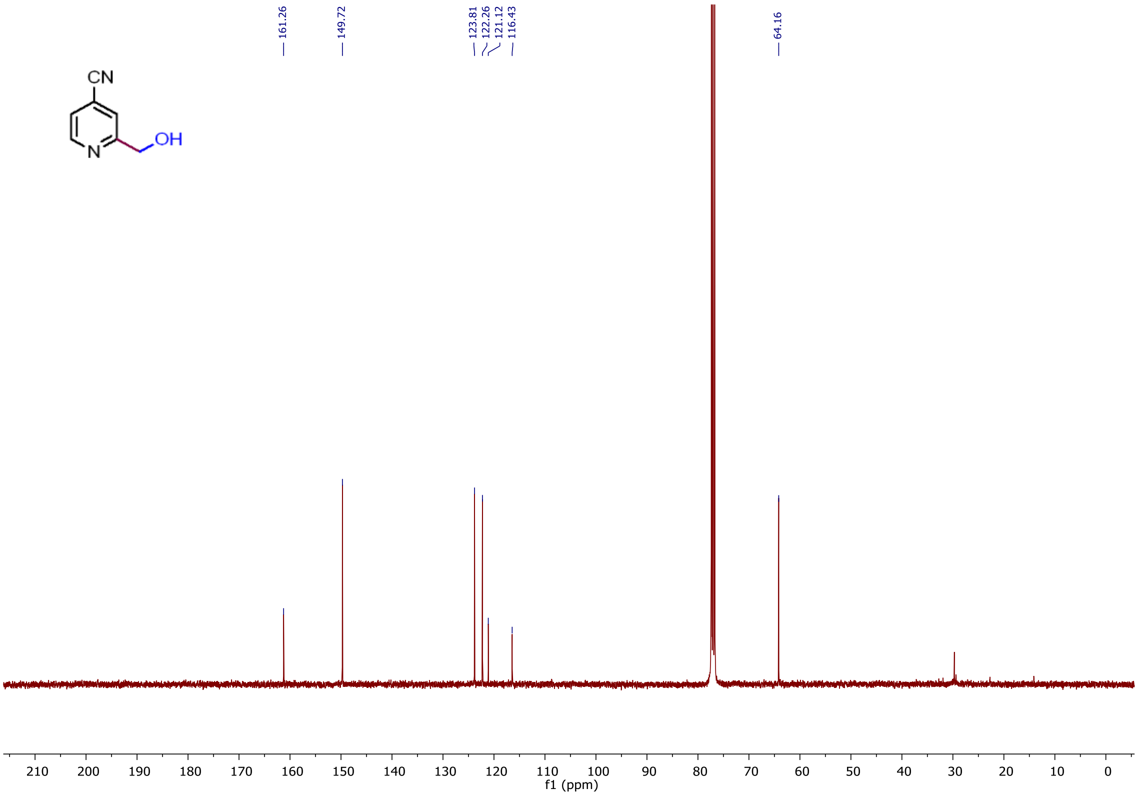

^{1}H NMR (400 MHz, $CDCl_3$) **(19)**

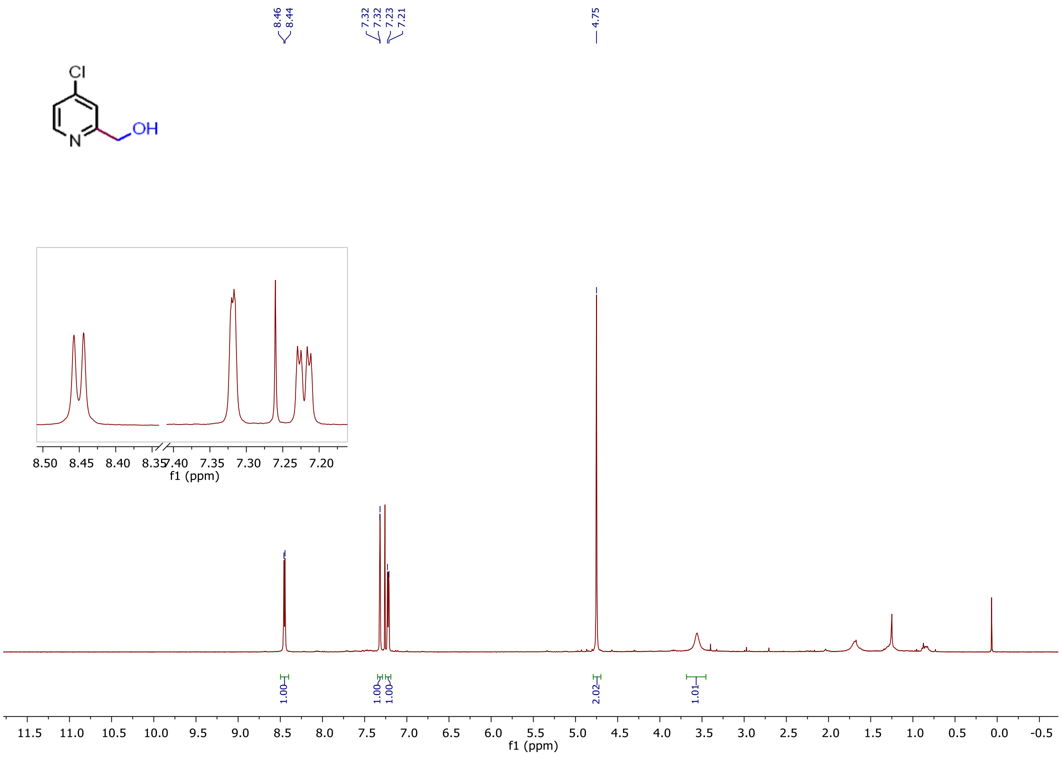

^{13}C NMR (101 MHz, $CDCl_3$) **(19)**

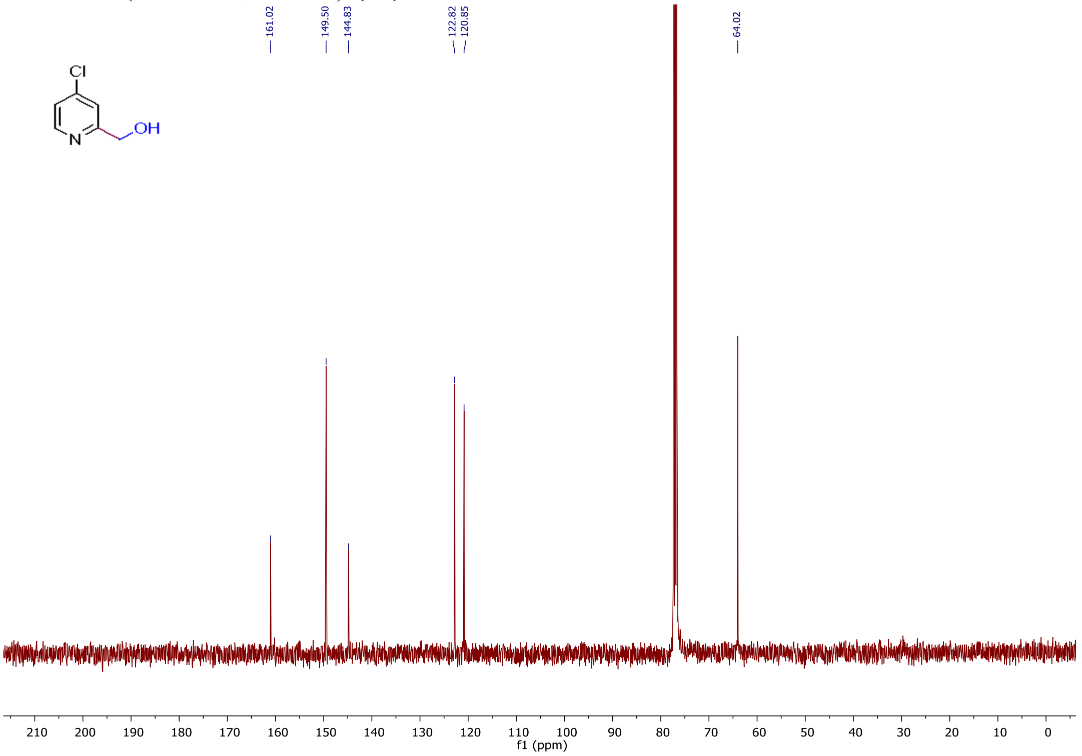

^{1}H NMR (300 MHz, $CDCl_3$) **(20)**

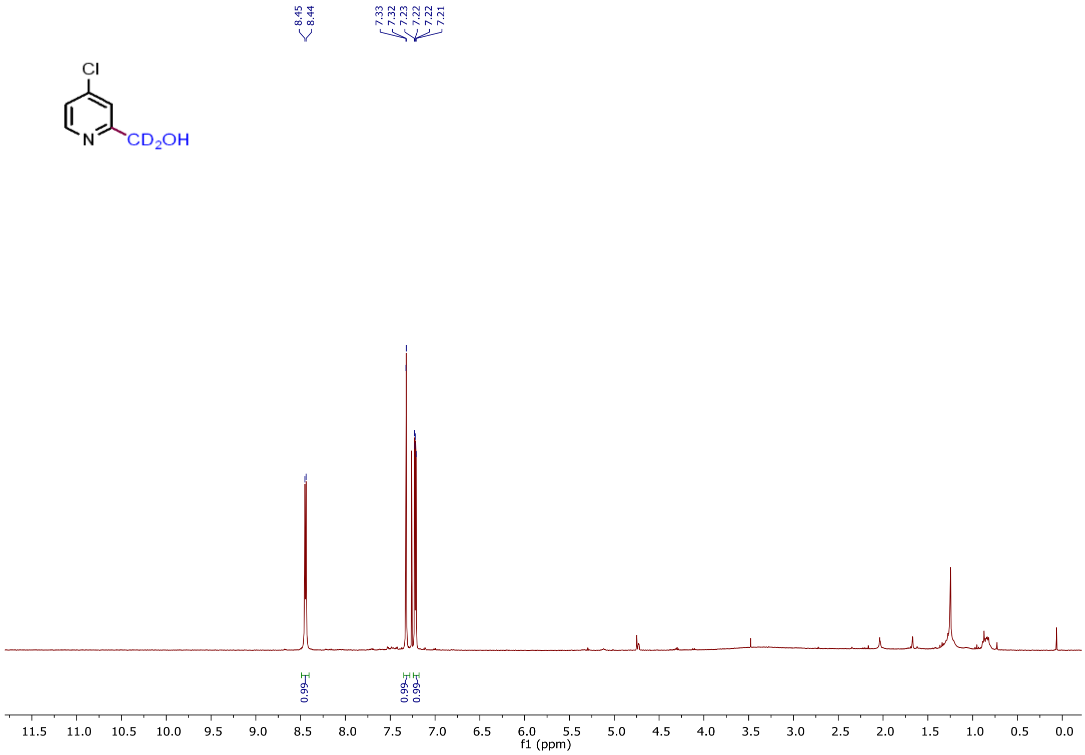

^{13}C NMR (101 MHz, $CDCl_3$) **(20)**

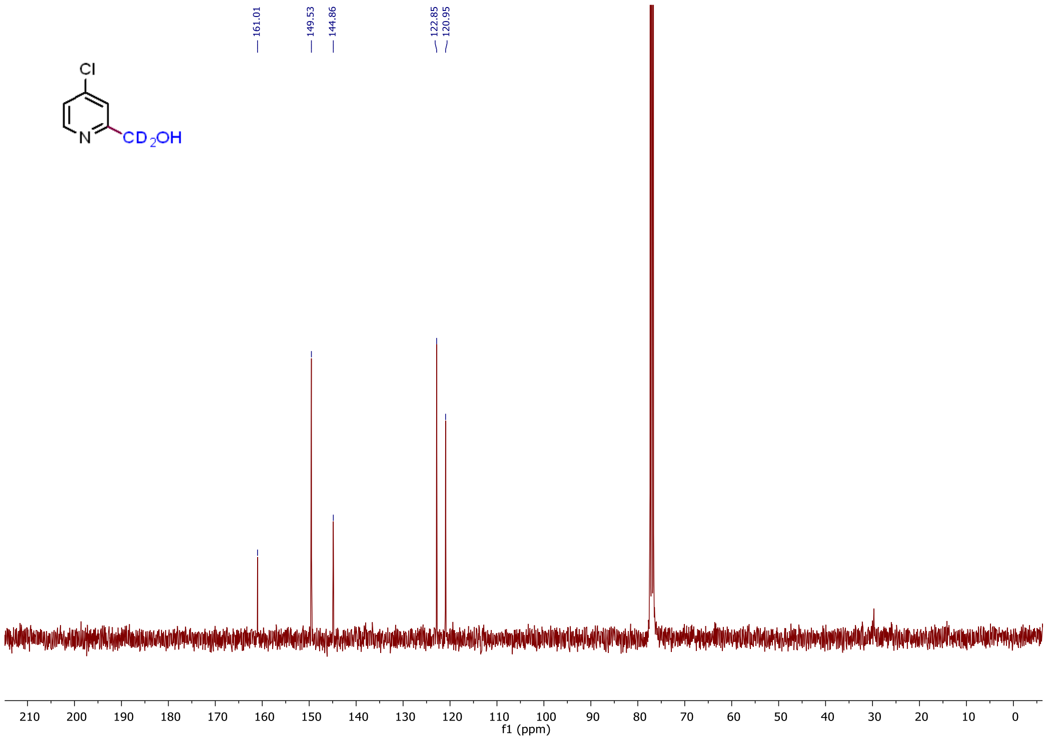

^{1}H NMR (400 MHz, DMSO-d_6) **(21)**

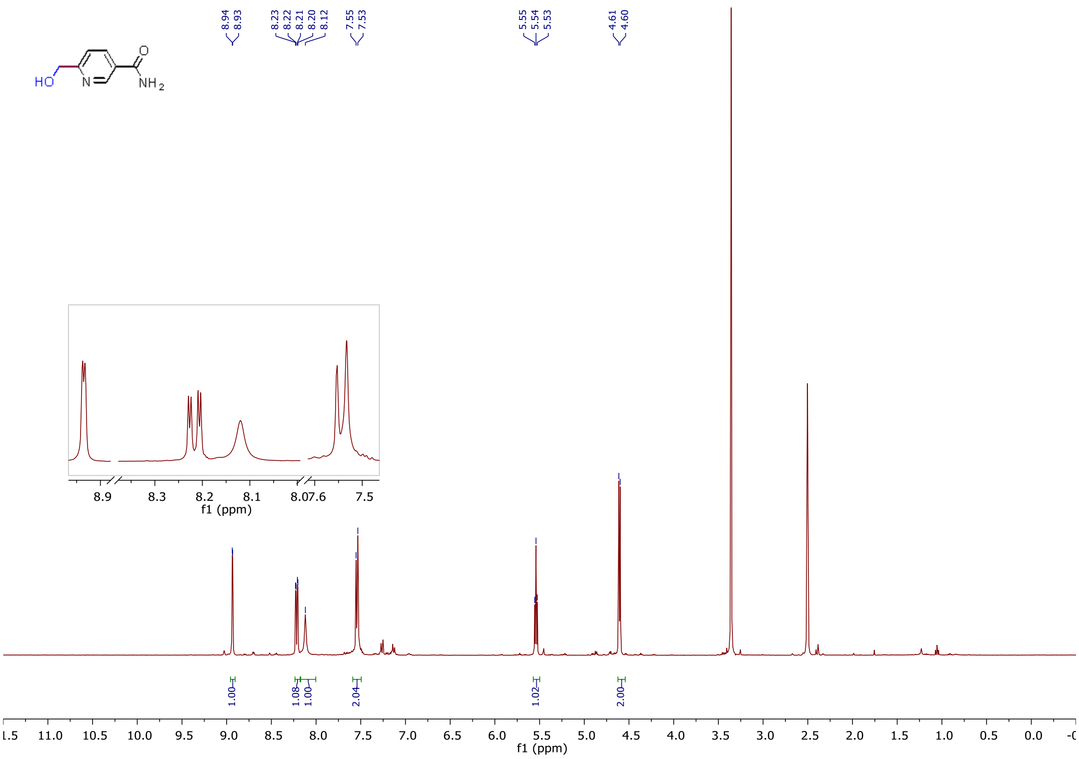

^{13}C NMR (101 MHz, DMSO-d_6) **(21)**

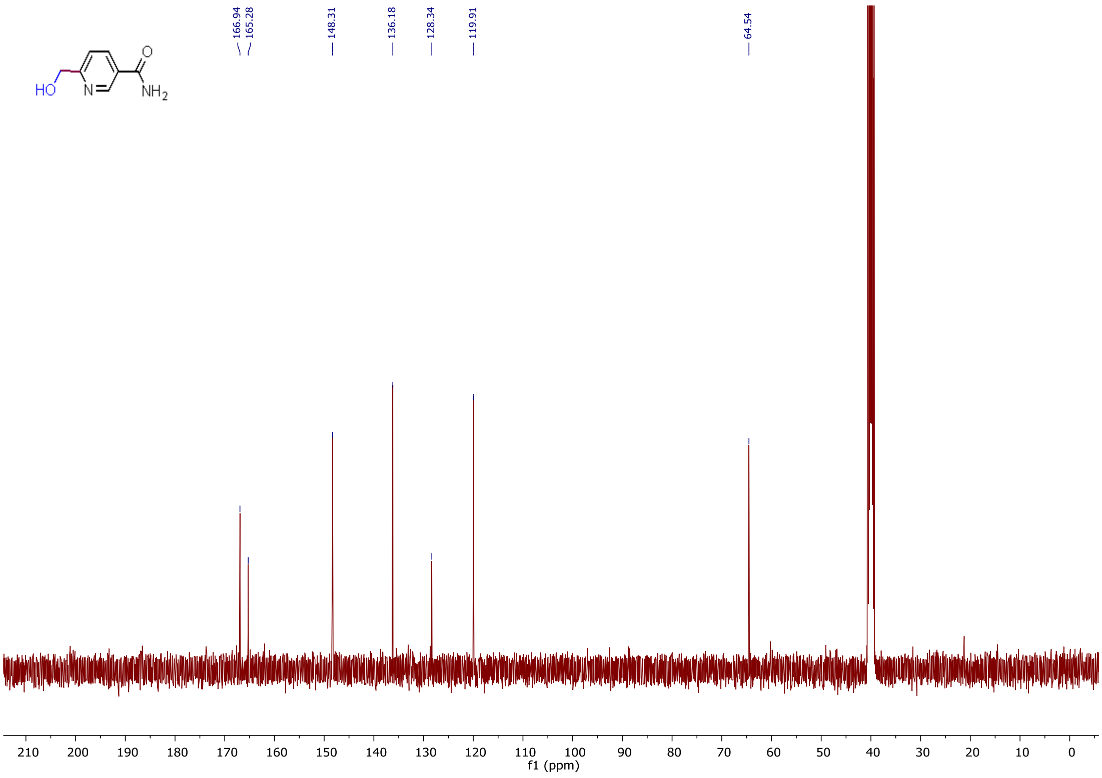

^{1}H NMR (400 MHz, $CDCl_3$) **(22)**

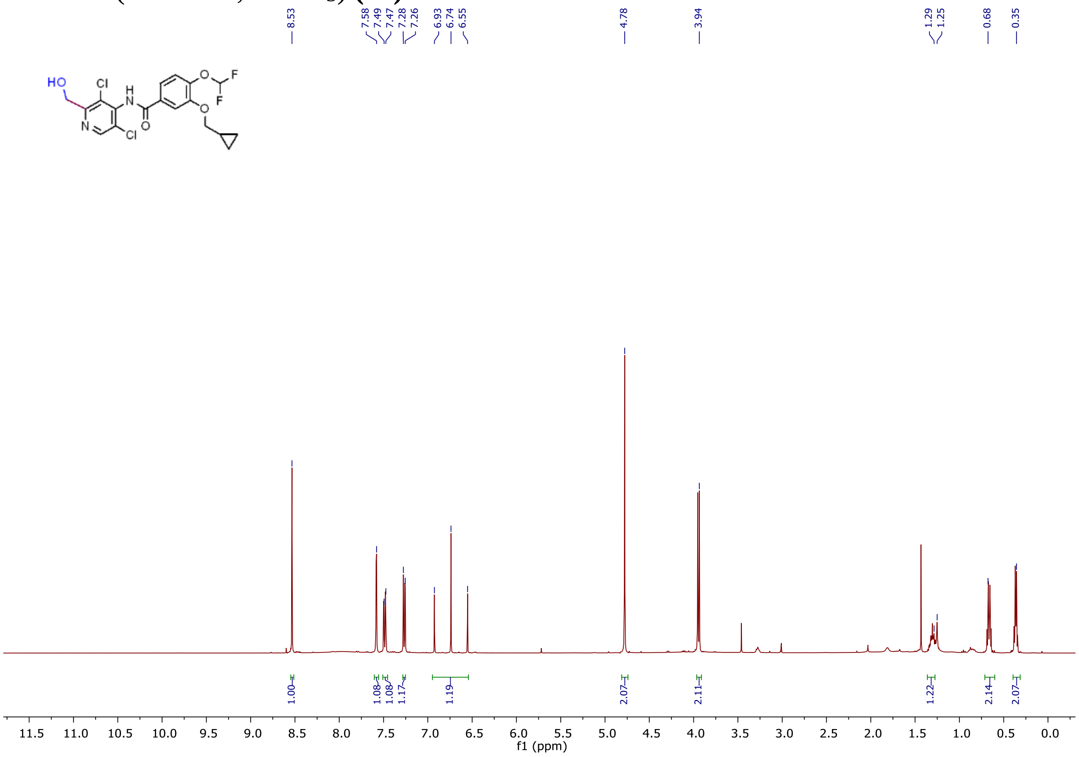

^{13}C NMR (101 MHz, $CDCl_3$) **(22)**

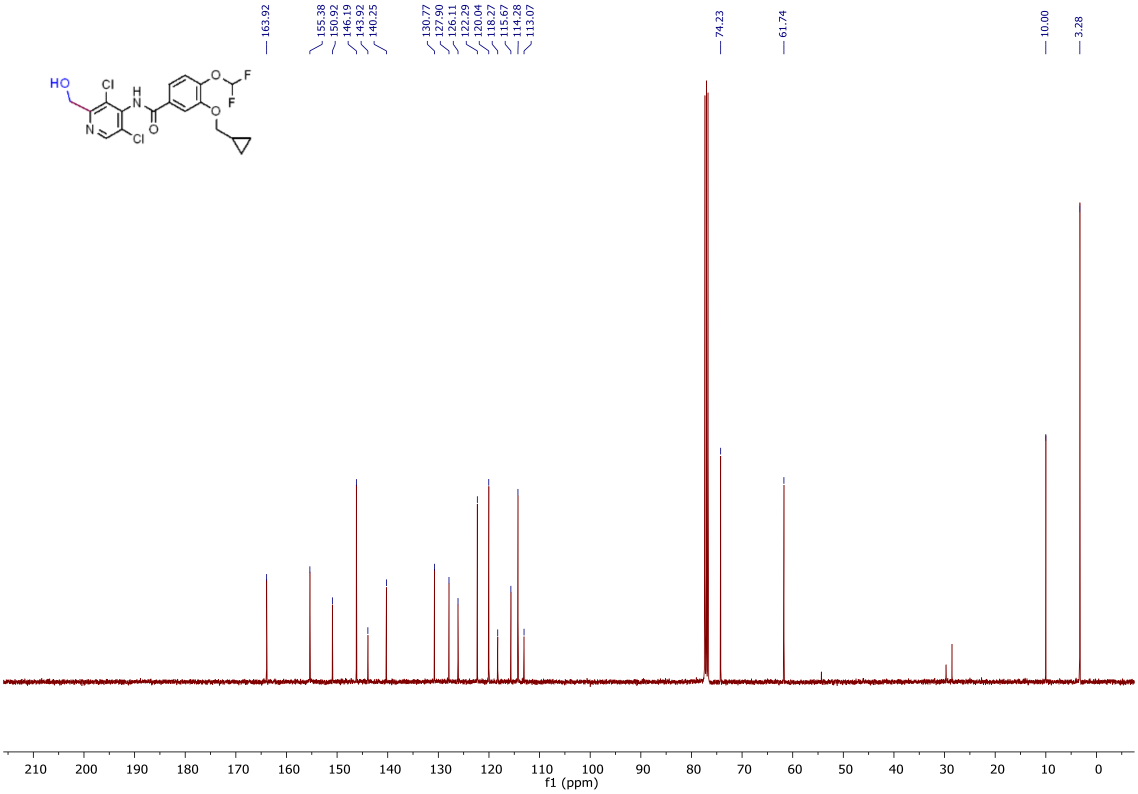

^{19}F NMR (377 MHz, $CDCl_3$) **(22)**

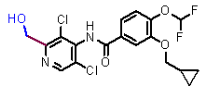

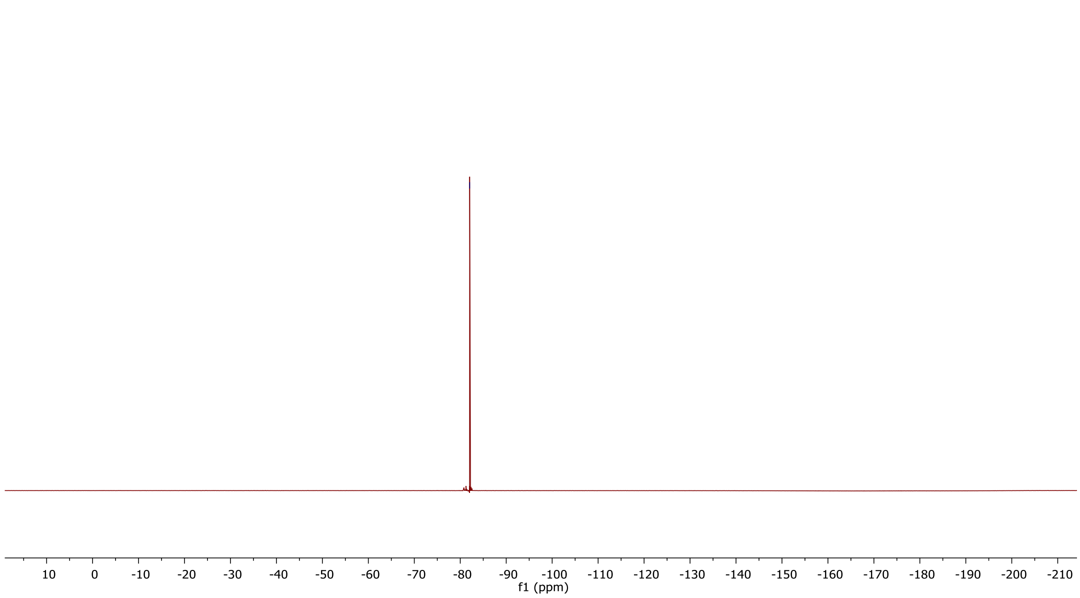

^{1}H NMR (400 MHz, $CDCl_3$) **(23)**

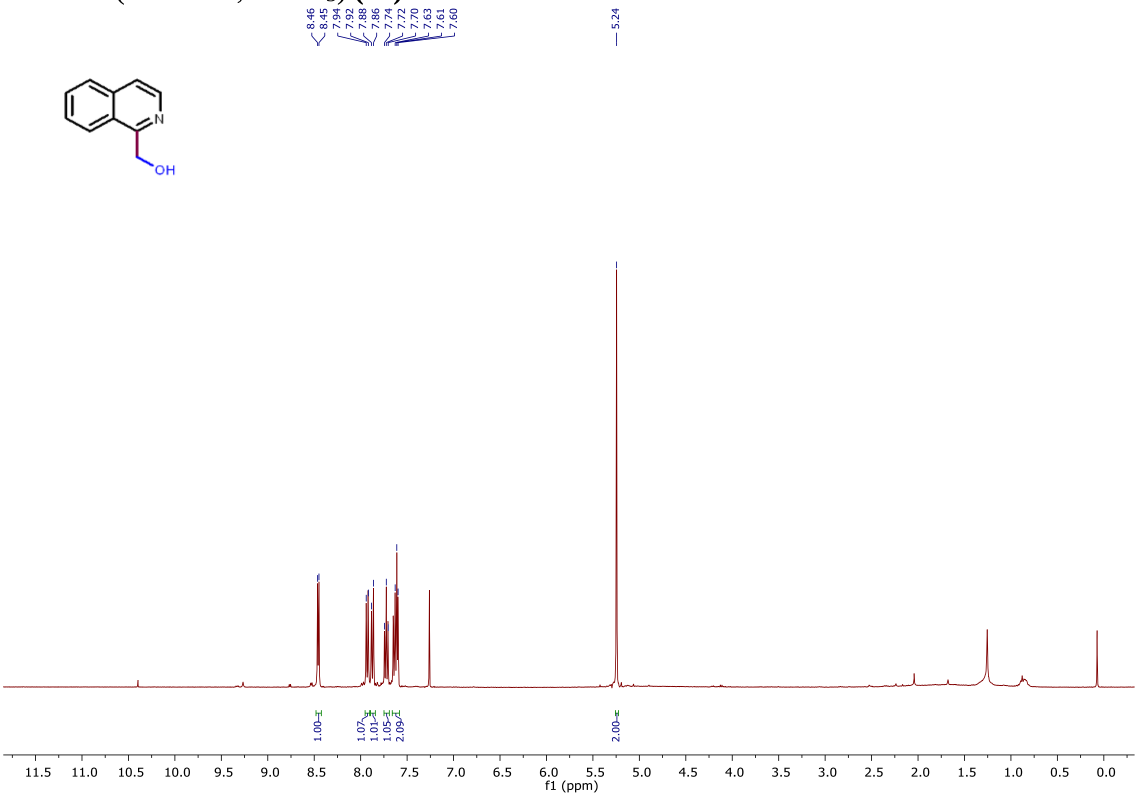

^{13}C NMR (101 MHz, $CDCl_3$) **(23)**

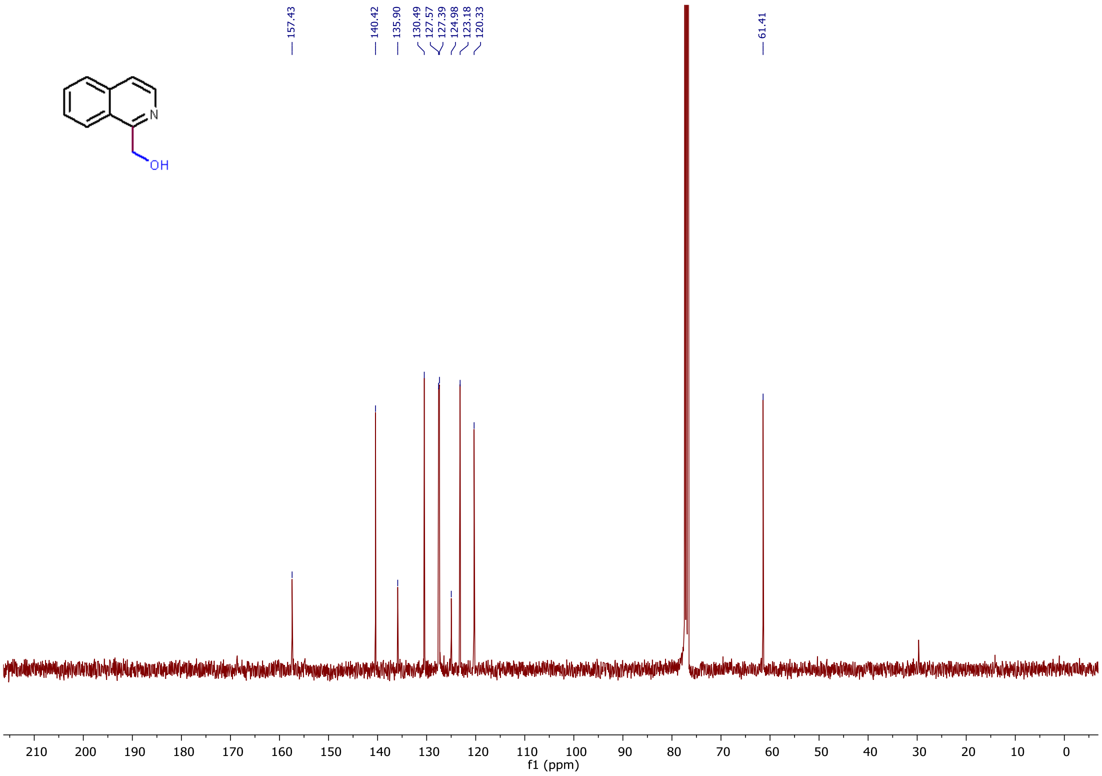

^{1}H NMR (400 MHz, $CDCl_3$) **(24)**

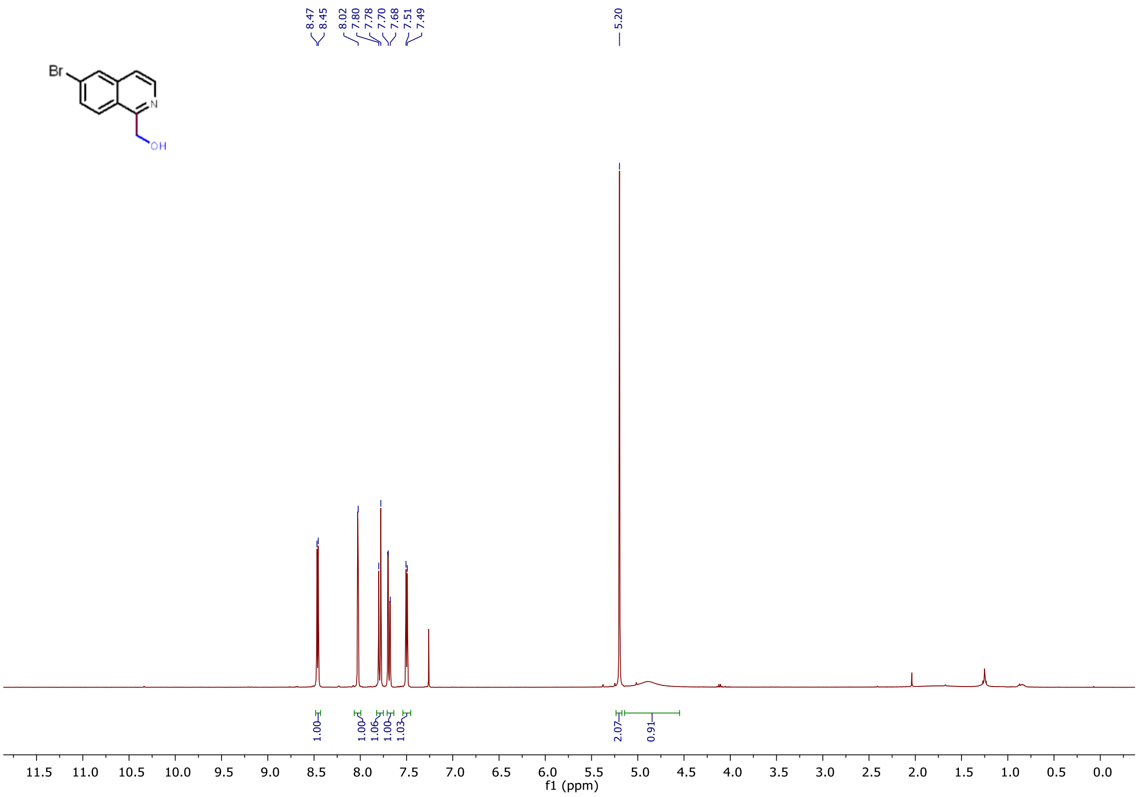

^{13}C NMR (101 MHz, $CDCl_3$) **(24)**

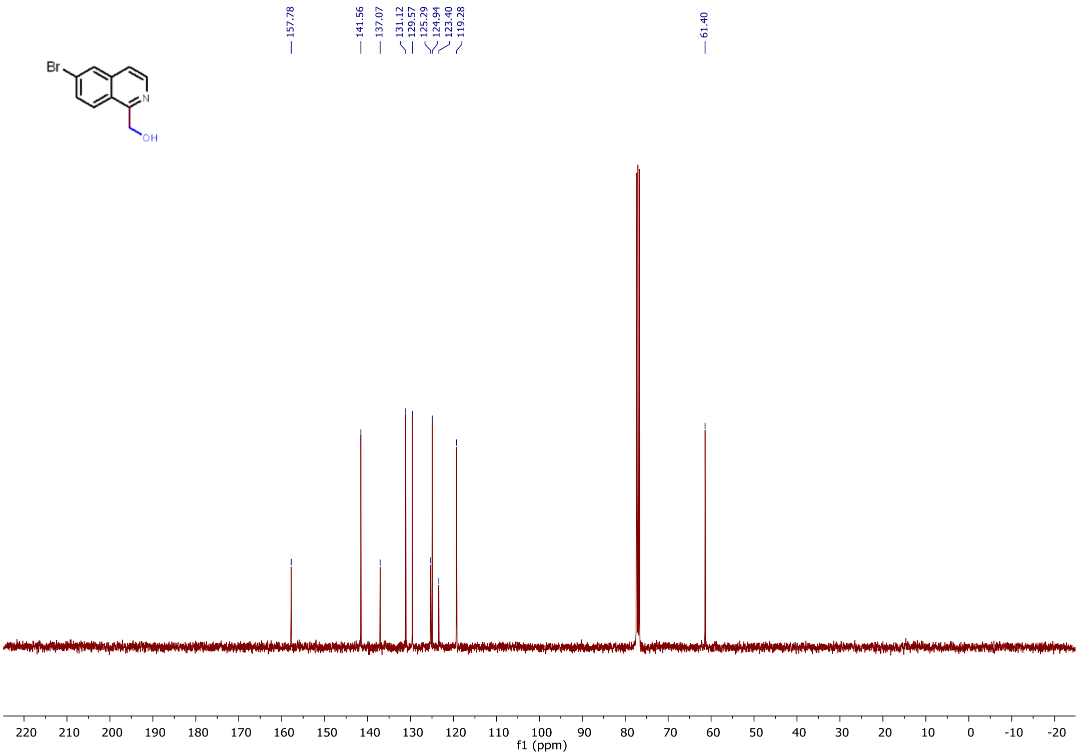

^{1}H NMR (400 MHz, $CDCl_3$) **(25)**

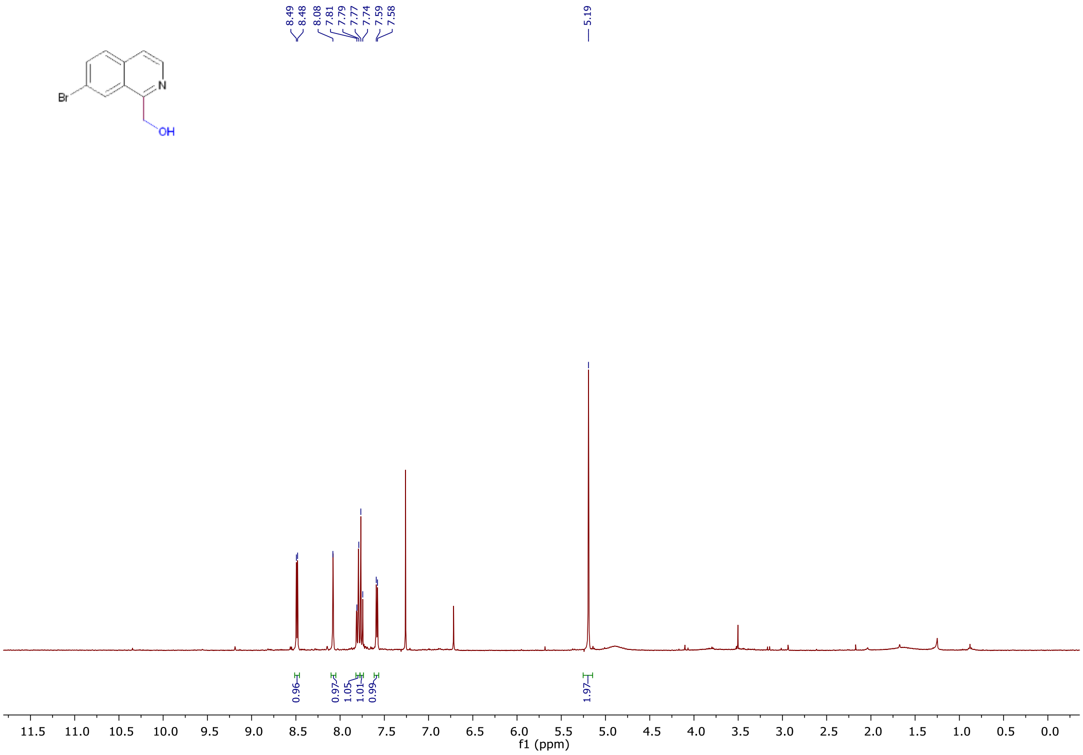

^{13}C NMR (101 MHz, $CDCl_3$) **(25)**

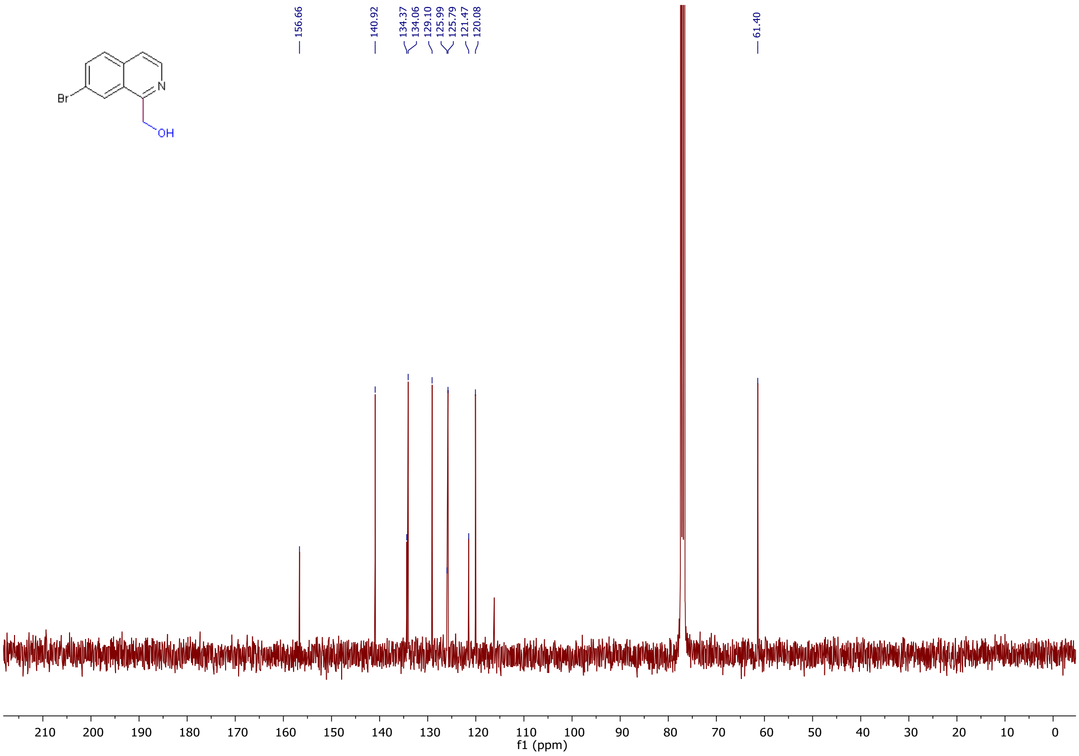

^{1}H NMR (400 MHz, $CDCl_3$) **(26)**

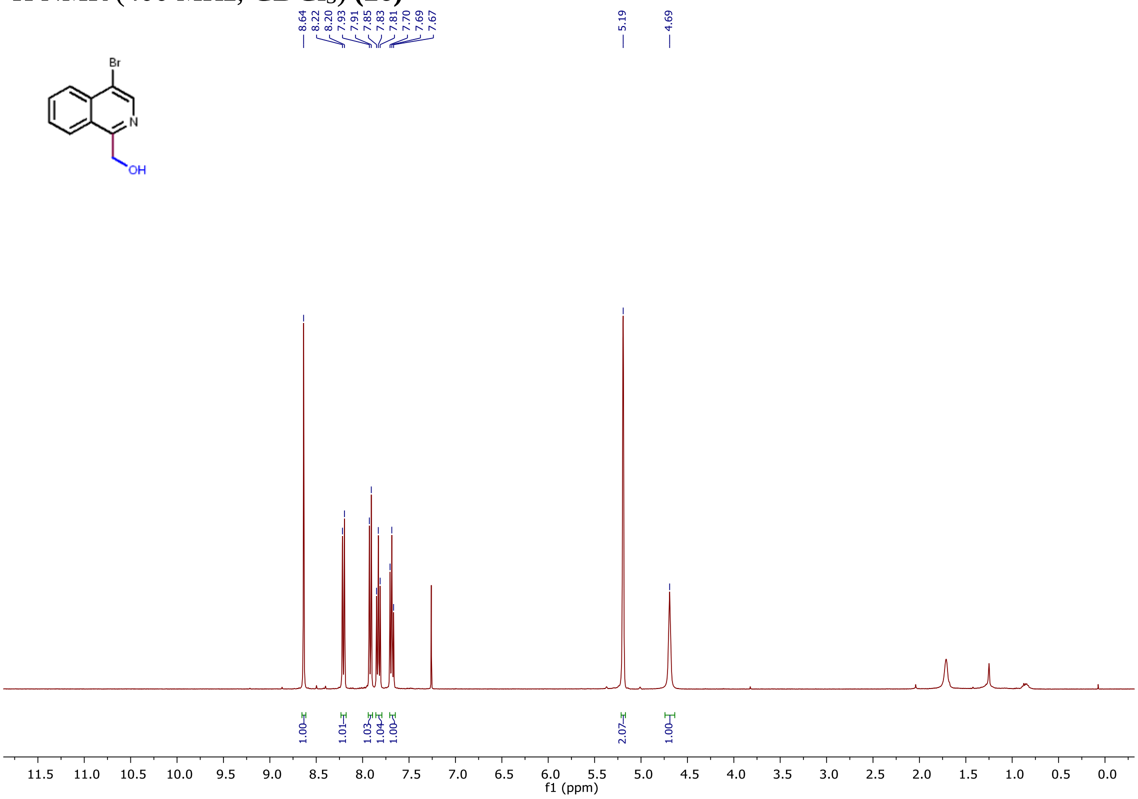

^{13}C NMR (101 MHz, $CDCl_3$) **(26)**

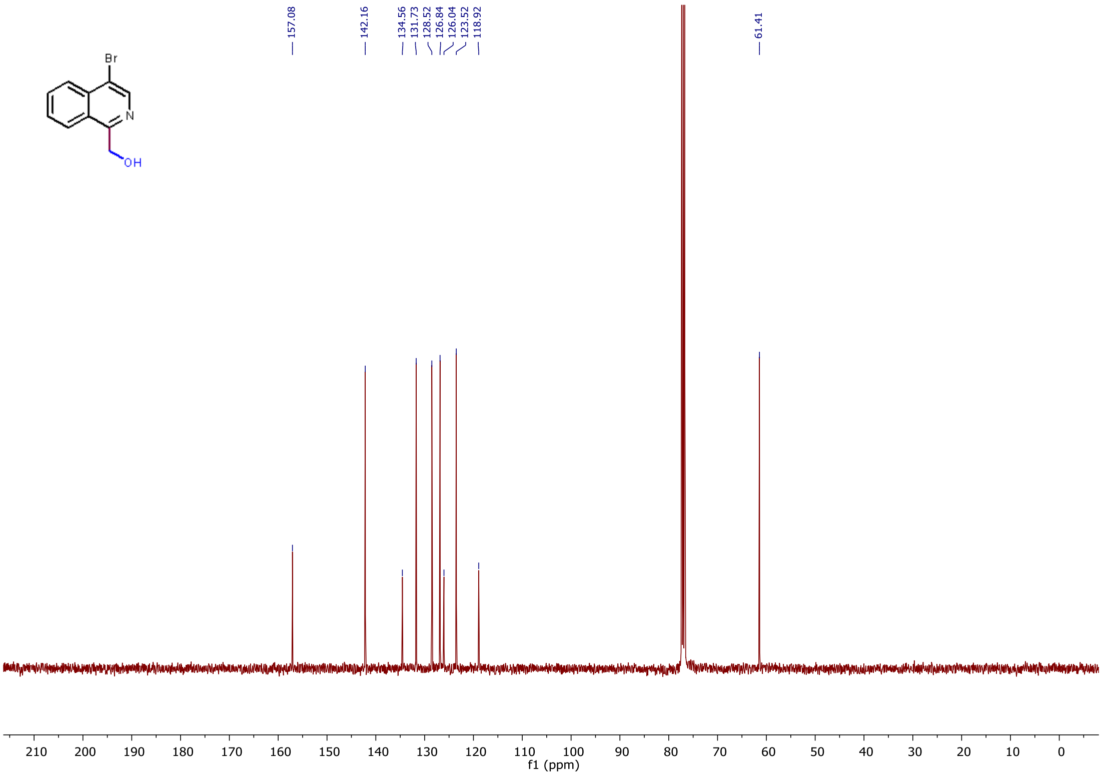

^{1}H NMR (400 MHz, $CDCl_3$) **(27)**

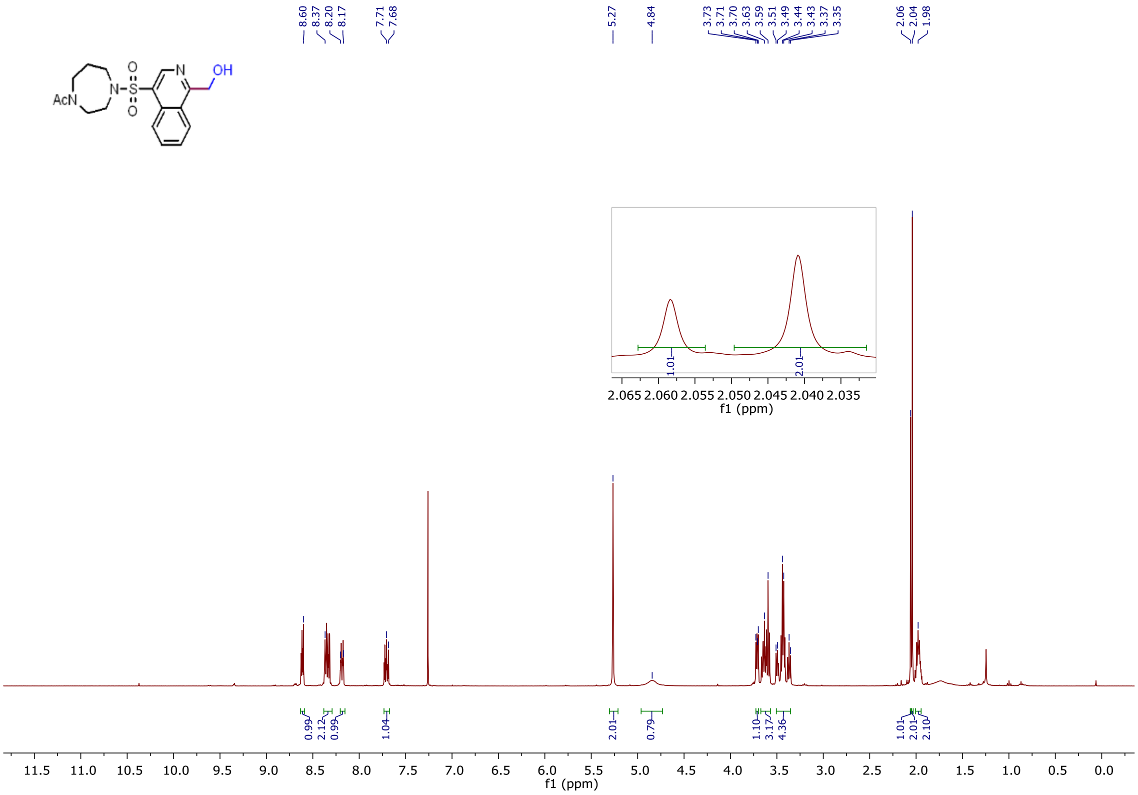

^{13}C NMR (101 MHz, $CDCl_3$) **(27)**

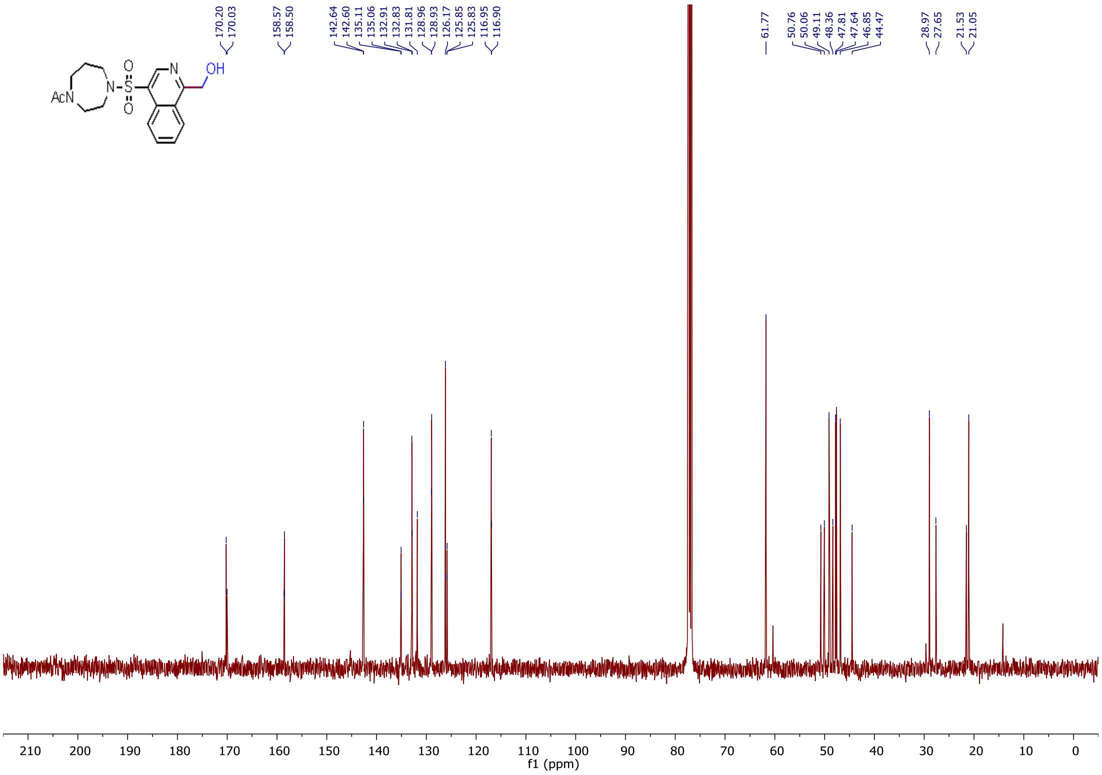

^{1}H NMR (300 MHz, $CDCl_3$) **(28)**

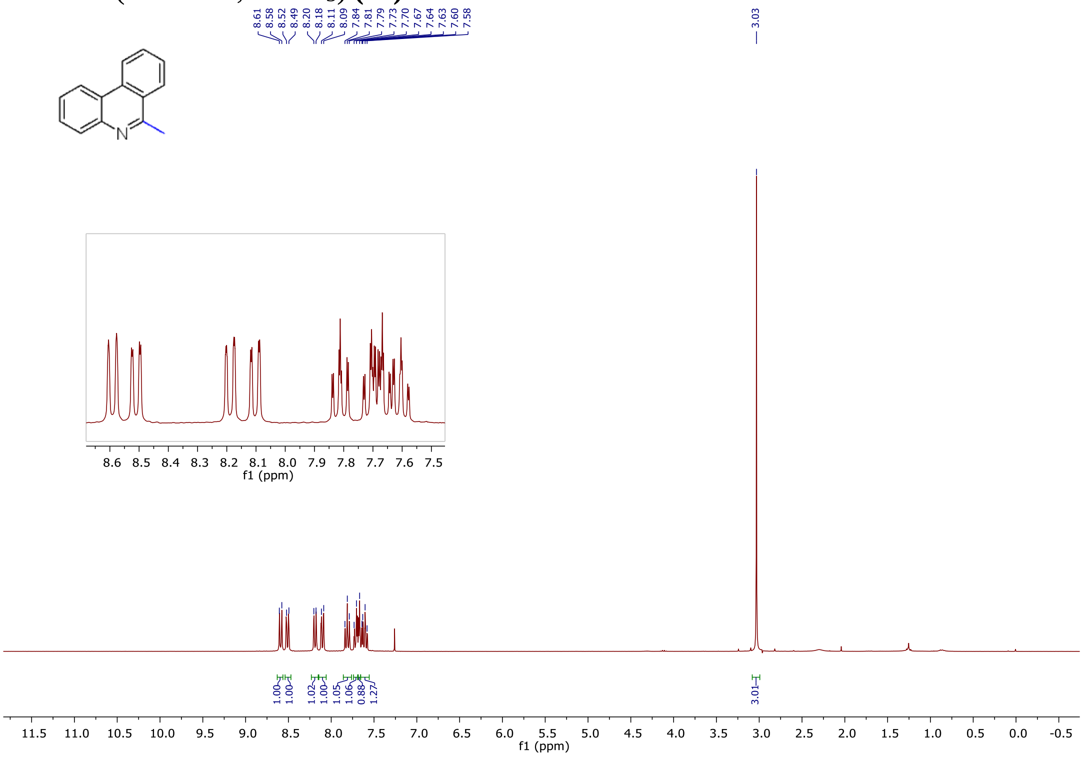

^{13}C NMR (101 MHz, $CDCl_3$) **(28)**

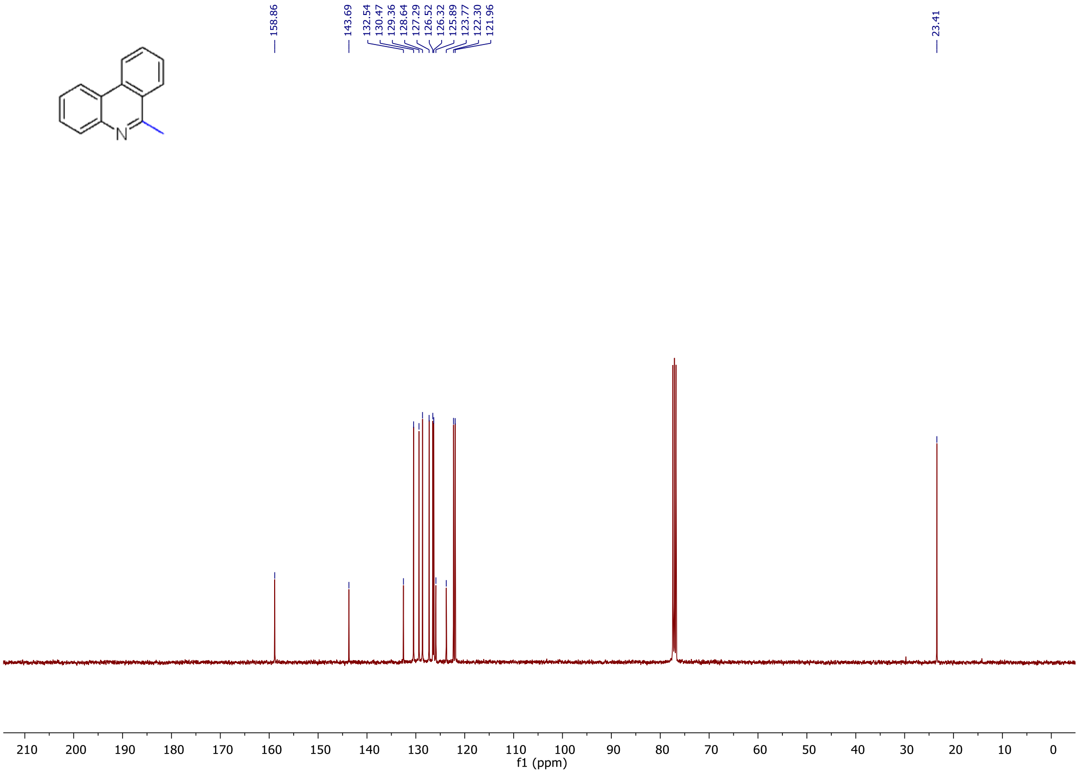

^{1}H NMR (400 MHz, $CDCl_3$) **(29)**

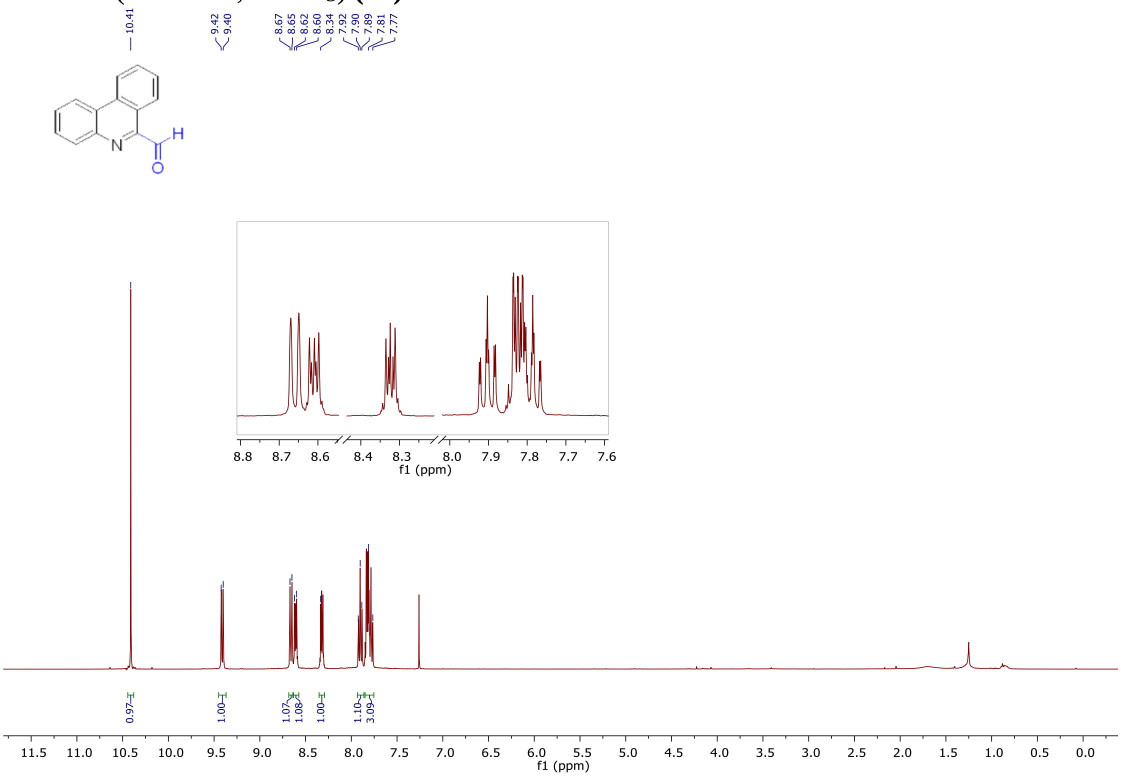

^{13}C NMR (101 MHz, $CDCl_3$) **(29)**

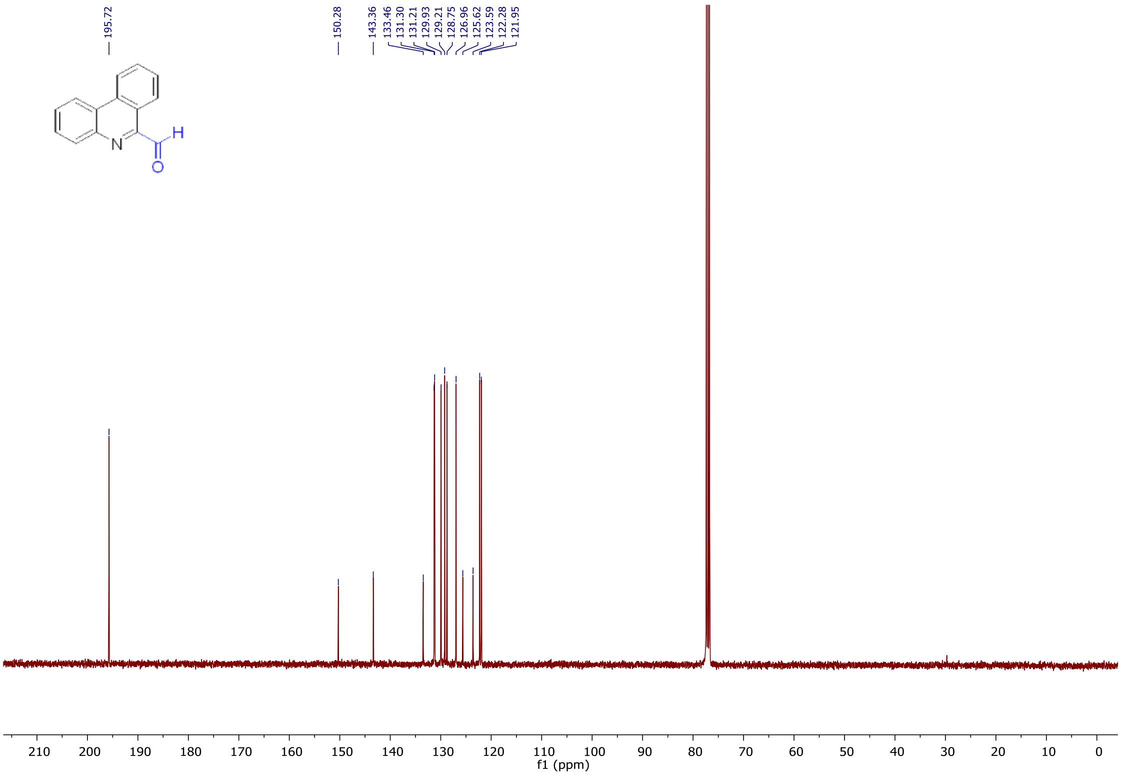

^{1}H NMR (400 MHz, $CDCl_3$) **(30)**

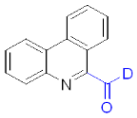

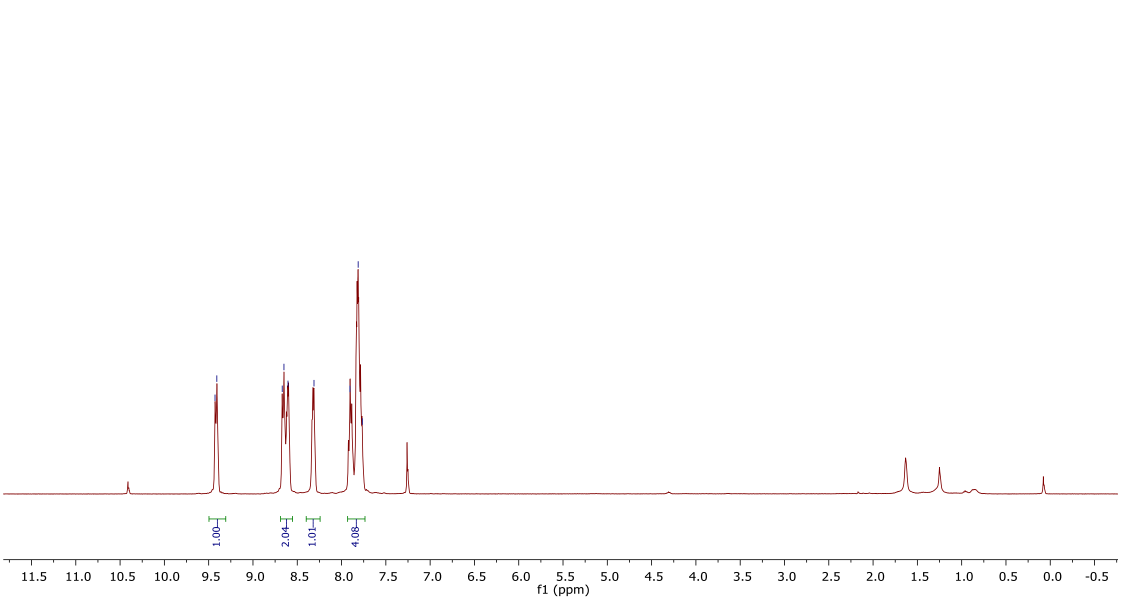

^{13}C NMR (101 MHz, $CDCl_3$) **(30)**

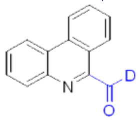

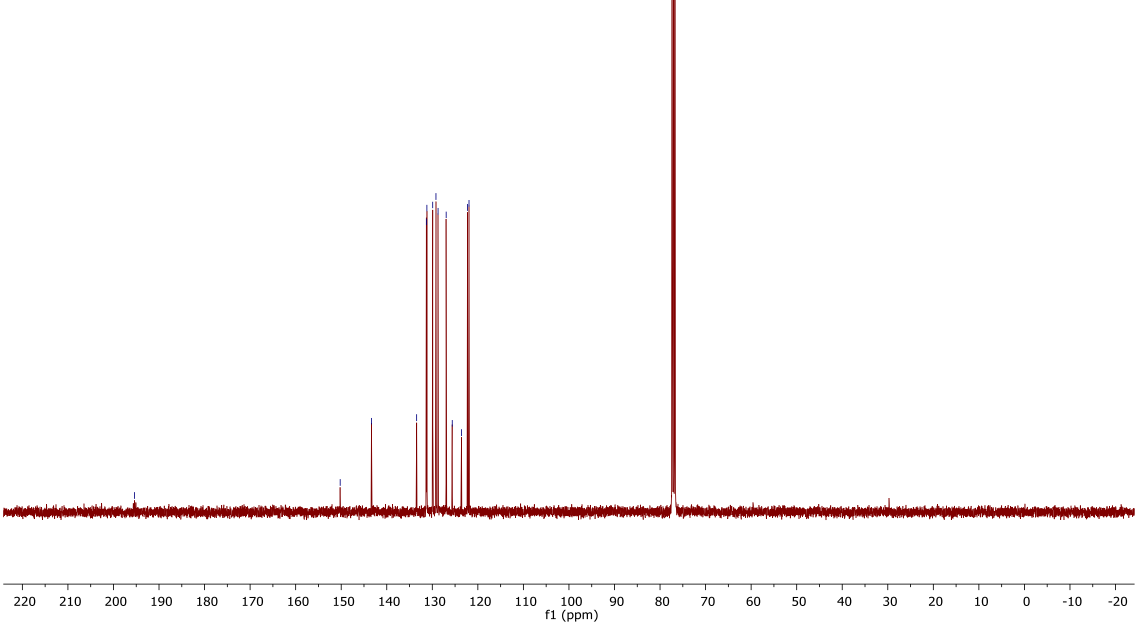

^{1}H NMR (400 MHz, $CDCl_3$) **(31)**

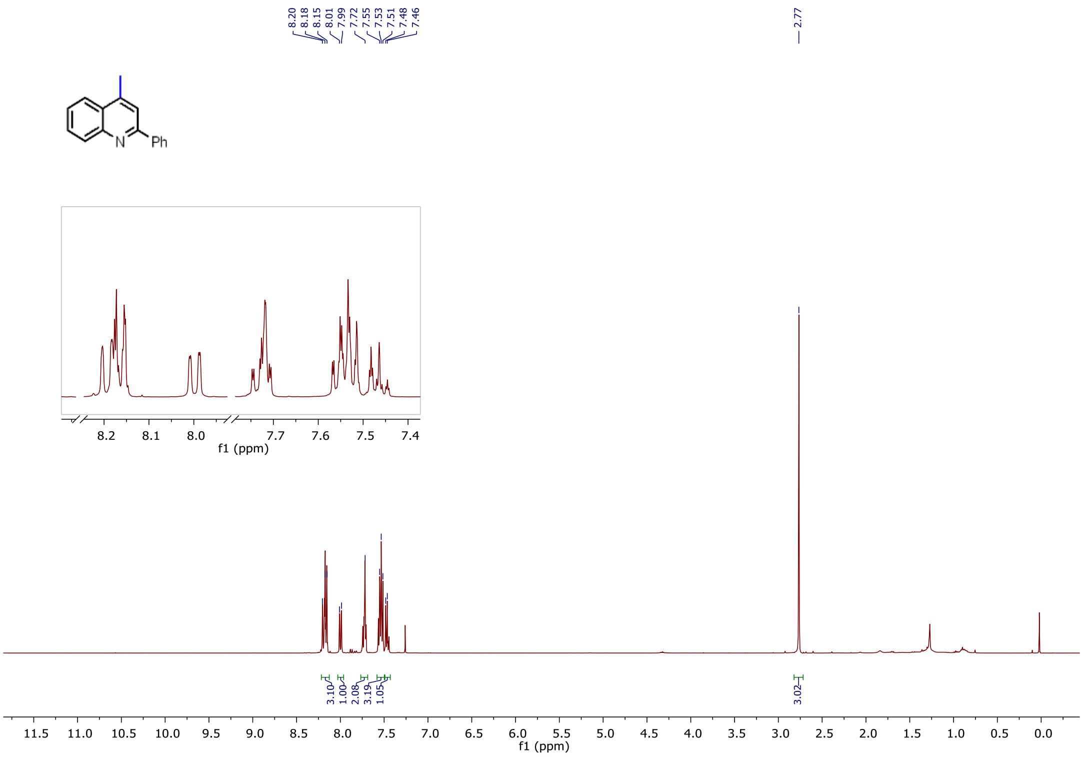

^{13}C NMR (101 MHz, $CDCl_3$) **(31)**

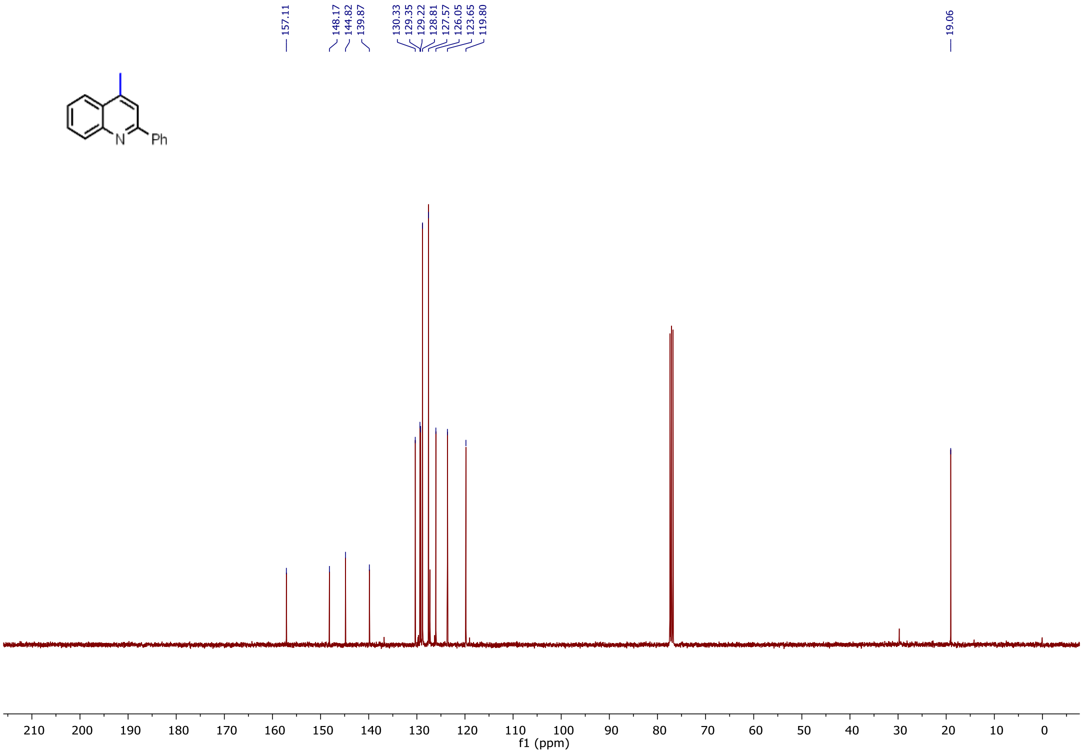

^{1}H NMR (400 MHz, $CDCl_3$) **(32)**

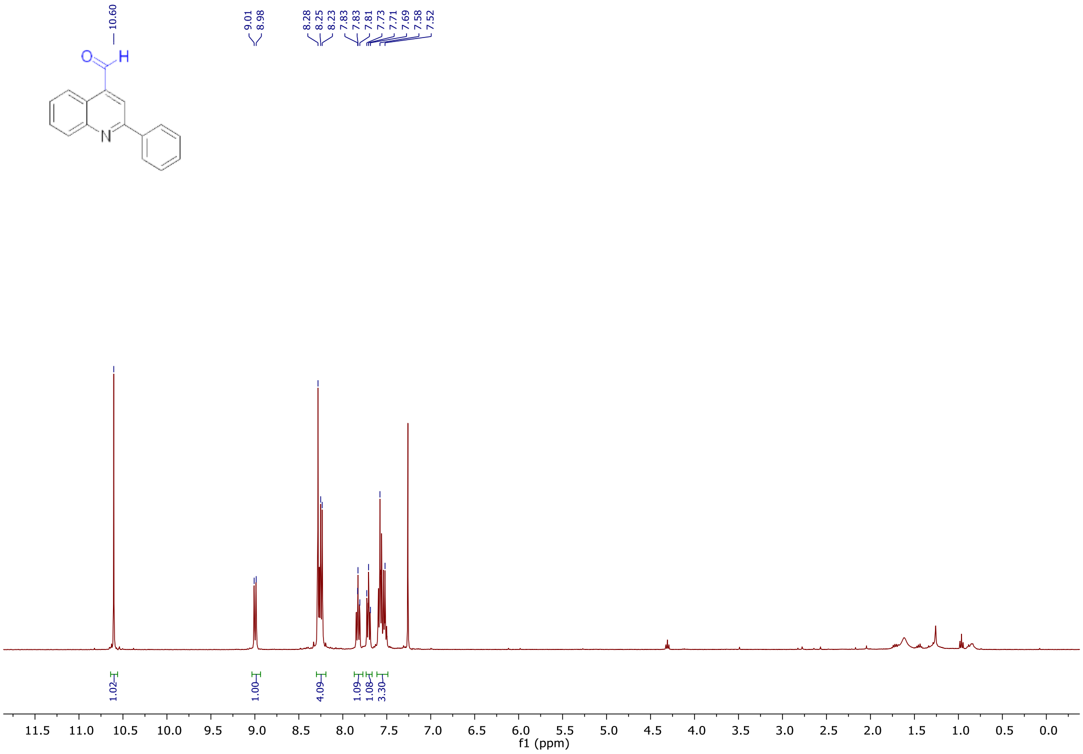

^{13}C NMR (101 MHz, $CDCl_3$) **(32)**

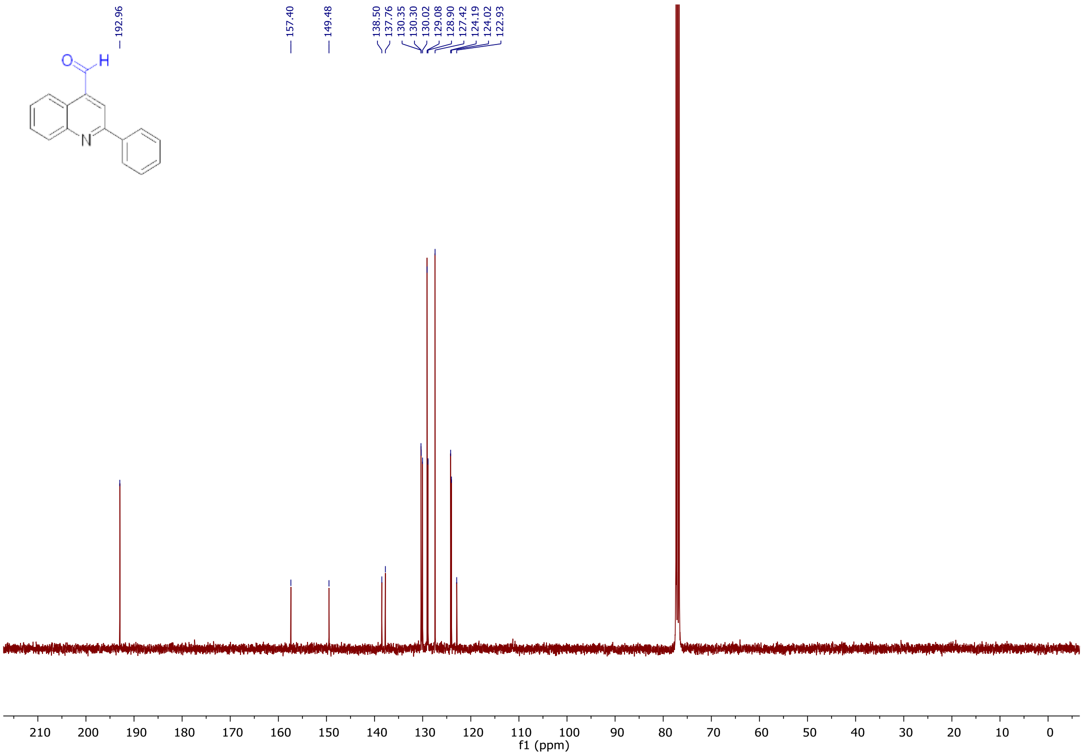

REFERENCES

(1) Laze, L.; Quevedo-Flores, B.; Bosque, I.; Gonzalez-Gomez, J. C. Alkanes in Minisci-Type Reaction under Photocatalytic Conditions with Hydrogen Evolution. *Org. Lett.* **2023**, *25* (48), 8541–8546. .

(2) Ramirez, N. P.; Bosque, I.; Gonzalez-Gomez, J. C. Photocatalytic Dehydrogenative Lactonization of 2-Arylbenzoic Acids. *Org. Lett.* **2015**, *17* (18), 4550–4553.

(3) Proctor, R. S. J.; Davis, H. J.; Phipps, R. J. Catalytic Enantioselective Minisci-Type Addition to Heteroarenes. *Science* **2018**, *360* (6387), 419–422.

(4) Elangovan, A.; Yang, S.-W.; Lin, J.-H.; Kao, K.-M.; Ho, T.-I. Synthesis and Electrogenerated Chemiluminescence of Donor-Substituted Phenylquinolinylethynes and Phenylisoquinolinylethynes: Effect of Positional Isomerism. *Org. Biomol. Chem.,* **2004**, 2, 1597-1602.

(5) Jiang, C.; Liao, Y.; Li, H.; Zhang, S.; Liu, P.; Sun, P.; Electrochemical Silylation of Electron-Deficient Heterocycles Using N-Hydroxyphthalimide as HAT Catalyst. *Adv. Synth. Catal.* **2023**, 365, 1205-1210.

(6) Yang, J.-F.; Liu, Y.-F.; Wei, L.-L.; Qiao, K.-K.; Zhao, Y.-Q.; Shi, L. Minisci-Type Dehydrogenative Coupling of N-Heteroaromatic Rings with Inert C(Sp ^{3})–H Enabled by a Visible-Light-Catalyzed Intermolecular Hydrogen Atom Transfer Process. *J. Org. Chem.* **2024**, *89* (6), 4249–4260.

(7) Kim, J.; Sun, X.; Van Der Worp, B. A.; Ritter, T. Anti-Markovnikov Hydrochlorination and Hydronitrooxylation of α-Olefins via Visible-Light Photocatalysis. *Nat. Catal.* **2023**, *6* (2), 196–203.

(8) Zeng, C.-L.; Wang, H.; Gao, D.; Zhang, Z.; Ji, D.; He, W.; Liu, C.-K.; Yang, Z.; Fang, Z.; Guo, K. CF_3SO_2Na-Mediated Visible-Light-Induced Cross-Dehydrogenative Coupling of Heteroarenes with Aliphatic C(Sp ^{3})–H Bonds. *Org. Lett.* **2022**, *24* (17), 3244–3248.

(9) Niu, L.; Liu, J.; Liang, X.-A.; Wang, S.; Lei, A. Visible Light-Induced Direct α C–H Functionalization of Alcohols. *Nat. Commun.* **2019**, *10* (1), 467.

(10) Zhou, L.; Okugawa, N.; Togo, H. Hydroxymethylation of Quinolines with $Na_2S_2O_8$ by a Radical Pathway. *Eur. J. Org. Chem.* **2017**, *2017* (41), 6239–6245.

(11) Zhang, Y.; Yue, X.; Zhu, J.; Peng, J.; Zhou, C.; Wu, J.; Zhang, P. Visible Light-Induced Hydroxymethylation and Formylation of (Iso)Quinolines with Alcohols. *Mol. Catal.* **2022**, *530*, 112594..

(12) Stenkamp, D.; Mueller, S. G.; Lustenberger, P. **(2005)**. Alkyne compounds with mch antagonistic activity and medicaments comprising these compounds. (U.S. Patent No. 7,592,358 B2).

(13) Rammal, F.; Gao, D.; Boujnah, S.; Hussein, A. A.; Lalevée, J.; Gaumont, A.-C.; Morlet-Savary, F.; Lakhdar, S. Photochemical C–H Silylation and Hydroxymethylation of Pyridines and Related Structures: Synthetic Scope and Mechanisms. *ACS Catal.* **2020**, *10* (22), 13710–13717.

(14) Uhlig, N.; Martins, A.; Gao, D. Selective DIBAL-H Monoreduction of a Diester Using Continuous Flow Chemistry: From Benchtop to Kilo Lab. *Org. Process Res. Dev.* **2020**, *24* (10), 2326–2335.

(15) Xu, P.; Chen, P.; Xu, H. Scalable Photoelectrochemical Dehydrogenative Cross-Coupling of Heteroarenes with Aliphatic C−H Bonds. *Angew. Chem. Int. Ed.* **2020**, *59* (34), 14275–14280.

(16) Jin, J.; MacMillan, D. W. C. Alcohols as Alkylating Agents in Heteroarene C–H Functionalization. *Nature* **2015**, *525* (7567), 87–90.

(17) Xu, Z.; Zhang, L. Methanol as a Formylating Agent in Nitrogen Heterocycles. *Org. Biomol. Chem.* **2021**, *19* (43), 9476–9482.

(18) Shantharjun, B.; Vani, D.; Unnava, R.; Sandeep, M.; Reddy, K. R. Hydroxymethylation of Quinolines *via* Iron Promoted Oxidative C–H Functionalization: Synthesis of Arsindoline-A and Its Derivatives. *Org. Biomol. Chem.* **2021**, *19* (3), 645–652.

(19) Dong, J.; Wang, X.; Song, H.; Liu, Y.; Wang, Q. Photoredox-Catalyzed Redox-Neutral Minisci C−H Formylation of N-Heteroarenes. *Adv. Synth. Catal.* **2020**, *362* (11), 2155–2159.
